# Comparative Panel Sequencing of DNA Variants in cf-, ev- and tumorDNA for Pancreatic Ductal Adenocarcinoma Patients

**DOI:** 10.3390/cancers14041074

**Published:** 2022-02-21

**Authors:** Mareike Waldenmaier, Lucas Schulte, Jonathan Schönfelder, Axel Fürstberger, Johann M. Kraus, Nora Daiss, Tanja Seibold, Mareen Morawe, Thomas J. Ettrich, Hans A. Kestler, Christoph Kahlert, Thomas Seufferlein, Tim Eiseler

**Affiliations:** 1 Department of Internal Medicine, University Clinic Ulm, 89081 Ulm, Germany; mareike.waldenmaier@uniklinik-ulm.de (M.W.); lucas-alexander.schulte@uniklinik-ulm.de (L.S.); jonathan.schoenfelder@uniklinik-ulm.de (J.S.); nora.daiss@uniklinik-ulm.de (N.D.); tanja.seibold@uniklinik-ulm.de (T.S.); mareen.morawe@uniklinik-ulm.de (M.M.); thomas.ettrich@uniklinik-ulm.de (T.J.E.); 2 Institute of Medical Systems Biology, University Ulm, 89081 Ulm, Germany; axel.fuerstberger@uni-ulm.de (A.F.); johann.kraus@uni-ulm.de (J.M.K.); hans.kestler@uni-ulm.de (H.A.K.); 3 Department of General-, Visceral- and Vascular Surgery, University Clinic Carl Gustav Carus Dresden, 01307 Dresden, Germany; christoph.kahlert@uniklinikum-dresden.de

**Keywords:** liquid biopsy, PDAC, extracellular vesicles, exosomes, cfDNA, next-generation sequencing

## Abstract

**Simple Summary:**

Pancreatic ductal adenocarcinoma (PDAC) has still a dismal prognosis. To improve treatment, personalized medicine uses next-generation DNA sequencing to monitor disease and guide treatment decisions. Tumor samples for sequencing are usually obtained by invasive fine-needle biopsy. Recently, the focus has been increasingly shifting to blood-based liquid biopsies, including circulating free (cf)DNA or DNA isolated from extracellular vesicles (evDNA). To evaluate the detection performance of DNA alterations, we directly compared tumor-, cf- and evDNA from patients with advanced PDAC upon panel sequencing. Copy number variations (CNVs), single nucleotide variants (SNVs) and insertions and deletions (indels) were compared for their concordance with tumorDNA. Compared to cfDNA, evDNA contained significantly larger DNA fragments, which improved the concordance of SNVs and indels with tumorDNA. In line with previous observations, CNV detection was mostly uninformative for cf- and evDNA. However, the combination of both liquid biopsy analytes was clearly superior for SNV detection, pointing to potentially improved actionable variant prediction.

**Abstract:**

Pancreatic ductal adenocarcinomas (PDACs) are tumors with poor prognosis and limited treatment options. Personalized medicine aims at characterizing actionable DNA variants by next-generation sequencing, thereby improving treatment strategies and outcomes. Fine-needle tumor biopsies are currently the gold standard to acquire samples for DNA profiling. However, liquid biopsies have considerable advantages as they are minimally invasive and frequently obtainable and thus may help to monitor tumor evolution over time. However, which liquid analyte works best for this purpose is currently unclear. Our study aims to directly compare tumor-, circulating free (cf-) and extracellular vesicle-derived (ev)DNA by panel sequencing of matching patient material. We evaluated copy number variations (CNVs), single nucleotide variants (SNVs) and insertions and deletions (indels). Our data show that evDNA contains significantly larger DNA fragments up to 5.5 kb, in line with previous observations. Stringent bioinformatic processing revealed a significant advantage of evDNA with respect to cfDNA concerning detection performance for SNVs and a numerical increase for indels. A combination of ev- and cfDNA was clearly superior for SNV detection, as compared to either single analyte, thus potentially improving actionable variant prediction upon further optimization. Finally, calling of CNVs from liquid biopsies still remained challenging and uninformative.

## 1. Introduction

Pancreatic cancer is characterized by a dismal prognosis due to late-stage diagnosis and early metastasis, with an overall 5-year survival rate of less than 9% [1,2]. The most prevalent tumor subtype is ductal adenocarcinoma (PDAC) [3]. Owing to their aggressive nature with high inter- and intracellular heterogeneity and an abundant desmoplastic microenvironment, PDACs are rather resistant towards not only conventional treatment efforts, including chemo- and radiotherapy, but also targeted agents and immunotherapies. Thus, new treatment options are urgently needed [4,5,6]. Personalized medicine is increasingly implemented in clinical oncology, aiming at advancing tumor diagnosis and treatment [7]. Personalized medicine approaches often utilize next-generation sequencing (NGS) of tumor tissue to determine actionable variants in tumors and to tailor therapeutic strategies [8]. In PDAC, mainly fine-needle biopsies of the primary tumor or metastases are performed, which are stressful for patients particularly when performed several times and can represent only a snapshot of the tumor at the biopsy site due to intratumoral heterogeneity [9]. Therefore, the focus shifts from tumor to liquid biopsies, utilizing analytes mainly isolated from peripheral blood, including circulating cell-free (cf) DNA, circulating tumor cells (CTCs) and extracellular vesicles (EVs), such as exosomes (small extracellular vesicles (sEVs)) that also contain tumor DNA fragments [10]. In contrast to tumor biopsies, liquid biopsies can be obtained easily, minimally invasively and therefore repeatedly and thus enable longitudinal analyses during treatment [11]. Copy number variations (CNVs), insertions and deletions (indels) and single nucleotide variants (SNVs) were already successfully detected both in cfDNA as well as in DNA isolated from EVs (evDNA) [8,11,12]. Low fractions of EVs and cfDNA from the tumor can often hamper concordant variant calling and make screening for clinically relevant variants a challenging effort, as shown for tumor-derived cfDNA by Elazezy et al. [13]. In particular, CNVs, which represent major genetic alterations commonly observed across various solid tumor entities, are difficult to predict in cf- as well as evDNA, due to a mixture of analytes from different cells of origin [14]. cfDNA was suggested to exhibit higher sensitivity for CNV detection than evDNA in some studies [15]. NGS sequencing approaches utilizing cfDNA, but in particular EVs, are yet not part of the clinical routine. Most of the research studies so far examined only specific, known variants using highly sensitive digital droplet PCR (ddPCR). Here, analysis of evDNA was reported to be superior to cfDNA; e.g., detection rates for mutated KRAS alleles were higher in evDNA than in cfDNA in early-stage pancreatic cancer patients [16]. Moreover, evDNA fragments perform better with respect to amplification for NGS applications [17]. This may be explained by larger DNA fragment sizes in EVs of up to 10 kilobases (kb), whereas cfDNA mainly consists of short 167 bp fragments [18,19]. cfDNA from the tumor can be even more fragmented with a length <100 bp, thus impairing reliability of sequencing data [20]. evDNA also has other potential advantages including high stability of nucleic acids due to protection within the lipid bilayer of the nanovesicles, as well as the ability for multiplexing different analytes, such as proteins and DNA or different RNA classes [21,22]. However, there are caveats that so far prevent the widespread use of evDNA analysis, such as the more complex isolation and characterization procedures for EVs as well as lower DNA yields in comparison to cfDNA. By analyzing a cohort of patients with advanced PDAC, we directly compared cfDNA and evDNA isolated from plasma samples by panel sequencing with DNA extracted from tumor tissue from the same patients obtained by fine-needle biopsy.

## 2. Materials and Methods

### 2.1. EV Preparation/Sample Processing

Liquid biopsy plasma samples and fine-needle biopsies were obtained from 10 patients with advanced PDAC treated in the outpatient clinic of the Department of Internal Medicine I, Ulm University, Ulm, Germany. All patients had given prior written informed consent before samples were taken according to the institutional ethical approval number 67/19. In addition, blood from healthy probands was obtained with informed consent. Until processing within 1 h, monovettes (Sarstedt) containing peripheral blood and EDTA were stored at 4 °C. To separate blood plasma from whole blood, samples were centrifuged (820× *g*, 10 min, 4 °C) and plasma was transferred into precooled tubes. Then, plasma was again centrifuged (20,000× *g*, 10 min, 4 °C), transferred into precooled cryovials and immediately stored at −80 °C, until further processing. Subsequently, plasma samples were thawed on ice, and EVs were isolated from plasma using the Total Exosome Isolation Kit (from plasma) (Thermo Fisher Scientific, Waltham, MA, USA). Subsequently, plasma was centrifuged for 20 min at 2000× *g* at room temperature (RT) and the supernatant was transferred to a new tube. Then plasma was centrifuged for 20 min at 10,000× *g* at RT. After the transfer of supernatant to a new tube, 0.5 volumes of PBS (1×) were added, and plasma was mixed thoroughly by vortexing. Then, 0.2 volumes of the total sample volume of Exosome Precipitation reagent (from plasma) were added and the sample was mixed thoroughly by vortexing, followed by incubation for 10 min at RT. After centrifugation for 5 min at 10,000× *g* at RT, the supernatant was discarded and samples were centrifuged for 30 s at 10,000× *g* at RT. The resolving supernatant was discarded again, and the EV pellet was frozen at −80 °C until further processing.

evDNA and cfDNA were prepared using the QIAamp Circulating Nucleic Acid Kit (Qiagen, Hilden, Germany) as described in the manufacturer’s instructions. EV pellets for evDNA and 4 mL of plasma for cfDNA were resuspended in 4 mL of PBS and 400 µL of proteinase K, respectively. At the end, samples were eluted in 30 µL of Buffer AVE, and DNA concentration was measured using Qubit dsDNA HS Assay Kit (Thermo Fisher Scientific) according to the manufacturer’s instructions. DNA isolated from fine-needle tumor biopsies from all patients was obtained from the Department of Pathology of the University Clinic Ulm.

### 2.2. DNA Quality and Sequencing

**Figure 1:**
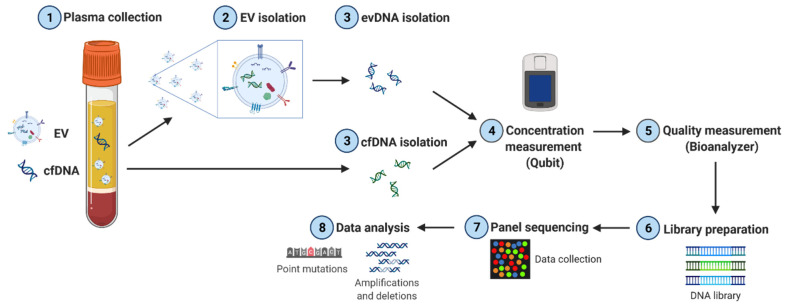
Experimental workflow of sample and data processing. EVs were isolated from PDAC patient plasma by precipitation. ev- and cfDNA were prepared and DNA quality as well as fragment length were determined using a bioanalyzer device. Sequencing was performed using a commercial panel covering 766 tumor-relevant genes.

After isolation, tumor-, ev- and cfDNA samples were sent for commercial sequencing with the somatic TUM01-panel covering 766 tumor-relevant genes (CeGaT GmbH, Tübingen, Germany). This panel contains only coding regions for the majority of targets (Table S1). For some genes, intronic regions were included to allow for the detection of translocations as listed in Table S1. Furthermore, intronic regions were partially enriched to cover known pathogenic variants from ClinVar and HGMD. Prior to sequencing, DNA quality was measured using a bioanalyzer, followed by library preparation with 50 ng of DNA. Sequencing was performed on a NovaSeq 6000 system (Illumina, 2 × 100 bp). The initial bioinformatic analysis was performed by CeGaT (Figure 1).

Sequencing reads were demultiplexed with Illumina bcl2fastq (2.20) and adapters were trimmed using Skewer (version 0.2.2), but no quality trimming of the reads was performed. To prevent removal of reads that map to pseudoautosomal regions (PARs) on the Y chromosome in the hg19-cegat reference genome, the respective chromosomal positions (chrY:10001-2649520, chrY:59034050-59363566) were masked. The ABRA tool was used to facilitate global accurate alignment of reads to target regions and more precise indel calling [23]. In addition, a proprietary software tool (CeGaT) was used to discard reads aligning to more than one locus, showing duplicated reads and the same mapping score, which in all probability originated from the same PCR amplicon.

#### 2.2.1. Variant Calling

CNVs were detected by overlapping reads with specific target regions and compared to the expected number in a proprietary reference sample cohort (CeGaT) [24]. To determine the number of CNVs on chromosomes X and Y, sex of the patients was estimated according to the coverage, to analyze the expected copy number. Then, variants were annotated based on various public databases (Ensembl v100, RefSeq Curated (20200723), CCDS r22, dbSNP154, GnomAD 2.1.1 (exonic) and 3.1 (genomic), Gencode 34). Regions were reported as homozygous deletions upon a decrease in coverage to less than 5%, as heterozygous deletions with a coverage of less than 55% and as duplications if the coverage was more than 145% of the expected value. Since coding regions are usually analyzed by the TUM01 panel, CNV detection is limited to exons, and precise start or end coordinates were not provided. If all exons of a gene are affected, the whole gene was labeled as changed, even though technically no information about introns was available. In the case of larger chromosomal deletions, all affected genes were reported individually. Following this annotation (Files S1–S30), the numbers of CNV variants for tumor-, ev- and cfDNA per patient were determined, and CNVs were analyzed in relation to the term “call”, which depicts for duplication, homo- or heterozygous deletion.

**Figure 2:**
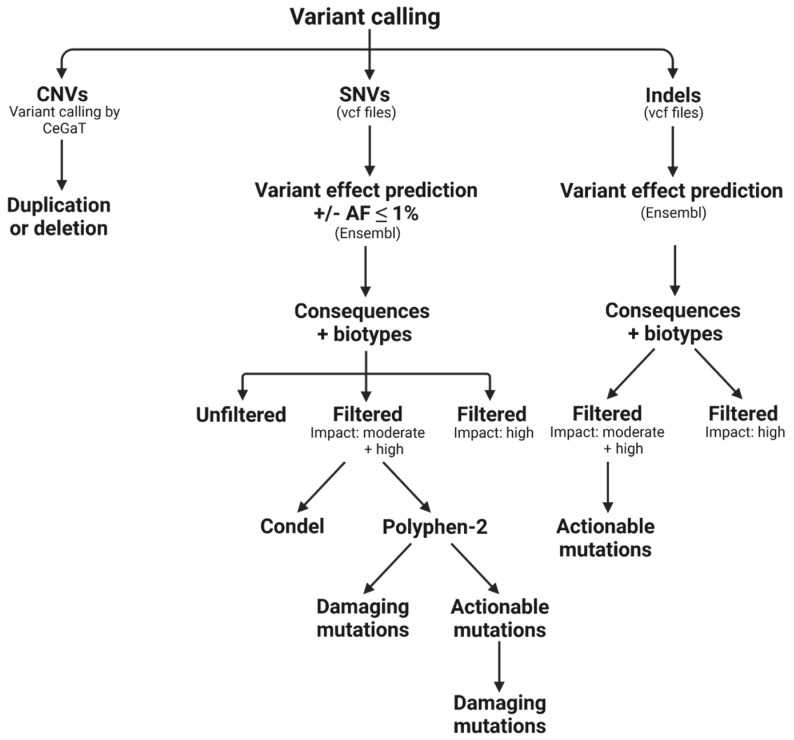
Collected data were processed by variant calling/variant effect prediction (Ensembl) of vcf-files for detected indels and SNVs, and resulting data were analyzed for biotypes, consequences, impact, damaging/severity scores PP-2 and Condel and actionable variants (suggested by COSMIC database). Called CNVs were analyzed for duplications or deletions.

Variant detection and prediction of vcf-files for SNVs and indels (Files S30–S87) was performed using the Ensembl Variant Effect Predictor (VEP) followed by several filtering steps as outlined in Figure 2.

SNV raw variant calls (VEP) were either filtered by allele frequency (AF ≤ 1%) or not filtered and duplicate calls were always removed by a small self-coded software tool (available on request) to quantify and compare the number of SNVs for the indicated conditions. In this process, duplicates were removed according to the terms: “location”, “allele”, “symbol”, “protein position”, “amino acid” and, if present, “PolyPhen-2 (PP-2) score”, whereby from duplicates with PP-2 scores the ones with the lesser damaging PP-2 score were excluded. Subsequent processing steps determined biotypes and consequences, followed by filtering for moderate and high impact (in combination with AF ≤ 1%), which includes variants that might change protein function (moderate) as well as variants that are assumed to have a disruptive impact on the protein (high). Additional filtering also included damaging scores, such as PolyPhen-2 (PP-2) [25], an algorithm that predicts a possible impact of amino acid substitutions on protein structure and function, or Condel, which aggregates an output score for SIFT, PP-2, MAPP, LogR Pfam E-value and MutationAssessor for deleteriousness [26], on top of impact filtering. Actionable variants were selected according to the COSMIC complete actionability database (Download V93), which lists actionable genes and specific variants with putative therapeutic options. Afterward, types of consequences, biotypes and PP-2 scores of remaining variants were automatically determined. Moreover, results were stratified for actionable variants tier 1–4 and tier 1 + 2. In addition, variants with damaging PP-2 scores were more closely investigated; treatment options suggested by COSMIC were listed; and variants were analyzed using different publicly available databases, such as ClinVar, Varsome or OncoKB. Upon VEP prediction for indels, duplicates were removed by filtering according to the terms “location”, “allele” and “feature”. Subsequently, indels were filtered for moderate and high impact or only high impact, and remaining variants were analyzed in relation to types of consequences or biotypes. Actionable variants tier 1–4 as well as tier 1 + 2 in the COSMIC actionability database were identified upon moderate/high prefiltering, respectively.

Furthermore, for CNVs, SNVs (unfiltered + filtered AF ≤ 1%) and indels, the concordance between ev- and tumorDNA and that between cf- and tumorDNA were determined by calculating percent agreement. Data quality was analyzed using the Bland–Altman method [27]. Besides, tumor-, ev- and cfDNA were also examined for clinically relevant BRCA1 and BRCA2 variants.

#### 2.2.2. Bioinformatic Optimization of Filtering Stringency Using AVAtar

Alteration plots for variants detected in tumorDNA and evDNA or cfDNA were generated using the AVAtar software [28]. Visualization was done with “maximal overlap” to compare the variant detection performance of ev-, cf- and a combination of ev- and cfDNA to calculate mean percent coincidence with the tumor upon filtering for SNV variants with a moderate/high impact, moderate/high impact with damaging PP-2 or moderate/high impact with probably damaging PP-2 as a most stringent filtering option.

#### 2.2.3. Medical Art Illustrations

Illustrations were created using BioRender.com (accessed on 4 January 2022).

#### 2.2.4. Statistical Analysis

Statistical analysis was performed using Prism software, version 9.3, for Windows (GraphPad, San Diego, CA) and the MedCalc Statistical Software version 20.015 (MedCalc Software Ltd., Ostend, Belgium; https://www.medcalc.org; accessed on 2 November 2021). Graphs depict mean ± SEM for all conditions. Statistical significance: ns, not significant; * *p* = 0.05–0.01; ** *p* = 0.01–0.001; *** *p* < 0.001; **** *p* < 0.0001.

## 3. Results

### 3.1. Patient Cohort, Study Design and EV Characterization

To evaluate variant detection performance, we investigated a cohort of 10 PDAC patients (Table 1) with matching tumor and liquid biopsy samples for agreement of cf- and evDNA with tumorDNA samples upon panel sequencing. Informed consent was obtained according to institutional ethics approval (67/19). The patient cohort originally comprised 6 male and 4 female patients with advanced metastatic PDAC that were diagnosed at a median age of 56 years. Tumor biopsies were acquired by fine-needle biopsy mainly from the liver metastases, one from the pancreas and one from the pleura. For one patient (patient 2) the DNA isolated from the tumor sample was not sufficient for high-quality sequencing and the respective patient was therefore excluded from comparative analyses. Additional data including therapy of PDAC patients are described in Table S2.

**Figure 3:**
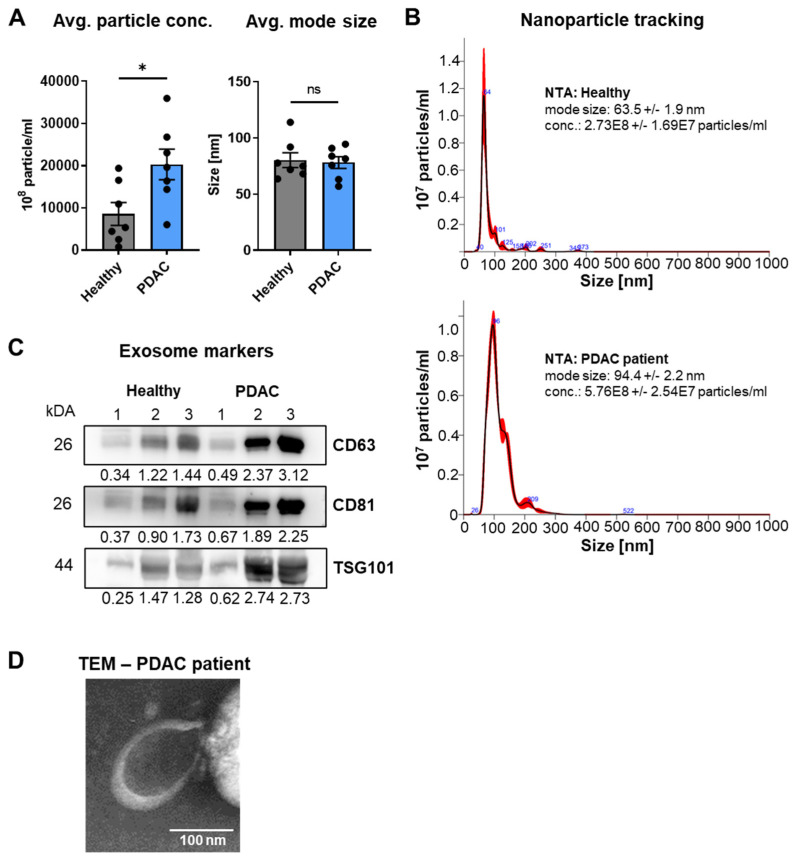
MISEV characterization of EVs after precipitation from plasma of exemplary PDAC patients (*n* = 7) and healthy subjects (*n* = 7). (**A**) Determination of EV particle concentration and mode size. (**B**) Exemplary NTA curves. (**C**) WB analysis of sEV markers for subjects (CD63, CD81, TSG101) and related densitometry of bands. The integrated density of WB bands was measured using ImageJ. To calculate the relative integrated density for each band, values were normalized on the mean of all three healthy subjects. (**D**) Exemplary TEM image of an EV isolated from PDAC patient plasma. Statistical tests: (**A**) Two-tailed unpaired Student *t*-test; * *p* < 0.05; ns: no significant difference.

A valid EV isolation and classification of nanovesicles was verified by subjecting EVs from additional PDAC patients and healthy control subjects (Tables S3 and S4) to a full MISEV guideline analysis (Figure 3) [29] since limited sample volumes prevented MISEV studies for the 10 patients submitted to sequencing of cf- and evDNA. To this end, the mode size of the EVs was determined by nanoparticle tracking analysis (NTA) at 80.20 nm for healthy probands and 85.16 nm for PDAC patients (Figure 3A,B), placing them both firmly in the size range of small extracellular vesicles (sEVs, exosomes).

In line with previous studies that demonstrated increased exosome secretion from tumor cells [30,31], PDAC patients (Table S3) showed a particle concentration significantly increased by 2.4-fold compared to healthy probands (Table S4). The presence of sEVs was further validated by the detection of exosome markers in Western blots (WBs), for three subjects each (Figure 3C). In line with particle tracking data, PDAC patients demonstrated increased levels of exosome surface markers CD63 and CD81 as well as increased levels for the luminal marker TSG101. The WB raw data are provided as Supplemental Figure S37. The presence of sEVs was further validated by transmission electron microscopy (TEM) of uranyl-shaded vesicle preparations with typical cup-shaped features (Figure 3D).

### 3.2. evDNA Contains Significantly More Longer DNA Fragments Compared to cfDNA

**Figure 4:**
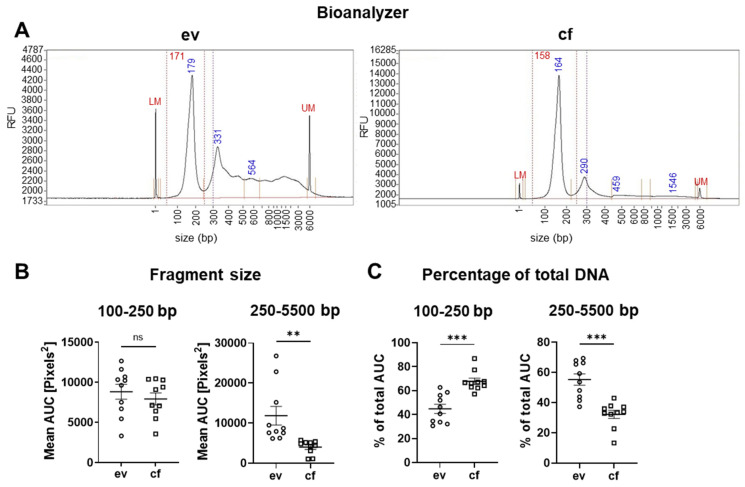
Analysis of DNA quality. (**A**) Exemplary bioanalyzer data of patient 10. (**B**) Statistical analysis of the mean area under the curve (AUC) for ev- and cfDNA in a range of 100 to 5500 bp. (**C**) Percentage amount of DNA fragments of total DNA in a range of 100 to 5500 bp for ev- and cfDNA. Statistical tests: (**B**,**C**) Two-tailed unpaired Student *t*-test; ** *p* < 0.01; *** *p* < 0.001; ns: no significant difference.

Previous reports suggested that analysis of evDNA may be superior to cfDNA, due to increased fragment lengths of up to 10 kb, whereby cfDNA from tumor patients was reported to have a mean length of 120–220 bp [32]. The fragment size of the isolated DNA samples was evaluated using a bioanalyzer device (Figure 4A).

Indeed, there was a significant enrichment of longer DNA fragments in the range of 250 to 5500 bp for evDNA compared to cfDNA, as measured by the mean area under the curve of the bioanalyzer profile (Figure 4B). In addition, we determined the percentage of long fragments (250–5500 bp) in the total AUC (total DNA content). There was a mean value of 55.23% for evDNA compared to 32.17% for cfDNA (Figure 4C). Short fragments with a size of 100–250 bp did not display a significant difference in the mean AUC between ev- and cfDNA. In the total AUC, the percentage of short fragments was 67.83% in cfDNA and 44.77% in evDNA samples. Thus, evDNA was less fragmented and contained a significantly higher percentage of longer DNA fragments, which could improve sequencing performance.

### 3.3. Panel Sequencing and Data Processing

**Figure 5:**
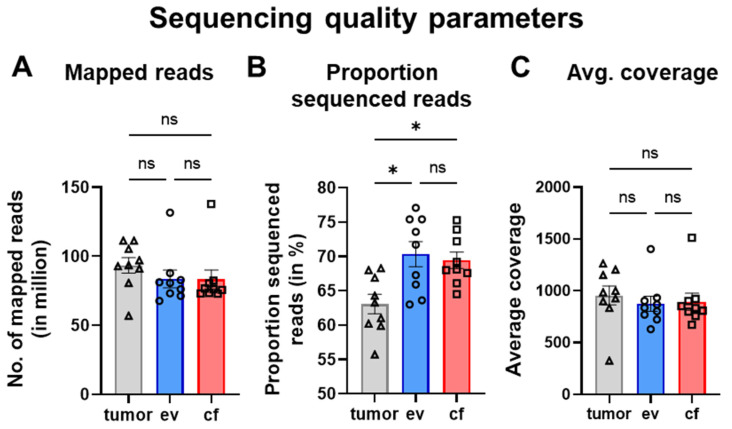
Quality parameters of sequencing data. (**A**) Number of mapped reads (in million). (**B**) Proportion of sequenced reads (in %). (**C**) Average coverage. Statistical tests: (**A**–**C**) Repeated-measures one-way analysis with Tukey multiple comparisons test; * *p* < 0.05; ns: no significant difference.

For commercial sequencing, the TUM01 panel was used. This panel comprises a list of validated variants with potential therapeutic relevance including selected translocations (see Supplemental Table S1). Quality data on sequenced samples are shown in Table 2 and Table 3 and Figure 5 for tumor-, ev- and cfDNA, respectively.

For tumorDNA, the average number of mapped reads was determined at 93,332, whereas the proportion of sequenced reads was on average 63.1% and the mean average coverage (number of reads aligning to known reference bases) was calculated at 955.8 (Figure 5A–C). The coverage per patient can be found in Figure S1A. Similar to the tumor, the average number of mapped reads was around 83.857 for evDNA and 82.368 for cfDNA. The proportion of sequenced reads was somewhat increased for ev- and cfDNA. However, the mean average coverage was not significantly different from the values obtained for tumorDNA. In line, an average Phred quality score (Q30) of 92.12% was determined for tumorDNA, whereas ev- and cfDNA demonstrated an average Phred score of 91.7%, indicating equal accuracy for base calling in tumor and liquid biopsy samples. After initial bioinformatic preprocessing (CeGaT, Tübingen, Germany), reads were mapped to the hg19 reference genome to facilitate variant calling of SNVs and indels. CNVs were called as described in Section 2. Further variant effect prediction for SNVs and indels was performed using the Ensembl VEP interface (variant effect predictor), as outlined in Section 2, with different filtering options delineated in Figure 2 as well as the following results sections.

### 3.4. Variant Calling Results for CNVs, SNVs and Indels

**Figure 6:**
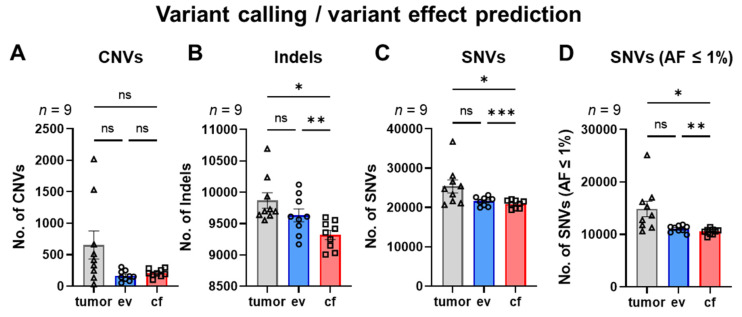
Variant calling/variant effect prediction. (**A**) Number of called CNVs. (**B**) Number of detected indels. (**C**) Number of detected SNVs. (**D**) Number of detected SNVs filtered for low allele frequencies (AF ≤ 1%). Statistical tests: (**A**–**D**) Repeated-measures one-way analysis with Tukey multiple comparisons test; * *p* < 0.05; ** *p* < 0.01; *** *p* < 0.001; ns: no significant difference.

The variant calling for CNVs indicated on average 654 CNVs for tumor samples, whereas for ev- and cfDNA on average only 161 and 203 CNVs were detected, respectively, suggesting that ev- and cfDNA are not very effective in determining CNVs as compared to tumor biopsy material [15,33] (Figure 6A).

Variant calling/variant effect prediction detected an average number of 9868 indels for tumorDNA and 9653 for evDNA, which was statistically not significantly different from the tumor, whereas a statistically significantly lower number of indels was detected for cfDNA (9318) (Figure 6B). SNV prediction identified an average of 25,368 variants in the tumor and 21,606 variants for evDNA, as well as significantly fewer variants for cfDNA (20,912) (Figure 6C). Upon filtering of SNV variants for AF ≤ 1% to increase stringency, the average number of variants in the tumor was reduced to 14,851, whereas filtering of evDNA SNVs generated 11,045 variants and numbers for cfDNA were further significantly reduced to an average of 10,511 (Figure 6D). An overview of the predicted CNVs, indels, SNVs and filtered SNVs (AF ≤ 1%) across all patients detected in tumor-, ev- and cfDNA can be found in Figure S1B–E and Tables S5 and S6. We also investigated SNVs and their chromosomal location in tumor-, ev- and cfDNA samples at the gene level. Respective ideograms for patients 1 to 10, with main mutational hotspots on chromosomes 6 and 19, as well as Venn diagrams indicating overlapping chromosomal positions are listed in Supplemental Figures S2–S11.

In summary, these data suggest that variant prediction from evDNA for indels and SNVs has significant advantages compared to cfDNA. However, CNV calling from ev- and cfDNA in our hands was insufficient for an effective detection of deletions and duplications in PDAC.

### 3.5. Concordance of Variant Calls between evDNA/cfDNA and tumorDNA

To evaluate agreement for all CNVs, SNVs and indel variants called by ev/cfDNAs compared to tumorDNA samples, we further analyzed average percent concordance for all patients. The raw data, indicating the number of matching variants with the corresponding tumorDNA samples, were further subjected to Bland–Altman analysis.

#### 3.5.1. CNVs

In line with the differences in the number of variant calls for CNVs, Bland–Altman plots for matching CNV calls between ev- and tumorDNA as well as between cf- and tumorDNA of the nine patients with complete datasets indicated that most of the data points for both liquid biopsy analytes were outside of the limits of agreement (1.96 s), demonstrating a significant systematic negative bias (Figure S12). The average percent concordance between variants called for tumor- and evDNA as well as for tumor- and cfDNA indicated poor agreement with the tumor of 4.836% for evDNA and 2.876% for cfDNA, respectively. Thus, both liquid biopsy methods under the conditions employed in our study are not suitable to effectively predict CNVs compared to tumor biopsy material. The individual analysis of CNVs for all patients is shown in Figure S13.

#### 3.5.2. SNVs

**Figure 7:**
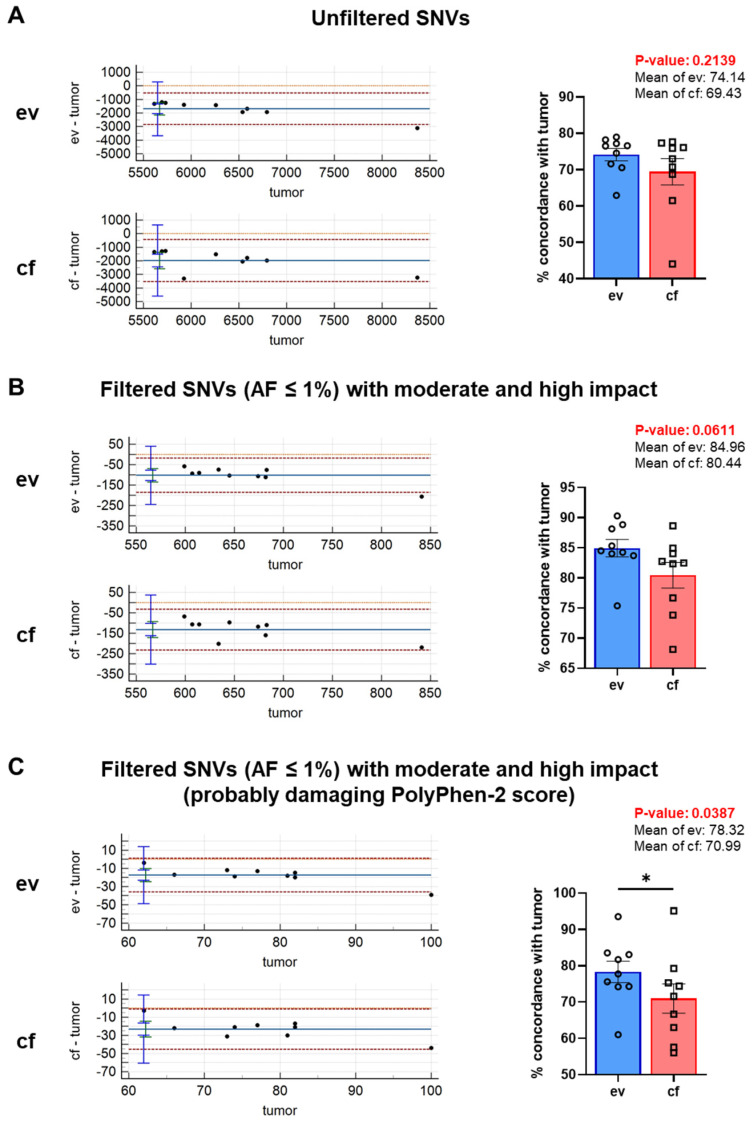
Stringent filtering of variants was applied to increase specificity for tumor-associated variants. Bland–Altman plots and concordance (%) of variants between tumor- and evDNA as well as between tumor- and cfDNA for (**A**) unfiltered SNVs, (**B**) filtered SNVs (AF ≤ 1%) with moderate and high impact and (**C**) filtered SNVs (AF ≤ 1%) with moderate and high impact and a probably damaging PP-2 score. Statistical tests: (**A**–**C**) Two-tailed paired Student *t*-test; * *p* < 0.05.

For SNVs, on the other hand, the Bland–Altman plots indicated that the variances between ev- or cf- and tumorDNA were much smaller. For the evDNA plot, one data point was outside the limits of agreement; for the cfDNA analysis, all data points were within the agreement limits. Nevertheless, the agreement span for the comparison of ev- and tumorDNA was much tighter and the negative systematic bias was smaller as compared to cfDNA, indicating improved data quality (Figure 7A). In contrast to CNVs, SNVs were readily predictable with good consistency with tumorDNA. Unfiltered SNVs showed an average match of about 74.14% for ev- and tumorDNA, whereas the concordance for cfDNA with the tumor was 69.43%.

### 3.6. Stringent Processing of SNV Calls for Tumor-Relevant Variants Substantially Reduces Variant Numbers and Improves Data Quality and Concordance with tumorDNA Samples

To further improve data quality and reduce systematic negative bias in liquid biopsy SNV calling, additional stringent filtering was applied for low allele frequencies (AF ≤ 1%) and moderate/high impact scores (Figure 7B). Bland–Altman plots again indicated tighter limits of agreement and reduced systematic bias for the comparison of evDNA with tumorDNA. One data point was still outside the limits of agreement. Upon filtering, the average percent concordance with the tumor was further increased to 84.96% for evDNA and 80.44% for cfDNA. By increasing stringent filtering utilizing the PP-2 probably damaging classification, we were able to minimize the negative systematic bias and improve data quality (Figure 7C). Percent concordance was now 78.32% for evDNA and 70.99% for cfDNA. Even though data quality and in particular negative systematic bias were strongly normalized by rigorous PP-2 (probably damaging) filtering, percent concordance could not be improved in this context and reached a plateau using the moderate and high impact score. Yet, additional PP-2 filtering revealed significant differences for the concordance of evDNA with tumorDNA as compared to cf- and tumorDNA and further reduced the number of variants that require evaluation during additional downstream processing, e.g., for actionable variants.

Next, we assessed whether filtering using an alternative damaging score would be able to further enhance agreement with the tumor. To this end, we utilized the Condel score, which aggregates five databases and was described to be superior to PP-2 [26]. Interestingly, additional filtering of AF ≤ 1% and moderate- and high-impact SNVs with a deleterious Condel score did not improve but rather decreased percent concordance with the tumor to 70.85 and 67.64% for ev- and cfDNA, respectively (Figure S14), indicating that if additional stringency processing is required, filtering with the PP-2 score is superior to Condel during ev- and cfDNA analysis of SNVs. In line with PP-2 filtering, data quality as determined by Bland–Altman analysis indicated tighter limits of agreement for evDNA, and the negative systematic bias was also slightly reduced in comparison to cfDNA.

We have shown that the concordance of SNVs detected in ev- and cfDNA with tumor biopsy samples is strongly dependent on stringent filtering for tumor-relevant variants. Moreover, detection performance significantly improved upon rigorous filtering in the case of evDNA. Next, we systematically explored how the respective single analytes or a combination of both liquid biopsies would fare against tumorDNA sequencing by generating alteration plots using AVAtar, after employing different filters [28]. To this end, for each patient, percent coincidence of alterations upon filtering was detected with the objective “maximal overlap” to compare detection performance, whereby the *n*-number indicated the number of different variants detected in the tumor over all patients. A representative alteration plot depicting AVAtar results for ev-, cf- and a combination of ev- and cfDNA is shown in Figure S15A,B. In line with our previous findings (Figure 7), on average, evDNA detection performance was improved over cfDNA. In addition, the number of detectable variants was reduced from *n* = 1024 (impact moderate/high) to *n* = 144 (impact moderate/high + PP-2: damaging) and further to *n* = 84 for the highest stringency (impact moderate/high + PP-2: probably damaging). At the same time, mean percent coincidence for all patients was detected at 69% for evDNA, 67% for cfDNA and 84% for a combination of ev- and cfDNA (impact moderate/high). Rigorous filtering and consequent strong reduction of n-numbers again somewhat reduced concordance to 65% for evDNA, 62% for cfDNA, and 80% for ev- and cfDNA (impact moderate/high + PP2: damaging). Interestingly, the most stringent filtering (PP-2: probably damaging) did not reduce concordance for all conditions any further. Systematic optimization using the AVAtar tool therefore indicated that the number of variants for subsequent downstream analysis can be reduced by a factor of 12 by employing a PP-2 probably damaging score, without drastically sacrificing detection efficacy. Furthermore, our data show that the combined analysis of ev- and cfDNA has a clear advantage (80% as compared to 65% or 62%, respectively) and could be considered as a new standard when comparing detection performance for SNVs with respect to tumor biopsies. Of note, while comparing variants using AVAtar, we have identified a number of variants for the following genes across all samples and all patients: lysine N-methyltransferase 2C (KMT2C, seven variants); mitogen-activated protein kinase kinase 3 (MAP2K3, five variants); fms-related receptor tyrosine kinase 3 (FLT3, one variant); serine protease 1 (PRSS1, one variant); PARP4, one variant; gamma-glutamyltransferase 1 (GGT1, one variant), nuclear receptor corepressor 1 (NCOR1, one variant); and ERCC excision repair 5, endonuclease (ERCC5, one variant). The respective variants are listed in Table S7 together with their CinVar, Varsome and OncoKB scoring. Although none of the variants was identified as pathogenic, many remain of uncertain significance. Furthermore, some of the genes were described as important regulators during PDAC development and progression; e.g., KMT2C/myeloid/lymphoid or mixed-lineage leukemia protein 3 (MLL3) is a histone methyltransferase [34] and chromatin modifier with a large impact on the expression of chromatin-regulating genes and genes involved in cell proliferation [35], suggesting that MLL defects likely cause global epigenetic alterations that support tumor development. Concerning MAP2K3, the constitutive activation of MAPK signaling was described in pancreatic cancer by [36]. FLT3 may be a potential biomarker for individualized pancreatic cancer prognosis [37]. Germline mutations in PRSS1 were associated with familial forms of chronic pancreatitis and extreme risk of PDAC [38,39]. PARPs have been implicated in the pathogenesis of pancreatitis as well as pancreatic cancer, and certain germline mutations were identified in patients with thyroid and breast cancers [40,41]. GGT1 has a suggested function in pancreatic carcinogenesis [42], whereas NCOR1 is part of a corepressor complex with histone deacetylase 3 (HDAC3) and may act as an oncogene in thyroid cancer [43,44], while ERCC5 polymorphisms were reported in breast cancer [45,46].

In summary, no known pathogenic germline or somatic variants were identified for the respective genes; nevertheless, their presence in all patients may warrant further functional analysis, as gene-level information hints at interesting connections to pancreatic cancer carcinogenesis and progression.

#### Indels

**Figure 8:**
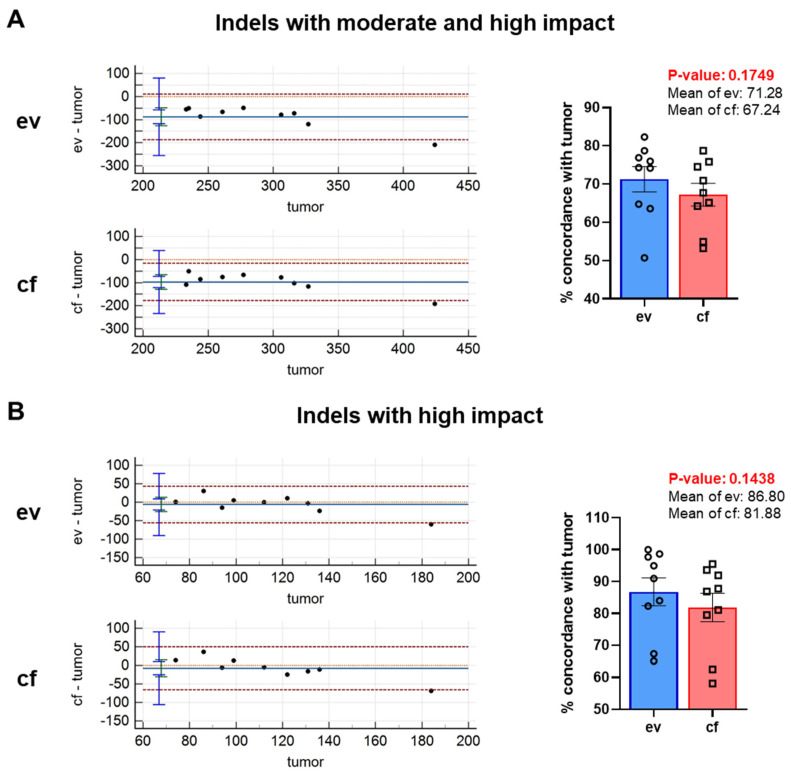
Stringent filtering of variants was applied to increase specificity for tumor-associated variants. Bland–Altman plots and concordance (%) of variants between tumor- and evDNA as well as between tumor- and cfDNA for (**A**) indels with moderate and high impact and (**B**) indels with high impact. Statistical tests: (**A**,**B**) Two-tailed paired Student *t*-test.

We also determined the agreement of evDNA and cfDNA with the tumor with respect to indels (Figure 8). To this end, we immediately employed filtering using moderate and high settings since the respective filtering conditions were proven to be effective in the SNV analysis. Bland–Altman plots indicated that all but one data point were set within the limits of agreement for both ev- and cfDNA and a similar significant systematic bias was detectable (Figure 8A).

Percent concordance for variant calls of evDNA with tumorDNA was around 71.28%, and that of cfDNA with tumorDNA was 67.24%. The difference between ev- and cfDNA was not significant. In line with SNVs, increased stringency of filtering to high-impact indels substantially improved data quality and further reduced the systematic bias for both ev- and cfDNA (Figure 8B). Again, one data point was outside of the limits of agreement for both comparisons. Percent concordance was improved to 86.80% for evDNA and 81.88% for cfDNA. However, there was only a numerical but no statistically significant difference between ev- and cfDNA. Whether concordance will become significant in larger cohorts remains to be tested in subsequent studies.

In summary, agreement analysis for CNVs, SNVs and indels indicated that in our patient cohort a global determination of CNVs by ev- and cfDNA sequencing of PDAC liquid biopsies is not sensible. The evaluation of SNVs demonstrated a good concordance with tumors indicating valid results upon sequencing of evDNA with subsequent stringent processing, which improved data quality and markedly reduced systematic negative bias. It is important to note that filtering algorithms for damaging scores severely impact concordance analysis, as the PP-2 score was superior to Condel in improving filtering stringency. The analysis of indels also demonstrated high and similar concordance with the tumor for both ev- and cfDNA. Data quality was again improved upon stringent filtering for high-impact variants. These results suggest that evDNA sequencing may significantly improve the detection of SNVs and indels in larger patient cohorts.

### 3.7. Consequences and Biotypes of Detected Variants

We also wanted to understand how the different variants would impact the structure and function of a gene product. To this end, we analyzed consequence and biotype predictions using the VEP analysis tool (consequences: www.ensembl.org/info/genome/variation/prediction/predicted_data.html (accessed on 15 December 2021); biotypes: https://m.ensembl.org/info/genome/genebuild/biotypes.html (accessed on 15 December 2021)) for SNVs and indels in the respective tumor-, ev- and cfDNA samples. The analysis of consequences for SNVs in individual patients with and without filtering SNVs (AF ≤ 1%, with moderate and high impact) is shown in Figures S16–S25.

**Figure 9:**
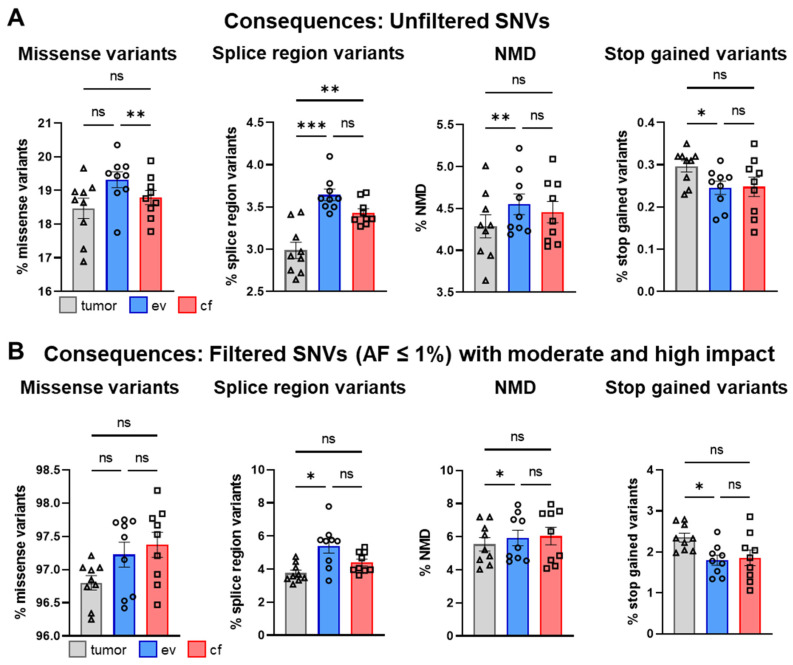
Analysis of consequences for SNVs. (**A**) Mean percentage of most prevalent consequences in tumor-, ev- and cfDNA of total consequences across all patients for unfiltered SNVs and (**B**) filtered SNVs (AF ≤ 1%) with moderate and high impact. Statistical tests: (**A**,**B**) Repeated-measures one-way ANOVA with Tukey multiple comparisons test; * *p* < 0.05; ** *p* < 0.01; *** *p* < 0.001; ns: no significant difference.

We also compared the most abundant variants before (Figure 9A) and after filtering (Figure 9B) across all patients. The most prominent consequences for tumor-, ev- and cfDNA after filtering were missense- and nonsense-mediated decay (NMD) transcript, splice region and stop-gained variants (Figure 9B). Here, the percentage of missense variants markedly increased from around 18% (tumor-, ev-, cfDNA) to more than 97%, while splice region and NMD variants were not drastically changed. However, stop-gained variants increased from 0.26% to more than 2% across all samples. There were also some significant differences detectable between tumor-, ev- and cfDNA before filtering, which were mostly normalized by the filtering process due to improved data quality as shown in Figure 7.

**Figure 10:**
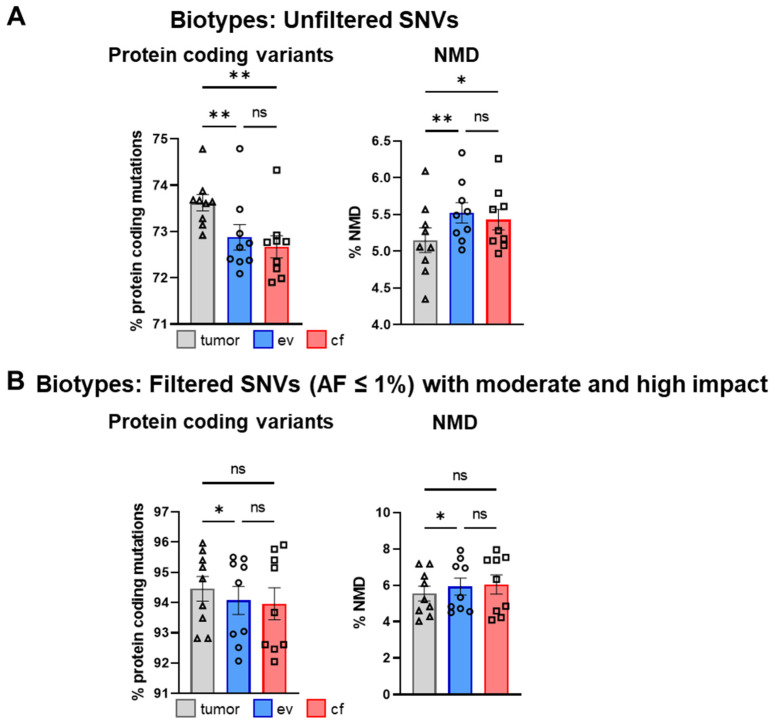
Analysis of biotypes for SNVs. (**A**) Mean percentage amount of most prevalent biotypes in tumor-, ev- and cfDNA of total biotypes across all patients for unfiltered SNVs and (**B**) filtered SNVs (AF ≤ 1%) with moderate and high impact. Statistical tests: (**A**,**B**) Repeated-measures one-way ANOVA with Tukey multiple comparisons test; * *p* < 0.05; ** *p* < 0.01; ns: no significant difference.

Furthermore, we determined the most prevalent biotypes of the respective variants according to the VEP biotype legend, again before (Figure 10A) and after filtering (Figure 10B).

After filtering, protein-coding followed by NMD were the most predominant variants (Figure 10B). The percentage of protein-coding variants was enriched from around 73.06% to over 94.16% after filtering, and the percentage of NMD variants was slightly increased (Figure 10B). Again, significant differences between tumor-, ev- and cfDNA were normalized by filtering as shown for the consequence analysis. The analysis of biotypes for individual patients is shown in Supplemental Figures S16–S25.

**Figure 11:**
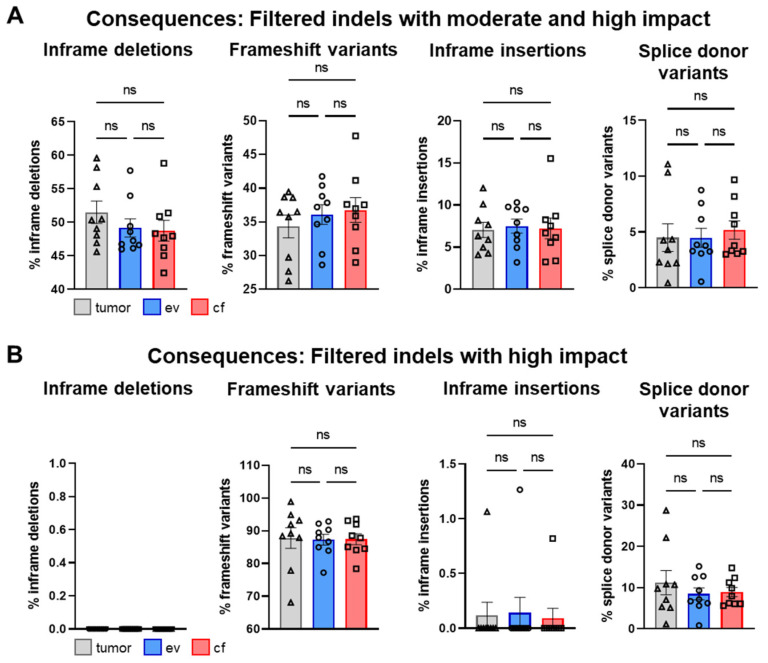
Analysis of consequences for indels. (**A**) Mean percentage amount of most prevalent consequences in tumor-, ev- and cfDNA of total consequences across all patients for indels with moderate and high impact and (**B**) indels with high impact. Statistical tests: (**A**,**B**) Repeated-measures one-way ANOVA with Tukey multiple comparisons test; ns: no significant difference.

Concerning indels, the most predominant consequences after moderate and high impact filtering were inframe deletions with a percentage of about 49.80%, followed by frameshift variants at about 35.74%, inframe insertions with 7.267% and splice donor variants with ~5% (Figure 11A).

**Figure 12:**
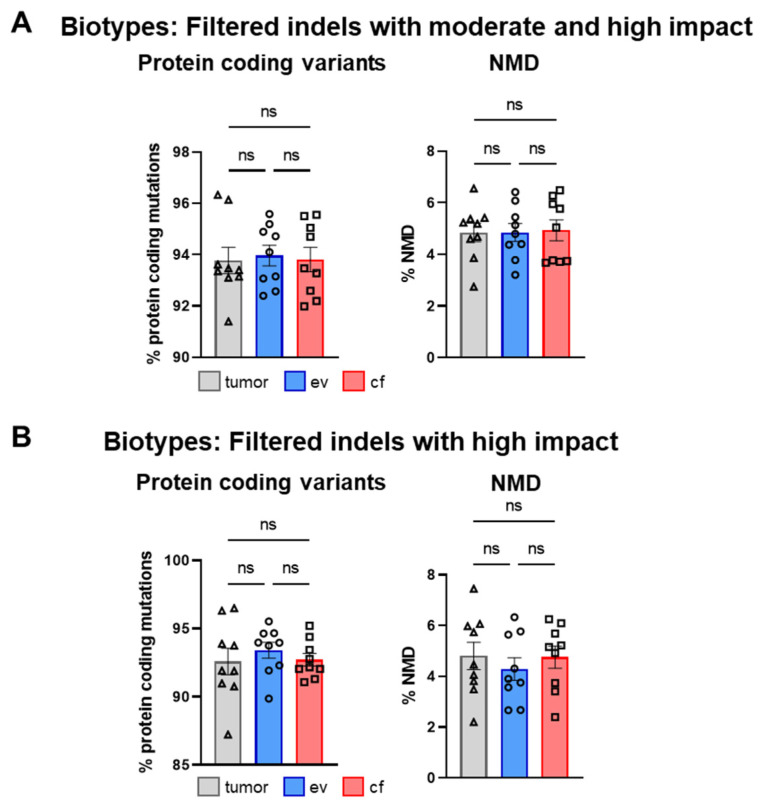
Analysis of biotypes for indels. (**A**) Mean percentage amount of most prevalent biotypes in tumor-, ev- and cfDNA of total biotypes across all patients for indels with moderate and high impact and (**B**) indels with high impact. Statistical tests: (**A**,**B**) Repeated-measures one-way ANOVA with Tukey multiple comparisons test; ns: no significant difference.

In line with the SNV analysis, after filtering, no significant differences were detected for tumor-, ev- and cfDNA (Figure 11B). The individual analysis of consequences for all patients is shown in Supplemental Figures S26–S35. Interestingly, upon high impact filtering, the composition of consequences drastically changed, and inframe deletions as well as inframe insertions were almost completely lost, whereas frameshift variants increased to 87.5%, suggesting that high impact filtering of indels has to be considered with caution, since putative relevant deletions may be removed (Figure 11B). Concerning biotypes, the most predominant variants upon moderate/high or high impact filtering were protein-coding variants with > 90% (Figure 12A,B). The individual analysis of biotypes for all patients is shown in Supplemental Figures S26–S35.

### 3.8. Comparison of BRCA1/2 Variant Prediction between ev-, cf- and tumorDNA

**Figure 13:**
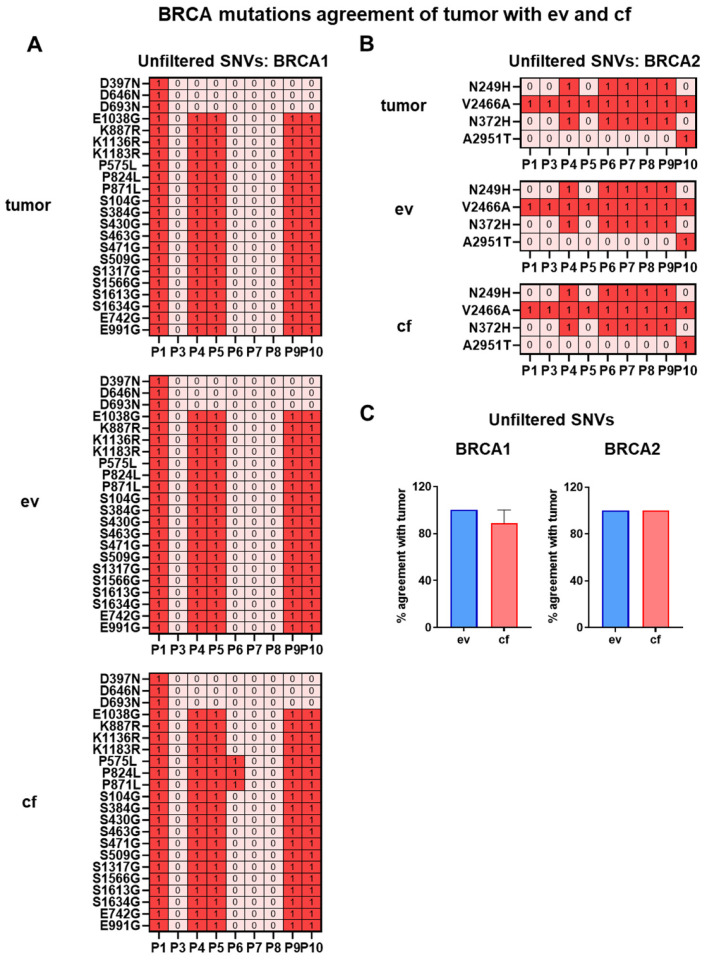
Heat maps of BRCA variant concordance between tumor-, ev- and cfDNA. (**A**) BRCA1 and (**B**) BRCA2 variants of unfiltered SNVs. (**C**) Percentage concordance of BRCA1 and BRCA2 variants between tumor-, ev- and cfDNA. Statistical tests: (**C**) Repeated-measures one-way analysis with Tukey multiple comparisons test.

Besides the analysis of consequences and biotypes, we were interested in evaluating whether clinically relevant and therapeutically meaningful gene variants are properly reflected by ev- and cfDNA analysis in comparison to tumor biopsy samples. To this end, the incidence of germline pathogenic BRCA variants in pancreatic cancer was described with a prevalence ranging from 0.3–2.3% for BRCA1 and 0.7–5.7% for BRCA2 [47]. BRCA variants that impair protein function are known to sensitize tumors to platinum analogs and inhibition with the poly(adenosine diphosphate ribose) polymerase (PARP) inhibitor olaparib [48]. In our patient cohort, we identified 22 BRCA1 variants (Figure 13A) in unfiltered SNVs, which however were not listed as damaging germline variants in the ClinVar database.

We therefore went on to compare BRCA1 and -2 variants between the different analytes to evaluate variant prediction performance for clinically relevant genes. Interestingly, two BRCA1 variants, E742G and E991G, have been classified as possibly damaging by PP-2. A complete classification by ClinVar, Varsome and OncoKB databases of all detected BRCA variants in our patient cohort is shown in Table S8. Average percentage concordance for evDNA with the tumor was determined to be 100%, whereas for cfDNA concordance was lower with 88.89%, suggesting that evDNA may have a slight advantage for the detection of BRCA1 variants in patients (Figure 13C). For BRCA2, we have identified four variants, which however were also not listed as pathogenic germline mutations in ClinVar. As shown in Figure 13B, all variants were detected in both ev- and cfDNA, compared to tumorDNA. A full list of all BRCA1 and -2 variants in unfiltered SNVs of tumor-, ev- and cfDNA in all patients is shown in Tables S9 and S10. For indels, no BRCA variants were detected, whereas BRCA1 CNVs detected in tumor-, ev- and cfDNA are listed in Table S11, which however did not include homozygous deletions but did include one heterozygous deletion with unknown impact on the function of the gene product. Although we have determined that CNV analysis utilizing ev- and cfDNA is not favorable, we have actually detected the same BRCA1 CNVs in tumor- and evDNA, including the heterozygous deletion, in four out of nine patients and none in cfDNA. This suggests that for specific variants, CNV detection by evDNA might be possible in future applications, upon optimization of EV isolation, sequencing and bioinformatic processing steps. In conclusion, although we did not detect relevant germline variants, the BRCA1 mutational analysis indicates that there could be an advantage for the detection of specific, clinically relevant SNVs in unfiltered evDNA.

### 3.9. Agreement of Actionable Variant Prediction by ev- and cfDNA with Tumor Samples

Next, we went on to investigate how efficiently clinically relevant actionable genes and specific variants are reflected by ev- and cfDNA with respect to tumorDNA samples. We have therefore compared a list of moderate/high filtered SNVs (AF ≤ 1%) in combination with a stringent PP-2 damaging score as well as moderate/high filtered indels against the COSMIC actionability database (Download version 93). This database includes mainly gene-level actionable information and some specific variants sorted into the four main groups, i.e., tier 1–4, whereby approved marketed drugs with demonstrated efficacy at the gene/mutation were classified as tier 1. Tier 2 is described as phase 2/3 clinical trial results, which meet the primary outcome measures of the clinical trial, whereas tier 3 is drugs in ongoing trials and tier 4 is case studies. Upon stringent filtering, as described above, we have reduced the number of SNV variants from on average 22,623 SNVs in unfiltered VEP predictions to around 11 variants for PP-2-sorted tier 1–4 actionable genes. A graph displaying the number of all actionable PP-2-classified variants stratified for unknown, benign and possibly and probably damaging scores, as well as only the damaging PP-2 variants, is shown in Figure S36. When applying an additional tier 1 + 2 filter, numbers were further reduced to four variants per patient (Figure S36). The respective tables for tumor-, ev- and cfDNA indicating the number of variants are presented as Tables S12–S14.

**Figure 14:**
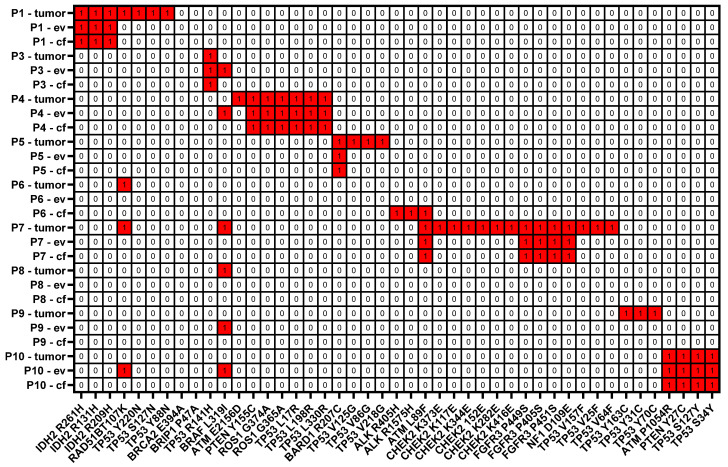
Analysis of tier 1 + 2 actionable variants. Tier 1 + 2 actionable variants detected in filtered SNVs (AF ≤ 1%) with moderate/high impact and damaging PP-2 score of tumor-, ev- and cfDNA per patient.

Since all detected actionable variants were selected by filtering on the gene level using the COSMIC database (Tables S15–S17), we went on to compare the specific variants across additional databases, including ClinVar, Varsome and OncoKB. The respective results are shown in Table 4, which lists specific variants; severity scores from ClinVar, Varsome and OncoKB; and levels of evidence for proposed treatment options, if available. When comparing the detection performance for actionable variants depicted in Figure 14 across all analytes, our data show that tumorDNA consistently identified more variants in actionability genes compared to ev- and cfDNA. Concerning concordance of the detected variants for liquid biopsy analytes with the tumor, we show that both ev- and cfDNA demonstrated similar concordance of ~43%, as in six out of nine patients, matching tumor variants were identified by the liquid biopsy analytes. Interestingly, evDNA detected five additional variants (BRAF L319I, RAD51B T107K) in patients 3, 4, 9 and 10 that were not present in the respective tumor. cfDNA, on the other hand, detected three additional variants in patient 6 (ATM L98F, ALK R405H, ALK R1575H). Interestingly, these variants were also not found in tumor- and evDNA. Thus, these data show that, in particular, evDNA was able to detect additional variants that are not reflected by tumorDNA sequencing. However, whether these variants are relevant and display increased tumor heterogeneity that is not covered by localized biopsy stances cannot be determined with the available data.

Upon comparison of the variants identified in the COSMIC actionable gene list with additional databases, three out of nine patients (4, 7 and 10) displayed the following actionable variants with treatment options in tumorDNA: patient 4: PTEN Y155C (AZD8186, GSK2636771), patient 7: CHEK2 K373E (olaparib) and patient 10: PTEN Y27C (AZD8186, GSK2636771), which were also found in the case of patients 4 and 10 in both ev- and cfDNA. For indels, in tumorDNA in all patients, moderate- and high-impact variants in actionable genes were detected, which include frameshift variants in CHEK1 and TP53 as well as inframe deletions in ABL1 and FGFR1. Overall, more indels were detected in actionable genes by tumorDNA, and again mean percent coincidence for ev- as well as for cfDNA was calculated at ~43%. Indeed, matching actionable variants were found in four out of nine patients for evDNA (CHECK1, TP53, ABL1) and in five out of nine patients for cfDNA (CHEK1, ABL1) as shown in Tables S18–S20. For evDNA, additional indels in actionable genes were detected for patients 4, 5 and 6, whereas cfDNA found additional indels in patients 6, 8, 9 and 10 (Tables S18–S20). We have also cross-referenced all indels of the respective actionable genes with the ClinVar, Varsome and OncoKB databases, but no previously described variants were identified. However, as indels with high-impact frameshift variants were detected in the respective genes, an altered protein functionality is very likely. In summary, concerning actionability analysis, our data show that tumorDNA consistently identified more variants in actionability genes compared to ev- or cfDNA, which demonstrated a similar coincidence of 43%, respectively, with the tumor. Although evDNA displayed improved detection concordance for SNVs and indels with the tumor upon stringent filtering, no relevant differences were detected concerning actionable variants with the proposed treatment options. However, this might be explained by the small cohort size and needs further investigations in larger collectives.

## 4. Discussion

In our proof-of-concept study, we have performed a comparative analysis of DNA variants detected in cf-, ev- and tumorDNA from PDAC patients upon panel sequencing using a large diagnostic tumor gene panel (TUM01, CeGaT, Figure 1, Table S1). We have analyzed a cohort of 10 PDAC patients with advanced metastatic tumors that have been presented at the clinical molecular tumor board to assess actionable DNA variants by sequencing (Table 1 and Table S2). Bioanalyzer DNA characterization indicated significantly longer fragment sizes for evDNA (up to 5500 bp) (Figure 4), which was described to improve sequencing quality due to chromatin superstructures that favor amplification during NGS [17,18]. Moreover, we show improved detection performance for evDNA in calling of SNVs and indels upon stringent bioinformatic processing for high-impact tumor-relevant variants (Figure 7 and Figure 8). A systematic bioinformatic optimization of filtering steps further indicated that rigorous filtering using impact (moderate/high) and PP-2 (damaging) scores drastically reduced the number of SNV variants that need to be considered for further downstream analysis of actionable variants, without a major sacrifice of detection coincidence with the tumor samples. Moreover, the combined detection performance of ev- and cfDNA was clearly superior to either single analyte. In line with previous observations in other tumor entities, calling of CNVs was challenging and uninformative for both ev- and cfDNA (Figure 6 and Figure S12). We have also determined concordance for actionable variants from the respective biopsy samples (Figure 14, Tables S12–S14). Yet, upon filtering for actionable genes, concordance of actionable variants that were also found in the tumorDNA was no longer significantly improved in evDNA as compared to cfDNA (Figure 14, Table 4). This may be explained by stringent filtering for actionable, damaging variants, as well as the small cohort size employed in our study. Although we also identified additional variants found only in ev- and cfDNA, which may represent tumor heterogeneity, it is currently unclear whether the detected variants are indeed tumor-derived or represent somatic variants in other cells. In summary, concordance analysis suggests that for SNVs and potentially indels, evDNA could improve detection efficacy and a combination of ev- and cfDNA is superior (Figure 7 and Figure 8). However, further optimization, standardization and larger cohort sizes will be required to acquire a fully informative statement on improved detection of actionable variants.

The results of our study agree with previous findings that liquid biopsies and analysis of cf- and/or evDNA during mutational profiling of tumors may in defined instances be able to replace invasive tumor fine-needle biopsies, e.g., for longitudinal characterization of tumors through treatment cycles. In line, fine-needle biopsies were described to be a significant burden for patients [9,49] and are often hard to obtain; in some cases, tumors are inaccessible and biopsy stances only represent a snapshot of the tumor, which does not fully represent clonal heterogeneity or metastases [49]. In contrast, liquid biopsies are easy to obtain with low burden for patients and cause lower costs for the healthcare system [11,50]. Nevertheless, recent advances in patient organoid technology [51] have created additional utility for biopsy material, allowing the examination of personalized responses of the respective patient-derived organoids to chemotherapeutic agents and small-molecule inhibitor targets identified by molecular profiling. To this end, our data further suggest that for the initial diagnosis of PDAC and the molecular characterization of tumor DNA variants, fine-needle biopsies can so far not be replaced by liquid analytes with a similar high detection efficacy, yet actionable variant detection may be supported by a combination of easy-to-obtain cf- and evDNA since they are thought to more closely represent overall tumor heterogeneity [52,53]. To this end, additional variants were also observed in ev- and cfDNA in our study (Figure 14). However, there are also some caveats concerning cf- and evDNA profiling; e.g., cfDNA comprises circulating free tumor-derived (ct)DNA and non-tumor-derived DNA, released from other somatic cells in the body, which can also be mutated [54] (see Figure 13, BRCA1 variants). Moreover, EVs are secreted by almost every cell type, impairing tumor-specific DNA analysis [55]. For evDNA, some of these disadvantages may be compensated, e.g., by additional immune purification of tumor-specific sEVs [56], which are also the main fraction of EVs in our samples (Figure 3). Interestingly, successful immune enrichment of tumor sEVs has been shown as a proof-of-concept study in PDAC patients for the analysis of KRAS mutations by ddPCR; however, further optimization is still required prior to clinical implementation [16]. Tumor cells were also described to release higher sEV concentrations [57], which is in line with Figure 3, where particle concentrations for PDAC patients were significantly elevated by 2.4-fold and sEV marker expression was increased as compared to healthy subjects. Thus, this may be of advantage for tumor evDNA profiling, since evDNA was also described to contain longer DNA fragments up to 10 kb [18] (Figure 4), whereas ctDNA fragments (<100 bp) are even significantly shorter than cfDNA [19,20]. Nevertheless, due to easy isolation and commercially available purification kits, cfDNA is currently still widely used as an important liquid biopsy analyte, but this is slowly changing as different advantages of evDNA are validated in more and more studies [58,59]. Of note, well-established commercially available cfDNA purification kits may at least partially copurify evDNA, which is not separated from plasma and serum samples during preparation [60]. This might also explain the detection of the small fraction of longer-sized DNA fragments in cfDNA (Figure 4). Moreover, most of the studies performed so far have utilized highly sensitive ddPCR to compare cf- and evDNA concordance for specific variants [16], while broad NGS approaches are rare [60]. We have therefore aimed to investigate the global concordance of cf- and evDNA analytes with tumorDNA upon sequencing with a larger diagnostic tumor panel. Our study has also limitations, such as the larger gene panel, which did not allow for sequencing with extremely high coverage due to high sequencing expenses. Nevertheless, we were able to successfully resolve the majority of SNVs and indels also covered by tumorDNA, in particular for evDNA samples (Figure 7 and Figure 8). We want to further caution that since comparative Bland–Altman graphs during concordance analysis (Figure 7 and Figure 8) are based on nine patients, the data suffer from higher variations due to small n-numbers.

In our study, we compared concordance between DNA variants detected by liquid biopsy analytes and the tumorDNA for CNVs, indels and SNVs. CNV calling from ev- and cfDNA was challenging and uninformative when compared to the tumorDNA samples, which detected ~3–4 times higher CNV numbers (Figure 6 and Figure S1B). This is in line with previous reports, indicating CNV detection with variable efficacies, depending on tumor entities and in particular the fraction of ctDNA in samples. A study by Chae et al. showed a concordance for CNV detection of only 3.5% by sequencing with a Guardant360 and FoundationOne panel in a cohort of 45 breast cancer patients [33]. These data align with the results from our study for the TUM01 panel (CeGaT), which detected a concordance of 2.876% for cfDNA and 4.836% for evDNA with tumor material (Figure S12), indicating increased concordance of CNV prediction by evDNA (Figure S12). However, higher concordance values were reported in other tumor entities. In a cohort of 45 prostate cancer patients, a concordance of 48.9% between cfDNA and tumor tissue was detected [61]. This may be explained by study requirements for higher ctDNA fractions of >35% in the samples. These data indicate that the parameters ctDNA or tumor EV fraction must be considered when planning clinical studies or implementing liquid biopsy analysis in the clinical routine. To this end, the tumor-derived cfDNA fraction may be quantified or enriched by focusing on smaller fragmented DNA sizes [62]. Similar considerations apply to tumor EVs. Here, detection or enrichment may be possible by tumor-specific markers such as glypican-1 [30] or using immune-enrichment [56].

Nevertheless, this aspect requires further investigation with studies that are specifically tuned towards tumor-derived cf- and evDNA enrichment and CNV detection, including improved bioinformatic processing. For SNVs, higher concordance values were obtained for evDNA after stringent filtering for tumor-relevant, high-impact, damaging variants, using impact (moderate/high) as well as PP-2 (damaging) scores (Figure 7C and Figure S15A), which drastically reduced the number of variants for downstream applications. Filtering even improved data quality, as analyzed by the Bland–Altman method, by reducing systematic bias. Hence, our data indicate that for accurate SNV detection, stringent filtering of tumor-relevant variants is essential. However, it must be noted that an optimal balance has to be achieved between stringent filtering and a sensitive detection of SNVs to avoid the exclusion of important variants. The type of filtering strategy chosen to improve data quality and concordance also seems to be of major importance. Here, we demonstrate that filtering with a PP-2 damaging score was superior to a stringent, aggregated prediction by Condel, which further impaired overall percent concordance (Figure S14). Thus, filtering optimization suggests that the application of an additional PP-2 damaging score may be the optimal trade-off strategy to improve data quality and concordance with the tumor, while at the same time drastically reducing variant numbers for downstream analysis. For indels, improved concordance upon rigorous filtering was also shown similar to SNVs (Figure 8B), yet concordance here was only numerically increased for evDNA and thus requires further follow-up investigations in larger cohort sizes.

To improve the clinical relevance of our study, we have also analyzed SNVs with moderate and high impact, as well as a damaging PP-2 score, for clinically meaningful variants such as BRCA1, which sensitizes PDAC tumors to platinum analogs and inhibition with the PARP inhibitor olaparib [48] upon loss of protein function. Although we did not detect known germline variants, likely due to low prevalence, we were able to identify 22 BRCA1 variants (Figure 13), which were more readily detected in evDNA across patients than in cfDNA (concordance with tumor for evDNA: 100% and cfDNA: 88.89%) (Figure 13C). Thus, also for specific, clinically relevant genes, improved detection of variants by evDNA with respect to cfDNA can be demonstrated, yet this does not always apply to all important variants, as shown for BRCA2 with 100% concordance for both liquid biopsy analytes. Upon performing a global concordance analysis for the detection of variants in tier 1 + 2 actionability genes, no significant differences for the detection of actionable SNV variants from cf- and evDNA were recorded (Table 4). This may be explained by rigorous data processing using moderate/high impact, filtering with PP-2 scores, as well as the selection of actionable tier 1 + 2 genes, which resulted in the identification of on average three positive hits for ev- and cfDNA. Thus, in follow-up studies, larger cohort sizes may be needed to fully elucidate any potential differences in the detection of actionable variants. In summary, we propose that a combination of ev- and cfDNA should be considered as a novel gold standard for liquid biopsy-based detection of DNA variants and may help to support mutational profiling of tumor samples concerning tumor heterogeneity or longitudinal analysis during treatment.

## 5. Conclusions

In conclusion, in our comparative analysis, we investigated CNV, SNV and indel detection efficacy for ev- and cfDNA with respect to tumor biopsy material upon panel sequencing. We were able to demonstrate some benefits for evDNA analysis, such as in-creased fragment size and better sequencing data quality, that were further improved by stringent bioinformatic processing. Although the investigated cohort size was limited, our data suggest that evDNA or in particular a combination of ev- and cfDNA analytes may have benefits for liquid biopsy NGS applications, but further investigation is needed to fully validate and establish their use in the clinical routine.

## Figures and Tables

**Table 1:** Patient data. Patient cohort comprising 10 patients with advanced-stage PDAC.

Patient	Gender	Age	Biopsy Taken from	Tumor Grade	TNM Stage	Metastasis
1	Male	61	Liver	/	cT2 cN+ cM1	Liver
2	Male	43	Liver	/	cT4 cN+ pM1	Local, liver, lung, peritoneum
3	Female	60	Liver	G2	cT3 cN+ cM1	Local, liver, lung
4	Female	32	Lung	/	/	Liver, lung
5	Male	61	Liver	/	cT3 cN+ cM1	Local, liver (multiple)
6	Male	62	Liver	G4	cT4 cN0 pM1	Local, liver (multiple)
7	Male	60	Pancreas	G2	pT3 cN1 cM1	Local, liver, lung, bones
8	Female	58	Liver	/	cT3-4 cN+ cM1	Liver, lung, peritoneum
9	Male	68	Liver	G3	cT3-4 cN+ cM2	Liver, lung
10	Female	54	Liver	/	cT3 cN+ cM1	Liver, spleen, stomach

**Table 2:** Quality parameters of sequencing data for tumorDNA.

Patient (tumorDNA)	Number of Mapped Reads (in Million)	Proportion of Sequenced Reads (in %)	Median Insert Size	Average Coverage
1	80.700	63.3	168	835.6
2	Not enough DNA to pass quality control for sequencing			
3	97.104	60.2	169	1001.1
4	111.257	66.9	168	1263.4
5	92.790	59.9	166	901.4
6	91.009	64.3	175	986.0
7	56.938	55.7	131	327.7
8	93.552	61.0	158	927.2
9	111.344	68.0	169	1206.1
10	105.295	68.3	167	1153.6
**Mean**	**93.332**	**63.1**	**163**	**955.8**

**Table 3:** Quality parameters of sequencing data for ev- and cfDNA.

Patient	Number of Mapped Reads (in Million)	Proportion of Sequenced Reads (in %)	Median Insert Size	Average Coverage
1	76.142	75.4	164	838.2
2	86.320	72.0	170	917.7
3	131.533	67.3	174	1404.9
4	81.173	77.1	178	869.6
5	71.287	75.4	174	771.6
6	67.681	65.9	179	630.8
7	82.874	73.6	165	904.7
8	80.640	63.0	170	803.6
9	73.149	63.6	174	723.9
10	87.771	71.7	172	933.6
**Mean (evDNA)**	**83.857**	**70.5**	**172**	**879.9**
1	75.039	73.9	162	833.7
2	80.430	71.7	167	889.0
3	76.728	75.3	163	902.6
4	75.712	66.1	164	798.8
5	73.376	72.2	168	851.8
6	72.949	64.5	166	672.2
7	82.374	68.1	163	922.6
8	73.116	67.6	165	758.0
9	76.150	69.2	166	809.3
10	137.801	68.0	162	1513.2
**Mean (cfDNA)**	**82.368**	**69.7**	**165**	**895.1**

**Table 4:** Analysis of actionable variants tier 1 + 2 detected in filtered SNVs (AF ≤ 1%; impact: moderate/high) of tumor-, ev- and cfDNA utilizing different databases.

Patient	Variant	ClinVar	Varsome	OncoKB	Level of Evidence (According to OncoKB)
1	IDH2 R261H (tumor, **ev**, **cf**)	Benign/likely benign (VCV000211177.4)	Benign	Unknown effect	/
	IDH2 R131H (tumor, **ev**, **cf**)	Benign/likely benign (VCV000211177.5)	Benign	Unknown effect	/
	IDH2 R209H (tumor, **ev**, **cf**)	Benign/likely benign (VCV000211177.5)	Benign	Unknown effect	/
	RAD51B T107K (tumor)	/	Uncertain significance	Unknown effect	/
	TP53 Y220N (tumor)	Conflicting interpretations of pathogenicity; pathogenic (1), likely pathogenic (1), uncertain significance (1) (VCV000376688.7)	Pathogenic	Likely oncogenic (likely loss of function)	/
	TP53 Y127N (tumor)	/	Pathogenic	Likely oncogenic (likely loss of function)	/
	TP53 Y88N (tumor)	Conflicting interpretations of pathogenicity; pathogenic (1), likely pathogenic (1), uncertain significance (1) (VCV000376688.7)	Pathogenic	Unknown effect	/
2	BRCA2 E394A (**ev**, **cf**)	Benign (VCV000051077)	Likely benign	Unknown effect	/
	BRIP1 P47A (**ev**, **cf**)	Conflicting interpretations of pathogenicity; benign (3), uncertain significance (13) (VCV000004736.28)	Likely pathogenic	Oncogenic (loss of function)	Therapeutic: Level 3B (clinical evidence) FDA Level 2 (prostate cancer and NOS: olaparib)
3	BRAF L319I (**ev**)	/	/	Unknown effect	/
	TP53 R141H (tumor, **ev**, **cf**)	Pathogenic (VCV000012366.20)	Pathogenic	Likely oncogenic (likely loss of function)	/
4	ATM E2156D (tumor)	/	Uncertain significance (VUS with minor pathogenic evidence)	Unknown effect	/
	BRAF L319I (**ev**)	/	/	Unknown effect	/
	PTEN Y155C (tumor, **ev**, **cf**)	Pathogenic (VCV000404168)	Pathogenic	Oncogenic (loss of function)	Therapeutic: Level 4 (biological evidence) FDA level 3 (all solid tumors: AZD8186, GSK2636771)
	ROS1 G374A (tumor, **ev**, **cf**)	/	Uncertain significance (VUS with minor pathogenic evidence)	Unknown effect	/
	ROS1 G365A (tumor, **ev**, **cf**)	Likely pathogenic (VCV000402166.1)	Uncertain significance (VUS with minor pathogenic evidence)	Unknown effect	/
	TP53 L17R (tumor, **ev**, **cf**)	/	/	Unknown effect	/
	TP53 L198R (tumor, **ev**, **cf**)	/	Uncertain significance	Likely oncogenic (likely loss of function)	/
	TP53 L330R (tumor, **ev**, **cf**)	/	Likely pathogenic	Likely oncogenic (likely loss of function)	/
5	BARD1 R207C (tumor, **ev**, **cf**)	Conflicting interpretations of pathogenicity; benign (7), likely benign (4), uncertain significance (1) (VCV000136500.20)	Benign	Unknown effect	/
	TP53 V125G (tumor)	/	Pathogenic	Likely oncogenic (likely loss of function)	/
	TP53 V86G (tumor)	Uncertain significance (VCV000528249.2)	Pathogenic	Unknown effect	/
	TP53 V218G (tumor)	Uncertain significance (VCV000528249.2)	Pathogenic	Likely oncogenic (likely loss of function)	/
6	RAD51B T107K (tumor)	/	Uncertain significance	Unknown effect	/
	ALK R405H (**cf**)	/	Uncertain significance (VUS with minor pathogenic evidence)	Unknown effect	/
	ALK R1575H (**cf**)	Uncertain significance (VCV000579141.4)	Uncertain significance (VUS with minor pathogenic evidence)	Unknown effect	/
	ATM L89F (**cf**)	/	Benign	Unknown effect	/
7	ATM L89F (tumor, **ev**, **cf**)	/	Benign	Unknown effect	/
	BRAF L319I (tumor)	/	/	Unknown effect	/
	CHEK2 K373E (tumor)	Conflicting interpretations of pathogenicity; benign (1), uncertain significance (4) (VCV000481100.6)	Uncertain significance (VUS with minor pathogenic evidence)	Oncogenic (loss of function)	Therapeutic: Level 3B (clinical evidence) FDA Level 2 (prostate cancer and NOS: olaparib)
	CHEK2 K117E (tumor)	/	/	Unknown effect	/
	CHEK2 K344E CHEK2 K152E CHEK2 K416E (tumor)	Conflicting interpretations of pathogenicity; benign (1), uncertain significance (4) (VCV000481100.6)	Uncertain significance	Unknown effect	/
	CHEK2 K282E (tumor)	Uncertain significance (VCV000182433.4)	Uncertain significance	Unknown effect	/
	FGFR3 P449S (tumor, **ev**, **cf**)	Benign/likely benign (VCV000134409.8)	Benign	Unknown effect	/
	FGFR3 P450S FGFR3 P451S (tumor, **ev**, **cf**)	Benign/likely benign (VCV000134409.10)	Benign	Unknown effect	/
	NF1 D109E (tumor, **ev**, **cf**)	/	/	Unknown effect	/
	RAD51B T107K (tumor)	/	Uncertain significance	Unknown effect	/
	TP53 V157F (tumor)	Conflicting interpretations of pathogenicity; likely pathogenic (2), uncertain significance (1) (VCV000012353.8)	Pathogenic	Likely oncogenic (likely loss of function)	/
	TP53 V25F (tumor)	Conflicting interpretations of pathogenicity; likely pathogenic (2), uncertain significance (1) (VCV000012353.8)	Pathogenic	Unknown effect	/
	TP53 V64F (tumor)	/	Pathogenic	Unknown effect	/
8	BRAF L319I (tumor)	/	/	Unknown effect	/
9	BRAF L319I (**ev**)	/	/	Unknown effect	/
	TP53 Y163C (tumor)	Pathogenic (VCV000127814.9)	Pathogenic	Likely oncogenic (loss of function)	/
	TP53 Y31C (tumor)	Pathogenic (VCV000127814.9)	Pathogenic	Unknown effect	/
	TP53 Y70C (tumor)	/	Pathogenic	Unknown effect	/
10	ATM P1054R (tumor, **ev**, **cf**)	Benign/likely benign (VCV000132695)	Benign	Likely neutral	/
	BRAF L319I (**ev**)	/	/	Unknown effect	/
	PTEN Y27C (tumor, **ev**, **cf**)	Likely pathogenic (VCV000404160)	Pathogenic	Likely oncogenic (likely loss of function)	Therapeutic: Level 4 (biological evidence) (all solid tumors: AZD8186, GSK2636771)
	RAD51B T107K (**ev**)	/	Uncertain significance	Unknown effect	/
	TP53 S127Y (tumor, **ev**, **cf**)	Pathogenic (VCV000656751.2)	Pathogenic	Likely oncogenic (likely loss of function)	/
	TP53 S34Y (tumor, **ev**, **cf**)	/	Pathogenic	Unknown effect	/

## Data Availability

Data are either available by download from the SRA database (PRJNA803199) or provided as a supplemental file attached to this manuscript or may be requested from the authors.

## Supplementary Materials

The following supporting information can be downloaded at: https://www.mdpi.com/article/10.3390/cancers14041074/s1, File S1: Patient 1_CNV call from cfDNA; File S2: Patient 1_CNV call from evDNA; File S3: Patient 1_CNV call from tumorDNA; File S4: Patient 2_CNV call from cfDNA; File S5: Patient 2_CNV call from evDNA; File S6: Patient 3_CNV call from cfDNA; File S7: Patient 3_CNV call from evDNA; File S8: Patient 3_CNV call from tumorDNA; File S9: Patient 4_CNV call from cfDNA; File S10: Patient 4_CNV call from evDNA; File S11: Patient 4_CNV call from tumorDNA; File S12: Patient 5_CNV call from cfDNA; File S13: Patient 5_CNV call from evDNA; File S14: Patient 5_CNV call from tumorDNA; File S15: Patient 6_CNV call from cfDNA; File S16: Patient 6_CNV call from evDNA; File S17: Patient 6_CNV call from tumorDNA; File S18: Patient 7_CNV call from cfDNA; File S19: Patient 7_CNV call from evDNA; File S20: Patient 7_CNV call from tumorDNA; File S21: Patient 8_CNV call from cfDNA; File S22: Patient 8_CNV call from evDNA; File S23: Patient 8_CNV call from tumorDNA; File S24: Patient 9_CNV call from cfDNA; File S25: Patient 9_CNV call from evDNA; File S26: Patient 9_CNV call from tumorDNA; File S27: Patient 10_CNV call from cfDNA; File S28: Patient 10_CNV call form evDNA; File S29: Patient 10_CNV call from tumorDNA; File S30: Patient 1_indel call from cfDNA_vcf; File S31: Patient 1_SNV call from cfDNA_vcf; File S32: Patient 1_indel call from evDNA_vcf; File S33: Patient 1_SNV call from evDNA_vcf; File S34: Patient 1_indel call from tumorDNA_vcf; File S35: Patient 1_SNV call from tumorDNA_vcf; File S36: Patient 2_indel call from cfDNA_vcf; File S37: Patient 2_SNV call from cfDNA_vcf; File S38: Patient 2_ indel call from evDNA_vcf; File S39: Patient 2_SNV call from evDNA_vcf; File S40: Patient 3_ indel call from cfDNA_vcf; File S41: Patient 3_ SNV call from cfDNA_vcf; File S42: Patient 3_ indel call from evDNA_vcf; File S43: Patient 3_ SNV call from evDNA_vcf; File S44: Patient 3_ indel call from tumorDNA_vcf; File S45: Patient 3_ SNV call from tumorDNA_vcf; File S46: File S46_ indel call from Patient 4_cfDNA_vcf; File S47: Patient 4_ SNV call from cfDNA_vcf; File S48: Patient 4_ indel call from evDNA_vcf; File S49: Patient 4_ SNV call from evDNA_vcf; File S50: Patient 4_ indel call from tumorDNA_vcf; File S51: Patient 4_ SNV call from tumorDNA_vcf; File S52: Patient 5_ indel call from cfDNA_vcf; File S53: Patient 5_ SNV call from cfDNA_vcf; File S54: Patient 5_ indel call from evDNA_vcf; File S55: Patient 5_ SNV call from evDNA_vcf; File S56: Patient 5_ indel call from tumorDNA_vcf; File S57: Patient 5_ SNV call from tumorDNA_vcf; File S58: Patient 6_ indel call from cfDNA_vcf; File S59: Patient 6_ SNV call from cfDNA_vcf; File S60: Patient 6_ indel call from evDNA_vcf; File S61: Patient 6_ SNV call from evDNA_vcf; File S62: Patient 6_ indel call from tumorDNA_vcf; File S63: Patient 6_ SNV call from tumorDNA_vcf; File S64: Patient 7_ indel call from cfDNA_vcf; File S65: Patient 7_ SNV call from cfDNA_vcf; File S66: Patient 7_ indel call from evDNA_vcf; File S67: Patient 7_ SNV call from evDNA_vcf; File S68: Patient 7_ indel call from tumorDNA_vcf; File S69: Patient 7_ SNV call from tumorDNA_vcf; File S70: Patient 8_ indel call from cfDNA_vcf; File S71: Patient 8_ SNV call from cfDNA_vcf; File S72: Patient 8_ indel call from evDNA_vcf; File S73: Patient 8_ SNV call from evDNA_vcf; File S74: Patient 8_ indel call from tumorDNA_vcf; File S75: Patient 8_ SNV call from tumorDNA_vcf; File S76: Patient 9_ indel call from cfDNA_vcf; File S77: Patient 9_ SNV call from cfDNA_vcf; File S78: Patient 9_ indel call from evDNA_vcf; File S79: Patient 9_ SNV call from evDNA_vcf; File S80: Patient 9_ indel call from tumorDNA_vcf; File S81: Patient 9_ SNV call from tumorDNA_vcf; File S82: Patient 10_ indel call from cfDNA_vcf; File S83: Patient 10_ SNV call from cfDNA_vcf; File S84: Patient 10_ indel call from evDNA_vcf; File S85: Patient 10_ SNV call from evDNA_vcf; File S86: Patient 10_ indel call from tumorDNA_vcf; File S87: Patient 10_ SNV call from tumorDNA_vcf; Table S1: Tumor-gene panel; Table S2: Additive patient data; Table S3: Exemplary patient data; Table S4: Healthy proband data; Table S5: Variant effect prediction for tumorDNA; Table S6: Variant effect prediction for ev- and cfDNA; Table S7: Variants detected across all patients and samples; Table S8: Overview of BRCA variants; Table S9: BRCA1 variants per patient; Table S10: BRCA2 variants per patient; Table S11: Detected BRCA variants in CNVs; Table S12: Tier 1 + 2 actionable variants in tumorDNA; Table S13: Tier 1 + 2 actionable variants in evDNA; Table S14: Tier 1 + 2 actionable variants in cfDNA; Table S15: Tier 1 + 2 actionable variants in tumorDNA utilizing the COSMIC database; Table S16: Tier 1 + 2 actionable variants in evDNA utilizing the COSMIC database; Table S17: Tier 1 + 2 actionable variants in cfDNA utilizing the COSMIC database; Table S18: Tier 1 + 2 actionable variants in indels of tumorDNA; Table S19: Tier 1 + 2 actionable variants in indels of evDNA; Table S20: Tier 1 + 2 actionable variants in indels of cfDNA; Figure S1: Coverage and variant calling; Figure S2: Chromosomal positions of SNVs for patient 1; Figure S3: Chromosomal positions of SNVs for patient 2; Figure S4: Chromosomal positions of SNVs for patient 3; Figure S5: Chromosomal positions of SNVs for patient 4; Figure S6: Chromosomal positions of SNVs for patient 5; Figure S7: Chromosomal positions of SNVs for patient 6; Figure S8: Chromosomal positions of SNVs for patient 7; Figure S9: Chromosomal positions of SNVs for patient 8; Figure S10: Chromosomal positions of SNVs for patient 9; Figure S11: Chromosomal positions of SNVs for patient 10; Figure S12: Agreement of CNVs between ev- and tumorDNA as well as between cf- and tumorDNA; Figure S13: Overview of detected CNVs; Figure S14: Agreement of SNVs between ev- and tumorDNA as well as between cf- and tumorDNA; Figure S15: Representative alteration plots for variants detected in tumor- and ev- or cfDNA; Figure S16: Analysis of SNVs for patient 1; Figure S17: Analysis of SNVs for patient 2; Figure S18: Analysis of SNVs for patient 3; Figure S19: Analysis of SNVs for patient 4; Figure S20: Analysis of SNVs for patient 5; Figure S21: Analysis of SNVs for patient 6; Figure S22: Analysis of SNVs for patient 7; Figure S23: Analysis of SNVs for patient 8; Figure S24: Analysis of SNVs for patient 9; Figure S25: Analysis of SNVs for patient 10; Figure S26: Analysis of indels for patient 1; Figure S27: Analysis of indels for patient 2; Figure S28: Analysis of indels for patient 3; Figure S29: Analysis of indels for patient 4; Figure S30: Analysis of indels for patient 5; Figure S31: Analysis of indels for patient 6; Figure S32: Analysis of indels for patient 7; Figure S33: Analysis of indels for patient 8; Figure S34: Analysis of indels for patient 9; Figure S35: Analysis of indels for patient 10; Figure S36: Analysis of actionable variants; Figure S37: Western blot raw data showing all bands and molecular weight markers.

## Abbreviations

AUC: area under the curve; bp: base pair; RT: room temperature.
