# Supplementary material for: Comparative Panel Sequencing of DNA Variants in cf-, ev- and tumorDNA for Pancreatic Ductal Adenocarcinoma Patients
Source: Cancers (Basel). 2022 Feb 21;14(4):1074. doi: 10.3390/cancers14041074 (PMC8870073; doi:10.3390/cancers14041074)
Supplement: Supplementary file 1 [file cancers-14-01074-s001.zip › cancers-1571843 supplementary.pdf]

Supplemental material

Supplemental Table S1: Tumor-gene panel. Gene list of TUM01 panel (CeGaT GmbH) used for DNA sequencing.

**TUM01 panel: Gene list (766 genes)**
AAK1, ABCB1, ABCG2, ABL1, ABL2, ABRAXAS1, ACD, ACVR1, ADGRA2, ADRB1, ADRB2, AIP, AIRE, AJUBA, AKT1, AKT2, AKT3, ALK,
ALOX12B, AMER1, ANKRD26, APC, APLNR, APOBEC3A, APOBEC3B, AR, ARAF, ARHGAP35, ARID1A, ARID1B, ARID2, ARID5B, ASXL1,
ASXL2, ATM, ATP1A1, ATR, ATRX, AURKA, AURKB, AURKC, AXIN1, AXIN2, AXL, B2M, BAP1, BARD1, BAX, BCHE, BCL10, BCL11A,
BCL11B, BCL2, BCL3, BCL6, BCL9, BCL9L, BCOR, BCORL1, BCR, BIRC2, BIRC3, BIRC5, BLM, BMI1, BMPR1A, BRAF, BRCA1, BRCA2, BRD3,
BRD4, BRD7, BRIP1, BTK, BUB1B, CALR, CAMK2G, CARD11, CASP8, CBFB, CBL, CBLB, CBLC, CCDC6, CCND1, CCND2, CCND3, CCNE1,
CD274, CD79A, CD79B, CD82, CDC73, CDH1, CDH11, CDH2, CDH5, CDK1, CDK12, CDK4, CDK5, CDK6, CDK8, CDKN1A, CDKN1B, CDKN1C,
CDKN2A, CDKN2B, CDKN2C, CEBPA, CENPA, CEP57, CFTR, CHD1, CHD2, CHD4, CHEK1, CHEK2, CIC, CIITA, CKS1B, CNKSR1, COL1A1,
COMT, COQ2, CREB1, CREBBP, CRKL, CRLF2, CRTC1, CRTC2, CSF1R, CSF3R, CSMD1, CSNK1A1, CTCF, CTLA4, CTNNA1, CTNNB1, CTRC,
CUX1, CXCR4, CYLD, CYP1A2, CYP2A7, CYP2B6, CYP2C19, CYP2C8, CYP2C9, CYP2D6, CYP3A4, CYP3A5, CYP4F2, DAXX, DCC, DDB2, DDR1,
DDR2, DDX11, DDX3X, DDX41, DEK, DHFR, DICER1, DIS3L2, DNMT1, DNMT3A, DOT1L, DPYD, E2F3, EBP, EED, EFL1, EGFR, EGLN1,
EGLN2, EIF1AX, ELAC2, ELF3, EME1, EML4, EMSY, EP300, EPAS1, EPCAM, EPHA2, EPHA3, EPHA4, EPHB4, EPHB6, ERBB2, ERBB3, ERBB4,
ERCC1, ERCC2, ERCC3, ERCC4, ERCC5, ERG, ERRFI1, ESR1, ESR2, ETNK1, ETS1, ETV1, ETV4, ETV5, ETV6, EWSR1, EXO1, EXT1, EXT2, EZH1,
EZH2, FAN1, FANCA, FANCB, FANCC, FANCD2, FANCE, FANCF, FANCG, FANCI, FANCL, FANCM, FAS, FAT1, FBXO11, FBXW7, FEN1,
FES, FGF10, FGF14, FGF19, FGF2, FGF23, FGF3, FGF4, FGF5, FGF6, FGF9, FGFBP1, FGFR1, FGFR2, FGFR3, FGFR4, FH, FLCN, FLI1, FLT1, FLT3,
FLT4, FOXA1, FOXA2, FOXE1, FOXL2, FOXO1, FOXO3, FOXP1, FOXQ1, FRK, FRS2, FUBP1, FUS, FYN, G6PD, GALNT12, GATA1, GATA2,
GATA3, GATA4, GATA6, GGT1, GLI1, GLI2, GLI3, GNA11, GNA13, GNAQ, GNAS, GNB3, GPC3, GPER1, GREM1, GRIN2A, GRM3, GSK3A,
GSK3B, GSTP1, H3-3A, H3-3B, H3C2, HABP2, HCK, HDAC1, HDAC2, HDAC6, HGF, HIF1A, HLA-A, HLA-B, HLA-C, HLA-DPA1, HLA-DPB1,
HLA-DQA1, HLA-DQB1, HLA-DRA, HLA-DRB1, HMGA2, HMGCR, HMGN1, HNF1A, HNF1B, HOXB13, HRAS, HSD3B1, HSP90AA1,
HSP90AB1, HTR2A, ID3, IDH1, IDH2, IDO1, IFNGR1, IFNGR2, IGF1R, IGF2, IGF2R, IKBKB, IKBKE, IKZF1, IKZF3, IL1B, IL1RN, ING4, INPP4A,
INPP4B, INPPL1, INSR, IRF1, IRF2, IRS1, IRS2, IRS4, ITPA, JAK1, JAK2, JAK3, JUN, KAT6A, KDM5A, KDM5C, KDM6A, KDR, KEAP1, KIAA1549,
KIF1B, KIT, KLF2, KLF4, KLHL6, KLLN, KMT2A, KMT2B, KMT2C, KMT2D, KNSTRN, KRAS, KSR1, LATS1, LATS2, LCK, LIG4, LIMK2, LRP1B,
LRRK2, LTK, LYN, LZTR1, MAD2L2, MAF, MAGI1, MAGI2, MAML1, MAP2K1, MAP2K2, MAP2K3, MAP2K4, MAP2K5, MAP2K6, MAP2K7,
MAP3K1, MAP3K13, MAP3K14, MAP3K3, MAP3K4, MAP3K6, MAP3K8, MAPK1, MAPK11, MAPK12, MAPK14, MAPK3, MAX, MBD1, MC1R,
MCL1, MDC1, MDH2, MDM2, MDM4, MECOM, MED12, MEF2B, MEN1, MERTK, MET, MGA, MGMT, MITF, MLH1, MLH3, MLLT10, MLLT3,
MN1, MPL, MRE11, MS4A1, MSH2, MSH3, MSH4, MSH5, MSH6, MSR1, MST1R, MTAP, MTHFR, MTOR, MT-RNR1, MTRR, MUC1, MUTYH,
MXI1, MYB, MYC, MYCL, MYCN, MYD88, MYH11, MYH9, NAT2, NBN, NCOA1, NCOA3, NCOR1, NF1, NF2, NFE2L2, NFKB1, NFKB2,
NFKBIA, NFKBIE, NIN, NKX2-1, NLRC5, NOTCH1, NOTCH2, NOTCH3, NOTCH4, NPM1, NQO1, NR1I3, NRAS, NRG1, NRG2, NSD1, NSD2,
NSD3, NT5C2, NT5E, NTHL1, NTRK1, NTRK2, NTRK3, NUMA1, NUP98, NUTM1, OPRM1, PAK1, PAK3, PAK4, PAK5, PALB2, PALLD, PARP1,
PARP2, PARP4, PAX3, PAX5, PAX7, PBK, PBRM1, PBX1, PDCD1, PDCD1LG2, PDGFA, PDGFB, PDGFC, PDGFD, PDGFRA, PDGFRB, PDIA3,
PDK1, PDPK1, PGR, PHF6, PHOX2B, PIGA, PIK3C2A, PIK3C2B, PIK3C2G, PIK3CA, PIK3CB, PIK3CD, PIK3CG, PIK3R1, PIK3R2, PIK3R3, PIM1,
PKHD1, PLCG1, PLCG2, PLK1, PML, PMS1, PMS2, POLD1, POLE, POLH, POLQ, POT1, PPM1D, PPP2R1A, PPP2R2A, PRDM1, PREX2,
PRKAR1A, PRKCA, PRKCI, PRKD1, PRKDC, PRKN, PRMT5, PRSS1, PSMB1, PSMB10, PSMB2, PSMB5, PSMB8, PSMB9, PSMC3IP, PSME1,
PSME2, PSME3, PSPH, PTCH1, PTCH2, PTEN, PTGS2, PTK2, PTK6, PTK7, PTPN11, PTPN12, PTPRC, PTPRD, PTPRS, PTPRT, RABL3, RAC1,
RAC2, RAD21, RAD50, RAD51, RAD51B, RAD51C, RAD51D, RAD54B, RAD54L, RAF1, RALGDS, RARA, RASA1, RASAL1, RB1, RBM10,
RECQL4, RET, RFC2, RFWD3, RFX5, RFXANK, RFXAP, RHBDF2, RHEB, RHOA, RICTOR, RINT1, RIPK1, RIT1, RNASEL, RNF43, ROS1, RPS20,
RPS6KB1, RPS6KB2, RPTOR, RSF1, RUNX1, RYR1, SAMHD1, SAV1, SBDS, SCG5, SDHA, SDHAF2, SDHB, SDHC, SDHD, SEC23B, SERPINB9,
SETBP1, SETD2, SETDB1, SF3B1, SGK1, SH2B1, SH2B3, SHH, SIK2, SIN3A, SKP2, SLC19A1, SLC26A3, SLCO1B1, SLIT2, SLX4, SMAD3, SMAD4,
SMARCA4, SMARCB1, SMARCD1, SMARCE1, SMC1A, SMC3, SMO, SOCS1, SOX11, SOX2, SOX9, SPEN, SPINK1, SPOP, SPRED1, SPTA1, SRC,

SRD5A2, SRGAP1, SRSF2, SRY, SSTR1, SSTR2, SSX1, STAG1, STAG2, STAT1, STAT3, STAT5A, STAT5B, STK11, SUFU, SUZ12, SYK, TAF1, TAF15,
TAP1, TAP2, TAPBP, TBK1, TBL1XR1, TBX3, TCF3, TCF4, TCF7L2, TCL1A, TEK, TENT5C, TERC, TERF2IP, TERT, TET1, TET2, TFE3, TGFB1,
TGFBR2, TLR4, TLX1, TMEM127, TMPRSS2, TNFAIP3, TNFRSF11A, TNFRSF13B, TNFRSF14, TNFRSF8, TNFSF11, TNK2, TOP1, TOP2A, TP53,
TP53BP1, TP63, TPMT, TPX2, TRAF2, TRAF3, TRAF5, TRAF6, TRAF7, TRRAP, TSC1, TSC2, TSHR, TTK, TUBB, TYMS, U2AF1, UBE2T, UBR5,
UGT1A1, UGT2B15, UGT2B7, UIMC1, UNG, USP34, USP9X, VEGFA, VEGFB, VHL, VKORC1, WRN, WT1, XIAP, XPA, XPC, XPO1, XRCC1, ,
XRCC2, XRCC3, XRCC5, XRCC6, YAP1, YES1, ZFHX3, ZNF217, ZNF703, ZNRF3, ZRSR2
**Detection of selected translocations in these genes**
ALK, BCL2, BCR, BRAF, BRD4, EGFR, ERG, ETV4, ETV6, EWSR1, FGFR1, FGFR2, FGFR3, FUS, MET, MYB, MYC, NOTCH2, NTRK1, PAX3,
PDGFB, RAF1, RARA, RET, ROS1, SSX1, SUZ12, TAF15, TCF3, TFE3, TMPRSS2

Supplemental Table S2: Additive patient data. Additional data of PDAC patient cohort focusing on therapy.

Patient	Pre-therapy	1st therapy	1st PFS (month)	2nd therapy	2nd PFS (month)	3rd therapy	3rd PFS (month)	Overall survival (month)
**1**	FOL	FOL	7	Gem/nab-pac (+ radiothe-rapy)	9	Nal-IRI/5-FU	2	N/A
**2**	FOL, gem/nab-pac	FOL	3	Gem/nab-pac	2	Mitomy-cin/vino-relbine	1	8
**3**	FOL, gem/nab-pac/nal-IRI/5-FU	FOL	9	Gem/nab-pac	8	Nal-IRI/5-FU	3	21
**4**	FOL, Gem/nab	FOL, gem/nab-pac	3	/	/	/	/	3
**5**	FOL, gem/nab-pac/nal-IRI/5-FU	FOL	3	Gem/nab-pac	12	/	/	18
**6**	FOL	FOL	6	/	/	/	/	6
**7**	Gem/nab-pac/ OFF	Nal-IRI/5-FU	10	OFF	4	Dual im-mun-therapy	2	29
**8**	OFF/Rtx/ gem/nab-pac/lap/nal-IRI/5-FU	Gem/nab-pac	2	Nal-IRI/5-FU	1	5-FU, lap	5	14
**9**	FOL/ gem/nab	FOL	7	Gem/nab-pac	/	/	/	/
**10**	Nal-IRI/5-FU, gem/cis	Nal-IRI/5-FU	2	Gem/cis	3	Everolimus	N/A	7

Abbreviations: 5-FU: 5-Fluorouracil; Cis: Cisplatin; FOL: FOLFIRINOX; Gem: Gemcitabine; Lap: Lapatinib; Nab-pac: nanoparticle albumin–bound paclitaxel; Nal-IRI: Liposomal irinotecan; OFF: Oxaliplatin + 5-FU; PFS: Progression-free survival; Rtx: Rituximab

Supplemental Table S3: Exemplary patient data. Information about exemplary PDAC patients utilized for nanoparticle tracking.

Patient	Gender	Age	Biopsy taken from	Tumor grade	TNM stage	Metastasis
**1**	Male	80	Pancreas	G2	pT3 pN1 cM0	/
**2**	Female	43	Liver	G1	pT3 pN1 pM1	Liver, bones, peritoneum
**3**	Male	57	Liver	G3	cT4 cN1 pM1	Liver
**4**	Male	71	Liver	G3	cT4 cN+ pM1	Liver
**5**	Male	60	Liver	/	cT4 cN1 cM1	Liver
**6**	Male	87	Lung	/	cT3 cN1 pM1	Liver, lung
**7**	Female	62	Liver	G2	cT4 cN1 pM1	Liver, lung

Supplemental Table S4: Healthy proband data. Information about gender and age of healthy probands.

Healthy proband	Gender	Age
**1**	Male	30
**2**	Female	58
**3**	Female	31
**4**	Female	30
**5**	Female	33
**6**	Male	30
**7**	Male	26

Supplemental Table S5: Variant effect prediction for tumorDNA. Numbers of detected variants for CNVs, indels, unfiltered and filtered SNVs (AF ≤ 1%).

Patient (tumorDNA)	Number of CNVs	Number of In-dels	Number of SNVs	Number of SNVs (AF ≤ 1%)
**1**	30	9,932	21,096	11,289
**3**	247	9,628	20,698	10,617
**4**	2,018	9,639	24,711	13,610
**5**	135	9,697	21,627	11,782
**6**	333	9,556	23,354	12,747
**7**	511	10,694	36,755	25,104
**8**	415	9,729	25,427	15,275
**9**	1,533	10,239	26,585	16,195
**10**	664	9,694	28,055	17,039
**Mean**	**654**	**9,868**	**25,368**	**14,851**

Supplemental Table S6: Variant effect prediction for ev- and cfDNA. Numbers of detected variants for CNVs, indels, unfiltered and filtered SNVs (AF ≤ 1%).

Patient	Number of CNVs	Number of In-dels	Number of SNVs	Number of SNVs (AF ≤ 1%)
**1**	252	9,565	20,109	10,381
**2**	129	9,839	21,779	10,880
**3**	141	9,302	19,983	9,931
**4**	151	9,853	23,142	11,874
**5**	157	9,170	20,626	10,824
**6**	225	10,122	22,126	11,441
**7**	82	9,611	22,332	10,728
**8**	115	9,611	21,408	11,268
**9**	51	9,986	21,931	11,426
**10**	305	9,468	22,622	11,693
**Mean (evDNA)**	**161**	**9,653**	**21,606**	**11,045**
**1**	205	9,149	19,731	9,984
**2**	154	9,324	21,428	10,603
**3**	295	9,031	19,393	9,463
**4**	283	9,555	21,745	10,848
**5**	254	9,008	19,794	10,072
**6**	180	9,599	21,509	11,393
**7**	204	9,417	22,088	10,517
**8**	160	9,489	20,779	10,759
**9**	101	9,357	20,909	10,586
**10**	191	9,249	21,743	10,889
**Mean (cfDNA)**	**203**	**9,318**	**20,912**	**10,511**

Supplemental Table S7: Variants detected across all patients and samples. Analysis of variants utilizing different databases.

Variant	ClinVar	Varsome	OncoKB
ERCC5 G1053R	Benign (VCV000134172.2)	Benign	Unknown effect
FLT3 T227M	Benign (VCV000134447.3)	Benign	Unknown effect
GGT1 G84S	/	Benign	/
KMT2C C988F	Benign (VCV000403019.1)	Benign	Unknown effect
KMT2C G838S	/	Uncertain significance	Unknown effect
KMT2C I882T	/	Uncertain significance (VUS with minor pathogenic evidence)	Unknown effect
KMT2C R973G	/	Uncertain significance	Unknown effect
KMT2C T316S	/	Likely benign	Unknown effect
KMT2C W858L	Uncertain significance (VCV001192208.1)	Uncertain significance (VUS with minor pathogenic evidence)	Unknown effect
KMT2C Y987H	/	Uncertain significance	Unknown effect
MAP2K3 R293H	Benign (VCV000768859.1)	Likely benign	/
MAP2K3 R94L	/	Likely benign	/
MAP2K3 R96W	/	Likely benign	/
MAP2K3 T222M	Benign (VCV000768851.2)	Likely benign	/
MAP2K3 V339M	Benign (VCV000768861.1)	Likely benign	/
NCOR1 G5V	/	Uncertain significance	Unknown effect
PARP4 A899T	/	Benign	/
PRSS1 C206S	Benign (VCV001169143)	Uncertain significance	Unknown effect

Supplemental Table S8: Overview of BRCA variants: Analysis of BRCA1 and BRCA2 variants utilizing different databases.

Variant	ClinVar	Varsome	OncoKB
BRCA1 D397N	/	Benign	Unknown effect
BRCA1 D646N	Benign (VCV000041808.22)	Benign	Unknown effect
BRCA1 D693N	Benign (VCV000041808.22)	Benign	Unknown effect
BRCA1 E1038G	Benign (VCV000041815.23)	Benign	Unknown effect
BRCA1 K887R	/	Benign	Unknown effect
BRCA1 K1136R	Benign (VCV000041818.23)	Benign	Unknown effect
BRCA1 K1183R	Uncertain significance (VCV000920987); benign (VCV000041818.23)	Benign	Unknown effect
BRCA1 P575L	/	Benign	Unknown effect
BRCA1 P824L	Benign (VCV000041812.24)	Benign	Unknown effect
BRCA1 P871L	Benign (VCV000041812.24)	Benign	Unknown effect
BRCA1 S104G	/	Benign	Unknown effect
BRCA1 S384G	/	Benign	Unknown effect
BRCA1 S430G	/	Benign	Unknown effect
BRCA1 S463G	/	Benign	Unknown effect
BRCA S471G	/	Benign	Unknown effect
BRCA1 S509G	Benign (VCV000041827.23)	Benign	Unknown effect
BRCA1 S1317G	/	Benign	Unknown effect
BRCA1 S1566G	Benign (VCV000041827.23)	Benign	Unknown effect
BRCA1 S1613G	Benign (VCV000041827.23)	Benign	Likely neutral
BRCA1 S1634G	Benign (VCV000041827.23)	Benign	Unknown effect
BRCA1 E742G	Uncertain significance (SCV001177685.2); be-nign (SCV000765906.3)	Benign	Unknown effect
BRCA1 E991G	Benign (VCV000041815.23)	Benign	Unknown effect
BRCA2 N249H	/	Benign	Unknown effect
BRCA2 V2466A	Likely benign (VCV001157128); benign (VCV000133738)	Benign	Unknown effect
BRCA2 N372H	Benign (VCV000009329)	Benign	Likely neutral
BRCA2 A2951T	Benign (VCV000041570.17, 36 submissions)	Benign	Likely neutral

Supplemental Table S9: BRCA1 variants per patient. BRCA1 variants found in unfiltered SNVs of tumor-, ev- and cfDNA.

	**1**			**3**			**4**			**5**			**6**			**7**			**8**			**9**			**10**		
BRCA1-variant	tumor	ev	cf	tumor	ev	cf	tumor	ev	cf	tumor	ev	cf	tumor	ev	cf	tumor	ev	cf	tumor	ev	cf	tumor	ev	cf	tumor	ev	cf
D397N	X	X	X																								
D646N	X	X	X																								
D693N	X	X	X																								
E1038G	X	X	X				X	X	X	X	X	X										X	X	X	X	X	X
K887R	X	X	X				X	X	X	X	X	X										X	X	X	X	X	X
K1136R	X	X	X				X	X	X	X	X	X										X	X	X	X	X	X
K1183R	X	X	X				X	X	X	X	X	X										X	X	X	X	X	X
P575L	X	X	X				X	X	X	X	X	X			X							X	X	X	X	X	X
P824L	X	X	X				X	X	X	X	X	X			X							X	X	X	X	X	X
P871L	X	X	X				X	X	X	X	X	X			X							X	X	X	X	X	X
S104G	X	X	X				X	X	X	X	X	X										X	X	X	X	X	X
S384G	X	X	X				X	X	X	X	X	X										X	X	X	X	X	X
S430G	X	X	X				X	X	X	X	X	X										X	X	X	X	X	X
S463G	X	X	X				X	X	X	X	X	X										X	X	X	X	X	X
S471G	X	X	X				X	X	X	X	X	X										X	X	X	X	X	X
S509G	X	X	X				X	X	X	X	X	X										X	X	X	X	X	X
S1317G	X	X	X				X	X	X	X	X	X										X	X	X	X	X	X
S1566G	X	X	X				X	X	X	X	X	X										X	X	X	X	X	X
S1613G	X	X	X				X	X	X	X	X	X										X	X	X	X	X	X
S1634G	X	X	X				X	X	X	X	X	X										X	X	X	X	X	X
E742G	X	X	X				X	X	X	X	X	X										X	X	X	X	X	X
E991G	X	X	X				X	X	X	X	X	X										X	X	X	X	X	X

Supplemental Table S10: BRCA2 variants per patient. BRCA2 variants found in unfiltered SNVs of tumor-, ev- and cfDNA.

	1			3			4			5			6			7			8			9			10		
BRCA2-variant	tumor	ev	cf	tumor	ev	cf	tumor	ev	cf	tumor	ev	cf	tumor	ev	cf	tumor	ev	cf	tumor	ev	cf	tumor	ev	cf	tumor	ev	cf
N249H							X	X	X				X	X	X	X	X	X	X	X	X	X	X	X			
V2466A	X	X	X	X	X	X	X	X	X	X	X	X	X	X	X	X	X	X	X	X	X	X	X	X	X	X	X
N372H							X	X	X				X	X	X	X	X	X	X	X	X	X	X	X			
A2951T																									X	X	X

Supplemental Table S11: Detected BRCA variants in CNVs. BRCA1 and BRCA2 variants found in tumor-, ev- and cfDNA across all patients.

Patient		Chr	Start	Stop	Gene		Call	Percentage deviation	Call z-score
**1**	tumor	17	41200457	41200457	BRCA1	Exon	Duplication	46	3.8
	ev	/	/	/	/	/	/	/	/
	cf	/	/	/	/	/	/	/	/
**3**	tumor	17	41200457	41200457	BRCA1	Exon	Duplication	57	7.1
	ev	17	41200457	41200457	BRCA1	Exon	Duplication	49	2.5
	cf	13	32900238	32900287	BRCA2	Exon	Heterozygous deletion	-56	-8.6
		13	32918695	32918790	BRCA2	Exon	Heterozygous deletion	-64	-8.6
**4**	tumor	13	32889798	32889814	BRCA2	Exon	Duplication	152	9.1
	ev	17	41200457	41200457	BRCA1	Exon	Duplication	58	4.2
		17	41277199	41277202	BRCA1	Exon	Heterozygous deletion	-46	-6.1
	cf	13	32918695	32918790	BRCA2	Exon	Heterozygous deletion	-46	-6.1
		17	41200457	41200457	BRCA1	Exon	Duplication	67	4.3
		17	41276034	41276113	BRCA1	Exon	Heterozygous deletion	-47	-4.8
**5**	tumor	17	41200457	41200457	BRCA1	Exon	Duplication	54	4.0
	ev	17	41276034	41276113	BRCA1	Exon	Heterozygous deletion	-53	-3.7
	cf	13	32889798	32889814	BRCA2	Exon	Heterozygous deletion	-47	-3.3
		13	32890557	32890561	BRCA2	Exon	Heterozygous deletion	-48	-6.3
		17	41276034	41276113	BRCA1	Exon	Heterozygous deletion	-54	-4.0
**6**	tumor	13	32889798	32889814	BRCA2	Exon	Duplication	62	5.5
	ev	17	41200457	41200457	BRCA1	Exon	Duplication	63	2.2
	cf	13	32918695	32918790	BRCA2	Exon	Heterozygous deletion	-45	-6.8
		17	41276034	41276113	BRCA1	Exon	Heterozygous deletion	-53	-5.9
**7**	tumor	13	32889798	32889814	BRCA2	Exon	Duplication	72	6.3
		17	41198104	41198104	BRCA1	Exon	Duplication	175	24.7
		17	41200457	41200457	BRCA1	Exon	Duplication	205	11.8
	ev	13	32918695	32918790	BRCA2	Exon	Heterozygous deletion	-52	-5.4
		17	41276034	41276113	BRCA1	Exon	Heterozygous deletion	-51	-6.1
	cf	/	/	/	/	/	/	/	/
**8**	tumor	13	32889798	32889814	BRCA2	Exon	Duplication	83	9.1
		17	41200457	41200457	BRCA1	Exon	Duplication	51	7.6
	ev	17	41200457	41200457	BRCA1	Exon	Duplication	68	4.3
	cf	/	/	/	/	/	/	/	/
**9**	tumor	13	32920964	32921033	BRCA2	Exon	Heterozygous deletion	-46	-2.8

		17	41199660	41199720	BRCA1	Exon	Duplication	45	3.2
		17	41200457	41200457	BRCA1	Exon	Duplication	86	2.4
	ev	17	41200457	41200457	BRCA1	Exon	Duplication	56	2.6
	cf	/	/	/	/	/	/	/	/
10	tumor	13	32889798	32889814	BRCA2	Exon	Duplication	60	6.1
		13	32900238	32900287	BRCA2	Exon	Heterozygous deletion	-45	-9.2
		17	41258473	41258552	BRCA1	Exon	Heterozygous deletion	-48	-6.4
		17	41258563	41258573	BRCA1	Exon	Heterozygous deletion	-53	-8.2
	ev	17	41258563	41258573	BRCA1	Exon	Heterozygous deletion	-47	-10.5
	cf	/	/	/	/	/	/	/	/

Agreements between tumor- and ev- as well as tumor and cfDNA are marked in red

Supplemental Table S12: Tier 1+2 actionable variants in tumorDNA. Analysis of actionable variants tier 1 and 2 detected in filteredSNVs(AF≤1%;impact:moderate/high)per patient.

Patient (tumorDNA)	SNVs: AF ≤1 % (impact: moderate/high)	Actionable variants tier 1-4 (PP-2 score: damaging)	Actionable variants tier 1+2 (PP-2 score: damaging)	Variant
**1**	645	19	7	IDH2 R261H<br>IDH2 R131H<br>IDH2 R209H<br>RAD51B T107K<br>TP53 Y220N<br>TP53 S127N<br>TP53 Y88N
**2**	/	/	/	/
**3**	607	2	1	TP53 R141H
**4**	599	15	7	ATM E2156D<br>PTEN Y155C<br>ROS1 G374A<br>ROS1 G365A<br>TP53 L17R<br>TP53 L198R<br>TP53 L330R
**5**	614	13	4	BARD1 R207C<br>TP53 V125G<br>TP53 V86G<br>TP53 V218G
**6**	634	1	1	RAD51B T107K
**7**	841	31	16	ATM L89F<br>BRAF L319I<br>CHEK2 K373E<br>CHEK2 K117E<br>CHEK2 K344E<br>CHEK2 K152E<br>CHEK2 K282E<br>CHEK2 K416E<br>FGFR3 P449S<br>FGFR3 P450S<br>FGFR3 P451S<br>NF1 D109E<br>RAD51B T107K<br>TP53 V157F<br>TP53 V25F<br>TP53 V64F
**8**	683	10	1	BRAF L319I
**9**	682	19	3	TP53 Y163C<br>TP53 Y31C<br>TP53 Y70C
**10**	674	14	4	ATM P1054R<br>PTEN Y27C<br>TP53 S127Y<br>TP53 S34Y
**Mean**	**664**	**14**	**5**	

Agreements between tumor- and ev- as well as tumor- and cfDNA are marked in red

Supplemental Table S13: Tier 1+2 actionable variants in evDNA. Analysis of actionable variants tier 1 and 2 detected in filtered SNVs (AF ≤ 1%; impact: moderate/high) per patient.

Patient (evDNA)	SNVs: AF ≤1 % (impact: moderate/high)	Actionable variants tier 1-4 (PP-2 score: damaging)	Actionable variants tier 1+2 (PP-2 score: damaging)	Variant
**1**	572	9	3	IDH2 R261H<br>IDH2 R131H<br>IDH2 R209H
**2**	611	3	2	BRCA2 E394A<br>BRIP1 P47A
**3**	561	9	2	BRAF L319I<br>TP53 R141H
**4**	643	25	7	BRAF L319I<br>PTEN Y155C<br>ROS1 G374A<br>ROS1 G365A<br>TP53 L17R<br>TP53 L198R<br>TP53 L330R
**5**	597	14	1	BARD1 R207C
**6**	655	4	0	/
**7**	666	14	5	ATM L89F<br>FGFR3 P449S<br>FGFR3 P450S<br>FGFR3 P451S<br>NF1 D109E
**8**	663	7	0	/
**9**	641	15	1	BRAF L319I
**10**	616	12	6	ATM P1054R<br>BRAF L319I<br>PTEN Y27C<br>RAD51B T107K<br>TP53 S127Y<br>TP53 S34Y
**Mean**	**623**	**11**	**3**	

Agreements between tumor- and evDNA are marked in red

Supplemental Table S14: Tier 1+2 actionable variants in cfDNA. Analysis of actionable variants tier 1 and 2 detected in filtered SNVs (AF ≤ 1%; impact: moderate/high) per patient.

Patient (cfDNA)	SNVs: AF ≤1 % (impact: moderate/high)	Actionable variants tier 1-4 (PP-2 score: damaging)	Actionable variants tier 1+2 (PP-2 score: damaging)	Variant
**1**	566	9	3	IDH2 R261H<br>IDH2 R131H<br>IDH2 R209H
**2**	592	2	2	BRCA2 E394A<br>BRIP1 P47A
**3**	537	1	1	TP53 R141H
**4**	595	19	6	PTEN Y155C<br>ROS1 G374A<br>ROS1 G365A<br>TP53 L17R<br>TP53 L198R<br>TP53 L330R
**5**	557	9	1	BARD1 R207C
**6**	629	7	3	ALK R405H<br>ALK R1575H<br>ATM L89F
**7**	662	14	5	ATM L89F<br>FGFR3 P449S<br>FGFR3 P450S<br>FGFR3 P451S<br>NF1 D109E
**8**	619	4	0	/
**9**	555	10	0	/
**10**	587	14	4	ATM P1054R<br>PTEN Y27C<br>TP53 S127Y<br>TP53 S34Y
**Mean**	**590**	**9**	**3**	

Agreements between tumor- and cfDNA are marked in red

Supplemental Table S15: Tier 1+2 actionable variants in tumorDNA utilizing the COSMIC database. Analysis of actionable var-iants tier 1+2 detected in filtered SNVs (AF ≤ 1%; impact: moderate/high)

Patient (tum-orDNA)	Actionability gene	Variant	Specific vari-ant/unspeci-fied (according to COSMIC)	Treatment options (FDA approved) suggested by COSMIC	Actionability rank	Disease
**1**	IDH2	R261H R131H R209H	Unspecified	Enasidenib	1	Haemato-poietic and lymphoid tissue / haemato-poietic neo-plasm / acute myeloid leukaemia
	RAD51B	T107K	Unspecified	Olaparib	1	Prostate / carcinoma / NS
	TP53	Y220N S127N Y88N	Unspecified	Gemcitabine + Nab-Paclitaxel	3 (Phase 2)	Pancreas / carcinoma / NS
**2**	/	/	/	/	/	/
**3**	TP53	R141H	Unspecified	Gemcitabine + Nab-Paclitaxel	3 (Phase 2)	Pancreas / carcinoma / NS
**4**	ATM	E2156D	Unspecified	Olaparib	1	Prostate / carcinoma / NS
				Olaparib + Selu-metinib; Epa-cadostat + Pem-brolizumab	3 (Phase 2)	Pancreas / carcinoma / NS
				Olaparib + Pembrolizumab	3 (Phase 2)	Pancreas / carcinoma / ductal car-cinoma
	PTEN	Y155C	Unspecified	MK2206	3 (Phase 2)	Pancreas / carcinoma / ductal car-cinoma
	ROS1	G374A G365A	Unspecified	Crizotinib, Ent-recitinib	1	Lung / car-cinoma / non small cell carci-noma
	TP53	L17R L198R L330R	Unspecified	Gemcitabine + Nab-Paclitaxel	3 (Phase 2)	Pancreas / carcinoma / NS

**5**	BARD1	R207C	Unspecified	Olaparib	1	Prostate / carcinoma / NS
				Olaparib + Pembrolizumab	3 (Phase 2)	pancreas / carcinoma / ductal carcinoma
	TP53	V125G<br>V86G<br>V218G	Unspecified	Gemcitabine + Nab-Paclitaxel	3 (Phase 2)	Pancreas / carcinoma / NS
**6**	RAD51B	T107K	Unspecified	Olaparib	1	Prostate / carcinoma / NS
**7**	ATM	L89F	Unspecified	Olaparib	1	Prostate / carcinoma / NS
				Olaparib + Selumetinib; E-pacadostat + Pembrolizumab	3 (Phase 2)	Pancreas / carcinoma / NS
				Olaparib + Pembrolizumab	3 (Phase 2)	Pancreas / carcinoma / ductal carcinoma
	BRAF	L319I	Unspecified	/	/	/
	CHEK2	K373E<br>K117E<br>K344E<br>K152E<br>K416E<br>K282E	Unspecified	Olaparib	1	Prostate / carcinoma / NS
				Olaparib + Pembrolizumab	3 (Phase 2)	Pancreas / carcinoma / ductal carcinoma
	FGFR3	P449S<br>P450S<br>P451S	Unspecified	/	/	/
	NF1	D109E	Unspecified	/	/	/
	RAD51B	T107K	Unspecified	Olaparib	1	Prostate / carcinoma / NS
	TP53	V157F<br>V25F<br>V64F	Unspecified	Gemcitabine + Nab-Paclitaxel	3 (Phase 2)	Pancreas / carcinoma / NS
**8**	BRAF	L319I	Unspecified	/	/	/
**9**	TP53	Y163C<br>Y31C<br>Y70C	Unspecified	Gemcitabine + Nab-Paclitaxel	3 (Phase 2)	Pancreas / carcinoma / NS
**10**	ATM	P1054R	Unspecified	Olaparib	1	Prostate / carcinoma / NS
				Olaparib + Selumetinib; E-pacadostat + Pembrolizumab	3 (Phase 2)	Pancreas / carcinoma / NS

			Olaparib + Pembrolizumab	3 (Phase 2)	Pancreas / carcinoma / ductal carcinoma
PTEN	Y27C	Unspecified	MK2206	3 (Phase 2)	Pancreas / carcinoma / ductal carcinoma
TP53	S127Y S34Y	Unspecified	Gemcitabine + Nab-Paclitaxel	3 (Phase 2)	Pancreas / carcinoma / NS

Supplemental Table S16: Tier 1+2 actionable variants in evDNA utilizing the COSMIC database. Analysis of actionable variants tier 1+2 detected in filtered SNVs (AF ≤ 1%; impact: moderate/high)

Patient (evDNA)	Actionability gene	Variant	Specific variant/unspecified (according to COSMIC)	Treatment options (FDA approved) suggested by COSMIC	Actionability rank	Disease
**1**	IDH2	R261H<br>R131H<br>R209H	Unspecified	Enasidenib	1	Haematopoietic and lymphoid tissue / haematopoietic neoplasm / acute myeloid leukaemia
**2**	BRCA2	E394A	Unspecified	Olaparib	1	Pancreas /carcinoma / NS; breast / carcinoma / NS; fallopian tube / carcinoma / NS; ovary / carcinoma / NS; peritoneum / carcinoma / NS
				Bevacizumab + chemotherapy + Olaparib	1	Fallopian tube / carcinoma/ NS; ovary / carcinoma / NS; peritoneum / carcinoma / NS
				Olaparib	1	Prostate / carcinoma / NS
	BRIP1	P47A	Unspecified	Olaparib + Pembrolizumab	3 (Phase 2)	Pancreas / carcinoma / ductal carcinoma
**3**	BRAF	L319I	Unspecified	/	/	/
	TP53	R141H	Unspecified	Gemcitabine + Nab-Paclitaxel	3 (Phase 2)	Pancreas / carcinoma / NS
**4**	BRAF	L319I	Unspecified	/	/	/

	PTEN	Y155C	Unspecified	MK2206	3 (Phase 2)	Pancreas / carcinoma / ductal carcinoma
	ROS1	G374A G365A	Unspecified	Crizotinib, Entrecitinib	1	Lung / carcinoma / non small cell carcinoma
	TP53	L17R L198R L330R	Unspecified	Gemcitabine + Nab-Paclitaxel	3 (Phase 2)	Pancreas / carcinoma / NS
**5**	BARD1	R207C	Unspecified	Olaparib	1	Prostate / carcinoma / NS
				Olaparib + Pembrolizumab	3 (Phase 2)	pancreas / carcinoma / ductal carcinoma
**6**	/	/	/	/	/	/
**7**	ATM	L89F	Unspecified	Olaparib	1	Prostate / carcinoma / NS
				Olaparib + Selumetinib; E-pacadostat + Pembrolizumab	3 (Phase 2)	Pancreas / carcinoma / NS
				Olaparib + Pembrolizumab	3 (Phase 2)	Pancreas / carcinoma / ductal carcinoma
	FGFR3	P449S P450S P451S	Unspecified	/	/	/
	NF1	D109E	Unspecified	/	/	/
**8**	/	/	/	/	/	/
**9**	BRAF	L319I	Unspecified	/	/	/
**10**	ATM	P1054R	Unspecified	Olaparib	1	Prostate / carcinoma / NS
				Olaparib + Selumetinib; E-pacadostat + Pembrolizumab	3 (Phase 2)	Pancreas / carcinoma / NS
				Olaparib + Pembrolizumab	3 (Phase 2)	Pancreas / carcinoma / ductal carcinoma
	BRAF	L319I	Unspecified	/	/	/
	PTEN	Y27C	Unspecified	MK2206	3 (Phase 2)	Pancreas / carcinoma / ductal carcinoma

RAD51B	T107K	Unspecified	Olaparib	1	Prostate / carcinoma / NS
TP53	S127Y<br>S34Y	Unspecified	Gemcitabine + Nab-Paclitaxel	3 (Phase 2)	Pancreas / carcinoma / NS

Supplemental Table S17: Tier 1+2 actionable variants in cfDNA utilizing the COSMIC database. Analysis of actionable variants tier 1+2 detected in filtered SNVs (AF ≤ 1%; impact: moderate/high)

Patient (cfDNA)	Actionability gene	Variant	Specific variant/unspecified (according to COSMIC)	Treatment options (FDA approved) suggested by COSMIC	Actionability rank	Disease
**1**	IDH2	R261H R131H R209H	Unspecified	Enasidenib	1	Haematopoietic and lymphoid tissue / haematopoietic neoplasm / acute myeloid leukaemia
**2**	BRCA2	E394A	Unspecified	Olaparib	1	Pancreas /carcinoma / NS; breast / carcinoma / NS; fallopian tube / carcinoma / NS; ovary / carcinoma / NS; peritoneum / carcinoma / NS
				Bevacizumab + chemotherapy + Olaparib	1	Fallopian tube / carcinoma/ NS; ovary / carcinoma / NS; peritoneum / carcinoma / NS
				Olaparib	1	Prostate / carcinoma / NS
	BRIP1	P47A	Unspecified	Olaparib + Pembrolizumab	3 (Phase 2)	Pancreas / carcinoma / ductal carcinoma
**3**	TP53	R141H	Unspecified	Gemcitabine + Nab-Paclitaxel	3 (Phase 2)	Pancreas / carcinoma / NS
**4**	PTEN	Y155C	Unspecified	MK2206	3 (Phase 2)	Pancreas / carcinoma / ductal carcinoma
	ROS1	G374A G365A	Unspecified	Crizotinib, Entrecitinib	1	Lung / carcinoma / non small cell carcinoma
	TP53	L17R L198R L330R	Unspecified	Gemcitabine + Nab-Paclitaxel	3 (Phase 2)	Pancreas / carcinoma / NS
**5**	BARD1	R207C	Unspecified	Olaparib	1	Prostate / carcinoma / NS

				Olaparib + Pembrolizumab	3 (Phase 2)	pancreas / carcinoma / ductal carcinoma
**6**	ALK	R405H R1575	Unspecified	Crizotinib, Brigatinib, Alectinib, Lorlatinib, Ceritinib	1	lung / carcinoma / non small cell carcinoma
				Ceritinib + Gemcitabine; Ceritinib + Cisplatin + Gemcitabine; Ceritinib + Gemcitabine + Nab-paclitaxel	3 (Phase 1)	pancreas / carcinoma / ductal carcinoma
	ATM	L89F	Unspecified	Olaparib	1	Prostate / carcinoma / NS
				Olaparib + Selumetinib; Epacadostat + Pembrolizumab	3 (Phase 2)	Pancreas / carcinoma / NS
				Olaparib + Pembrolizumab	3 (Phase 2)	Pancreas / carcinoma / ductal carcinoma
**7**	ATM	L89F	Unspecified	Olaparib	1	Prostate / carcinoma / NS
				Olaparib + Selumetinib; Epacadostat + Pembrolizumab	3 (Phase 2)	Pancreas / carcinoma / NS
				Olaparib + Pembrolizumab	3 (Phase 2)	Pancreas / carcinoma / ductal carcinoma
	FGFR3	P449S P450S P451S	Unspecified	/	/	/
	NF1	D109E	Unspecified	/	/	/
**8**	/	/	/	/	/	/
**9**	/	/	/	/	/	/
**10**	ATM	P1054 R	Unspecified	Olaparib	1	Prostate / carcinoma / NS
				Olaparib + Selumetinib; Epacadostat + Pembrolizumab	3 (Phase 2)	Pancreas / carcinoma / NS
				Olaparib + Pembrolizumab	3 (Phase 2)	Pancreas / carcinoma / ductal carcinoma
	PTEN	Y27C	Unspecified	MK2206	3 (Phase 2)	Pancreas / carcinoma /

					ductal carci-noma
TP53	S34Y S127Y	Unspecified	Gemcitabine + Nab-Paclitaxel	3 (Phase 2)	Pancreas / car-cinoma / NS

Supplemental Table S18: Tier 1+2 actionable variants in indels of tumorDNA. Analysis of tier 1+2 actionable variants in indels of tumorDNA per patient.

Patient (tumorDNA)	Location	Allele	Consequence	Impact	Symbol	Biotype	Exon	Intron	Protein position
1	11:125505377-125505377	A	Frameshift variant	HIGH	CHEK1	Protein coding	6/11	-	223
	11:125505377-125505377	A	Frameshift variant	HIGH	CHEK1	Protein coding	6/12	-	239
	11:125505377-125505377	A	Frameshift variant	HIGH	CHEK1	Protein coding	7/14	-	223
	11:125505377-125505377	A	Frameshift variant	HIGH	CHEK1	Protein coding	7/13	-	223
	11:125505377-125505377	A	Frameshift variant	HIGH	CHEK1	Protein coding	6/13	-	223
	11:125505377-125505377	A	Frameshift variant	HIGH	CHEK1	Protein coding	5/6	-	144
	11:125505377-125505377	A	Frameshift variant	HIGH	CHEK1	Protein coding	7/12	-	223
	9:133759489-133759492	-	Inframe deletion	MODERATE	ABL1	Protein coding	11/11	-	605
	9:133759489-133759492	-	Inframe deletion	MODERATE	ABL1	Protein coding	11/11	-	624
3	9:133759489-133759492	-	Inframe deletion	MODERATE	ABL1	Protein coding	11/11	-	605
	9:133759489-133759492	-	Inframe deletion	MODERATE	ABL1	Protein coding	11/11	-	624
4	11:125505377-125505377	A	Frameshift variant	HIGH	CHEK1	Protein coding	6/11	-	223
	11:125505377-125505377	A	Frameshift variant	HIGH	CHEK1	Protein coding	6/12	-	239
	11:125505377-125505377	A	Frameshift variant	HIGH	CHEK1	Protein coding	7/14	-	223
	11:125505377-125505377	A	Frameshift variant	HIGH	CHEK1	Protein coding	7/13	-	223
	11:125505377-125505377	A	Frameshift variant	HIGH	CHEK1	Protein coding	6/13	-	223
	11:125505377-125505377	A	Frameshift variant	HIGH	CHEK1	Protein coding	5/6	-	144
	11:125505377-125505377	A	Frameshift variant	HIGH	CHEK1	Protein coding	7/12	-	223
	9:133759489-133759492	-	Inframe deletion	MODERATE	ABL1	Protein coding	11/11	-	605
	9:133759489-133759492	-	Inframe deletion	MODERATE	ABL1	Protein coding	11/11	-	624
5	11:125505377-125505377	A	Frameshift variant	HIGH	CHEK1	Protein coding	6/11	-	223
	11:125505377-125505377	A	Frameshift variant	HIGH	CHEK1	Protein coding	6/12	-	239
	11:125505377-125505377	A	Frameshift variant	HIGH	CHEK1	Protein coding	7/14	-	223
	11:125505377-125505377	A	Frameshift variant	HIGH	CHEK1	Protein coding	7/13	-	223

	11:125505377-125505377	A	Frameshift variant	HIGH	CHEK1	Protein coding	6/13	-	223
	11:125505377-125505377	A	Frameshift variant	HIGH	CHEK1	Protein coding	5/6	-	144
	11:125505377-125505377	A	Frameshift variant	HIGH	CHEK1	Protein coding	7/12	-	223
6	17:7578221-7578223	-	Frameshift variant	HIGH	TP53	Protein coding	6/11	-	209
	17:7578221-7578223	-	Frameshift variant	HIGH	TP53	Protein coding	5/9	-	209
	17:7578221-7578223	-	Frameshift variant	HIGH	TP53	Protein coding	5/7	-	209
	17:7578221-7578223	-	Frameshift variant	HIGH	TP53	Protein coding	6/12	-	209
	17:7578221-7578223	-	Frameshift variant	HIGH	TP53	Protein coding	6/11	-	209
	17:7578221-7578223	-	Frameshift variant	HIGH	TP53	Protein coding	6/12	-	209
	17:7578221-7578223	-	Frameshift variant	HIGH	TP53	Protein coding	3/6	-	77
	17:7578221-7578223	-	Frameshift variant	HIGH	TP53	Protein coding	5/6	-	116
	9:133759489-133759492	-	Inframe deletion	MODERATE	ABL1	Protein coding	11/11	-	605
	9:133759489-133759492	-	Inframe deletion	MODERATE	ABL1	Protein coding	11/11	-	624
7	8:38285913-38285916	-	Inframe deletion	MODERATE	FGFR1	Protein coding	3/17	-	43-44
	8:38285913-38285916	-	Inframe deletion	MODERATE	FGFR1	Protein coding	5/19	-	124-125
	8:38285913-38285916	-	Inframe deletion	MODERATE	FGFR1	Protein coding	4/18	-	135-136
	8:38285913-38285916	-	Inframe deletion	MODERATE	FGFR1	Protein coding	4/18	-	132-133
	8:38285913-38285916	-	Inframe deletion	MODERATE	FGFR1	Protein coding	2/16	-	43-44
	8:38285913-38285916	-	Inframe deletion	MODERATE	FGFR1	Protein coding	5/19	-	132-133
	8:38285913-38285916	-	Inframe deletion	MODERATE	FGFR1	Protein coding	5/19	-	165-166
	8:38285913-38285916	-	Inframe deletion	MODERATE	FGFR1	Protein coding	4/4	-	43-44
	8:38285913-38285916	-	Inframe deletion	MODERATE	FGFR1	Protein coding	4/7	-	132-133
	8:38285913-38285916	-	Inframe deletion	MODERATE	FGFR1	Protein coding	3/5	-	43-44
	8:38285913-38285916	-	Inframe deletion	MODERATE	FGFR1	Protein coding	3/4	-	43-44
	9:133759489-133759492	-	Inframe deletion	MODERATE	ABL1	Protein coding	11/11	-	605
	9:133759489-133759492	-	Inframe deletion	MODERATE	ABL1	Protein coding	11/11	-	624

	17:7579470-7579470	G	Frameshift variant	HIGH	TP53	Protein coding	4/11	-	72-73
	17:7579470-7579470	G	Frameshift variant	HIGH	TP53	Protein coding	3/9	-	72-73
	17:7579470-7579470	G	Frameshift variant	HIGH	TP53	Protein coding	3/7	-	72-73
	17:7579470-7579470	G	Frameshift variant	HIGH	TP53	Protein coding	4/12	-	72-73
	17:7579470-7579470	G	Frameshift variant	HIGH	TP53	Protein coding	5/6	-	72-73
	17:7579470-7579470	G	Frameshift variant	HIGH	TP53	Protein coding	4/5	-	72-73
	8:38285913-38285916	-	Inframe deletion	MODERATE	FGFR1	Protein coding	3/17	-	43-44
	8:38285913-38285916	-	Inframe deletion	MODERATE	FGFR1	Protein coding	5/19	-	124-125
**8**	8:38285913-38285916	-	Inframe deletion	MODERATE	FGFR1	Protein coding	4/18	-	135-136
	8:38285913-38285916	-	Inframe deletion	MODERATE	FGFR1	Protein coding	4/18	-	132-133
	8:38285913-38285916	-	Inframe deletion	MODERATE	FGFR1	Protein coding	2/16	-	43-44
	8:38285913-38285916	-	Inframe deletion	MODERATE	FGFR1	Protein coding	5/19	-	132-133
	8:38285913-38285916	-	Inframe deletion	MODERATE	FGFR1	Protein coding	5/19	-	165-166
	8:38285913-38285916	-	Inframe deletion	MODERATE	FGFR1	Protein coding	4/4	-	43-44
	8:38285913-38285916	-	Inframe deletion	MODERATE	FGFR1	Protein coding	4/7	-	132-133
	8:38285913-38285916	-	Inframe deletion	MODERATE	FGFR1	Protein coding	3/5	-	43-44
	8:38285913-38285916	-	Inframe deletion	MODERATE	FGFR1	Protein coding	3/4	-	43-44
**9**	9:133759489-133759492	-	Inframe deletion	MODERATE	ABL1	Protein coding	11/11	-	605
	9:133759489-133759492	-	Inframe deletion	MODERATE	ABL1	Protein coding	11/11	-	624
**10**	9:133759489-133759492	-	Inframe deletion	MODERATE	ABL1	Protein coding	11/11	-	605
	9:133759489-133759492	-	Inframe deletion	MODERATE	ABL1	Protein coding	11/11	-	624

Agreements between ev- and tumorDNA are marked in blue and agreements between cf- and tumorDNA are marked in red; overlappings in the agreement of ev- and cf- with tumorDNA are marked in violet

Supplemental Table S19: Tier 1+2 actionable variants in indels of evDNA. Analysis of tier 1+2 actionable variants in indels of evDNA per patient.

Patient (evDNA)	Location	Allele	Consequence	Impact	Symbol	Biotype	Exon	Intron	Protein position
**1**	-	-	-	-	-	-	-	-	-
**3**	9:133759489-133759492	-	Inframe deletion	MODERATE	ABL1	Protein coding	11/11	-	605
	9:133759489-133759492	-	Inframe deletion	MODERATE	ABL1	Protein coding	11/11	-	624
**4**	11:125505377-125505377	A	Frameshift variant	HIGH	CHEK1	Protein coding	6/11	-	223
	11:125505377-125505377	A	Frameshift variant	HIGH	CHEK1	Protein coding	6/12	-	239
	11:125505377-125505377	A	Frameshift variant	HIGH	CHEK1	Protein coding	7/14	-	223
	11:125505377-125505377	A	Frameshift variant	HIGH	CHEK1	Protein coding	7/13	-	223
	11:125505377-125505377	A	Frameshift variant	HIGH	CHEK1	Protein coding	6/13	-	223
	11:125505377-125505377	A	Frameshift variant	HIGH	CHEK1	Protein coding	5/6	-	144
	11:125505377-125505377	A	Frameshift variant	HIGH	CHEK1	Protein coding	7/12	-	223
	8:38320386-38320688	-	Splice donor variant, 5 prime UTR variant, intron variant	HIGH	FGFR1	Protein coding	1/2	1/1	-
	9:133759489-133759492	-	Inframe deletion	MODERATE	ABL1	Protein coding	11/11		605
	9:133759489-133759492	-	Inframe deletion	MODERATE	ABL1	Protein coding	11/11	-	624
**5**	9:133759489-133759492	-	Inframe deletion	MODERATE	ABL1	Protein coding	11/11	-	605
	9:133759489-133759492	-	Inframe deletion	MODERATE	ABL1	Protein coding	11/11	-	624
**6**	14:68331716-68331718	-	Splice acceptor variant, intron variant	HIGH	RAD51B	Protein coding	-	3/9	-
	14:68331716-68331718	-	Splice acceptor variant, intron variant	HIGH	RAD51B	Protein coding	-	4/10	-
	14:68331716-68331718	-	Splice acceptor variant, intron variant, NMD transcript variant	HIGH	RAD51B	Nonsense mediated decay	-	5/11	-
	14:68331716-68331718	-	Splice acceptor variant, intron variant	HIGH	RAD51B	Protein coding	-	4/4	-
	14:68331716-68331718	-	Splice acceptor variant, intron variant	HIGH	RAD51B	Protein coding	-	4/11	-

	14:68331716-68331718	-	Splice acceptor variant, intron variant, non-coding transcript variant	HIGH	RAD51B	Processed transcript	-	1/3	-
	14:68331716-68331718	-	Splice acceptor variant, intron variant, non-coding transcript variant	HIGH	RAD51B	Processed transcript	-	1/3	-
	17:7578221-7578223	-	Frameshift variant	HIGH	TP53	Protein coding	6/11	-	209
	17:7578221-7578223	-	Frameshift variant	HIGH	TP53	Protein coding	5/9	-	209
	17:7578221-7578223	-	Frameshift variant	HIGH	TP53	Protein coding	5/7	-	209
	17:7578221-7578223	-	Frameshift variant	HIGH	TP53	Protein coding	6/12	-	209
	17:7578221-7578223	-	Frameshift variant	HIGH	TP53	Protein coding	6/11	-	209
	17:7578221-7578223	-	Frameshift variant	HIGH	TP53	Protein coding	3/6	-	77
	17:7578221-7578223	-	Frameshift variant	HIGH	TP53	Protein coding	5/6	-	116
**7**	9:133759489-133759492	-	Inframe deletion	MODERATE	ABL1	Protein coding	11/11	-	605
	9:133759489-133759492	-	Inframe deletion	MODERATE	ABL1	Protein coding	11/11	-	624
**8**	-	-	-	-	-	-	-	-	-
**9**	9:133759489-133759492	-	Inframe deletion	MODERATE	ABL1	Protein coding	11/11	-	605
	9:133759489-133759492	-	Inframe deletion	MODERATE	ABL1	Protein coding	11/11	-	624
**10**	-	-	-	-	-	-	-	-	-

Agreements between ev- and tumorDNA are marked in blue

Supplemental Table S20: Tier 1+2 actionable variants in indels of cfDNA. Analysis of tier 1+2 actionable variants in indels of cfDNA per patient.

Patient (cfDNA)	Location	Allele	Consequence	Impact	Symbol	Biotype	Exon	Intron	Protein position
1	11:125505377-125505377	A	Frameshift variant	HIGH	CHEK1	Protein coding	6/11	-	223
	11:125505377-125505377	A	Frameshift variant	HIGH	CHEK1	Protein coding	6/12	-	239
	11:125505377-125505377	A	Frameshift variant	HIGH	CHEK1	Protein coding	7/14	-	223
	11:125505377-125505377	A	Frameshift variant	HIGH	CHEK1	Protein coding	7/13	-	223
	11:125505377-125505377	A	Frameshift variant	HIGH	CHEK1	Protein coding	6/13	-	223
	11:125505377-125505377	A	Frameshift variant	HIGH	CHEK1	Protein coding	5/6	-	144
	11:125505377-125505377	A	Frameshift variant	HIGH	CHEK1	Protein coding	7/12	-	223
3	-	-	-	-	-	-	-	-	-
4	11:125505377-125505377	A	Frameshift variant	HIGH	CHEK1	Protein coding	6/11	-	223
	11:125505377-125505377	A	Frameshift variant	HIGH	CHEK1	Protein coding	6/12	-	239
	11:125505377-125505377	A	Frameshift variant	HIGH	CHEK1	Protein coding	7/14	-	223
	11:125505377-125505377	A	Frameshift variant	HIGH	CHEK1	Protein coding	7/13	-	223
	11:125505377-125505377	A	Frameshift variant	HIGH	CHEK1	Protein coding	6/13	-	223
	11:125505377-125505377	A	Frameshift variant	HIGH	CHEK1	Protein coding	5/6	-	144
	11:125505377-125505377	A	Frameshift variant	HIGH	CHEK1	Protein coding	7/12	-	223
5	11:125505377-125505377	A	Frameshift variant	HIGH	CHEK1	Protein coding	6/11	-	223
	11:125505377-125505377	A	Frameshift variant	HIGH	CHEK1	Protein coding	6/12	-	239
	11:125505377-125505377	A	Frameshift variant	HIGH	CHEK1	Protein coding	7/14	-	223
	11:125505377-125505377	A	Frameshift variant	HIGH	CHEK1	Protein coding	7/13	-	223
	11:125505377-125505377	A	Frameshift variant	HIGH	CHEK1	Protein coding	6/13	-	223
	11:125505377-125505377	A	Frameshift variant	HIGH	CHEK1	Protein coding	5/6	-	144
	11:125505377-125505377	A	Frameshift variant	HIGH	CHEK1	Protein coding	7/12	-	223
6	11:125505377-125505377	A	Frameshift variant	HIGH	CHEK1	Protein coding	6/11	-	223
	11:125505377-125505377	A	Frameshift variant	HIGH	CHEK1	Protein coding	6/12	-	239
	11:125505377-125505377	A	Frameshift variant	HIGH	CHEK1	Protein coding	7/14	-	223

	11:125505377-125505377	A	Frameshift variant	HIGH	CHEK1	Protein coding	7/13	-	223
	11:125505377-125505377	A	Frameshift variant	HIGH	CHEK1	Protein coding	6/13	-	223
	11:125505377-125505377	A	Frameshift variant	HIGH	CHEK1	Protein coding	5/6	-	144
	11:125505377-125505377	A	Frameshift variant	HIGH	CHEK1	Protein coding	7/13	-	223
	11:125505377-125505377	A	Frameshift variant	HIGH	CHEK1	Protein coding	7/12	-	223
	8:38285913-38285916	-	Inframe deletion	MODERATE	FGFR1	Protein coding	3/17	-	43-44
	8:38285913-38285916	-	Inframe deletion	MODERATE	FGFR1	Protein coding	5/19	-	124-125
	8:38285913-38285916	-	Inframe deletion	MODERATE	FGFR1	Protein coding	4/18	-	135-136
	8:38285913-38285916	-	Inframe deletion	MODERATE	FGFR1	Protein coding	4/18	-	132-133
	8:38285913-38285916	-	Inframe deletion	MODERATE	FGFR1	Protein coding	2/16	-	43-44
	8:38285913-38285916	-	Inframe deletion	MODERATE	FGFR1	Protein coding	5/19	-	132-133
	8:38285913-38285916	-	Inframe deletion	MODERATE	FGFR1	Protein coding	4/18	-	132-133
	8:38285913-38285916	-	Inframe deletion	MODERATE	FGFR1	Protein coding	5/19	-	165-166
	8:38285913-38285916	-	Inframe deletion	MODERATE	FGFR1	Protein coding	4/4	-	43-44
	8:38285913-38285916	-	Inframe deletion	MODERATE	FGFR1	Protein coding	4/7	-	132-133
	8:38285913-38285916	-	Inframe deletion	MODERATE	FGFR1	Protein coding	3/5	-	43-44
	8:38285913-38285916	-	Inframe deletion	MODERATE	FGFR1	Protein coding	3/4	-	43-44
7	9:133759489-133759492	-	Inframe deletion	MODERATE	ABL1	Protein coding	11/11	-	605
	9:133759489-133759492	-	Inframe deletion	MODERATE	ABL1	Protein coding	11/11	-	624
8	11:125505377-125505377	A	Frameshift variant	HIGH	CHEK1	Protein coding	6/11	-	223
	11:125505377-125505377	A	Frameshift variant	HIGH	CHEK1	Protein coding	6/12	-	239
	11:125505377-125505377	A	Frameshift variant	HIGH	CHEK1	Protein coding	7/14	-	223
	11:125505377-125505377	A	Frameshift variant	HIGH	CHEK1	Protein coding	7/13	-	223
	11:125505377-125505377	A	Frameshift variant	HIGH	CHEK1	Protein coding	6/13	-	223
	11:125505377-125505377	A	Frameshift variant	HIGH	CHEK1	Protein coding	5/6	-	144
	11:125505377-125505377	A	Frameshift variant	HIGH	CHEK1	Protein coding	7/12	-	223

	9:133759489-133759492	A	Inframe deletion	MODERATE	ABL1	Protein coding	11/11	-	605
	9:133759489-133759492	A	Inframe deletion	MODERATE	ABL1	Protein coding	11/11	-	624
9	8:38285913-38285916	-	Inframe deletion	MODERATE	FGFR1	Protein coding	3/17	-	43-44
	8:38285913-38285916	-	Inframe deletion	MODERATE	FGFR1	Protein coding	5/19	-	124-125
	8:38285913-38285916	-	Inframe deletion	MODERATE	FGFR1	Protein coding	4/18	-	135-136
	8:38285913-38285916	-	Inframe deletion	MODERATE	FGFR1	Protein coding	4/18	-	132-133
	8:38285913-38285916	-	Inframe deletion	MODERATE	FGFR1	Protein coding	2/16	-	43-44
	8:38285913-38285916	-	Inframe deletion	MODERATE	FGFR1	Protein coding	5/19	-	132-133
	8:38285913-38285916	-	Inframe deletion	MODERATE	FGFR1	Protein coding	5/19	-	165-166
	8:38285913-38285916	-	Inframe deletion	MODERATE	FGFR1	Protein coding	4/4	-	43-44
	8:38285913-38285916	-	Inframe deletion	MODERATE	FGFR1	Protein coding	4/7	-	132-133
	8:38285913-38285916	-	Inframe deletion	MODERATE	FGFR1	Protein coding	3/5	-	43-44
	8:38285913-38285916	-	Inframe deletion	MODERATE	FGFR1	Protein coding	3/4	-	43-44
	8:38285913-38285916	-	Inframe deletion	MODERATE	FGFR1	Protein coding	4/18	-	132-133
	9:133759489-133759492	-	Inframe deletion	MODERATE	ABL1	Protein coding	11/11	-	605
	9:133759489-133759492	-	Inframe deletion	MODERATE	ABL1	Protein coding	11/11	-	624
10	11:125505377-125505377	A	Frameshift variant	HIGH	CHEK1	Protein coding	6/11	-	223
	11:125505377-125505377	A	Frameshift variant	HIGH	CHEK1	Protein coding	6/12	-	239
	11:125505377-125505377	A	Frameshift variant	HIGH	CHEK1	Protein coding	7/14	-	223
	11:125505377-125505377	A	Frameshift variant	HIGH	CHEK1	Protein coding	7/13	-	223
	11:125505377-125505377	A	Frameshift variant	HIGH	CHEK1	Protein coding	6/13	-	223
	11:125505377-125505377	A	Frameshift variant	HIGH	CHEK1	Protein coding	5/6	-	144
	11:125505377-125505377	A	Frameshift variant	HIGH	CHEK1	Protein coding	7/12	-	223
	14:68331715-68331719	-	Splice acceptor variant, intron variant	HIGH	RAD51 B	Protein coding	-	3/9	-
	14:68331715-68331719	-	Splice acceptor variant, intron variant	HIGH	RAD51 B	Protein coding	-	4/10	-

14:68331715-68331719	-	Splice acceptor variant, intron variant, NMD transcript variant	HIGH	RAD51B	Nonsense mediated decay	-	5/11	-
14:68331715-68331719	-	Splice acceptor variant, intron variant	HIGH	RAD51B	Protein coding	-	4/4	-
14:68331715-68331719	-	Splice acceptor variant, intron variant	HIGH	RAD51B	Protein coding	-	4/11	-
14:68331715-68331719	-	Splice acceptor variant, intron variant, non-coding transcript variant	HIGH	RAD51B	Processed transcript	-	1/3	-

Agreements between cf- and tumorDNA are marked in red

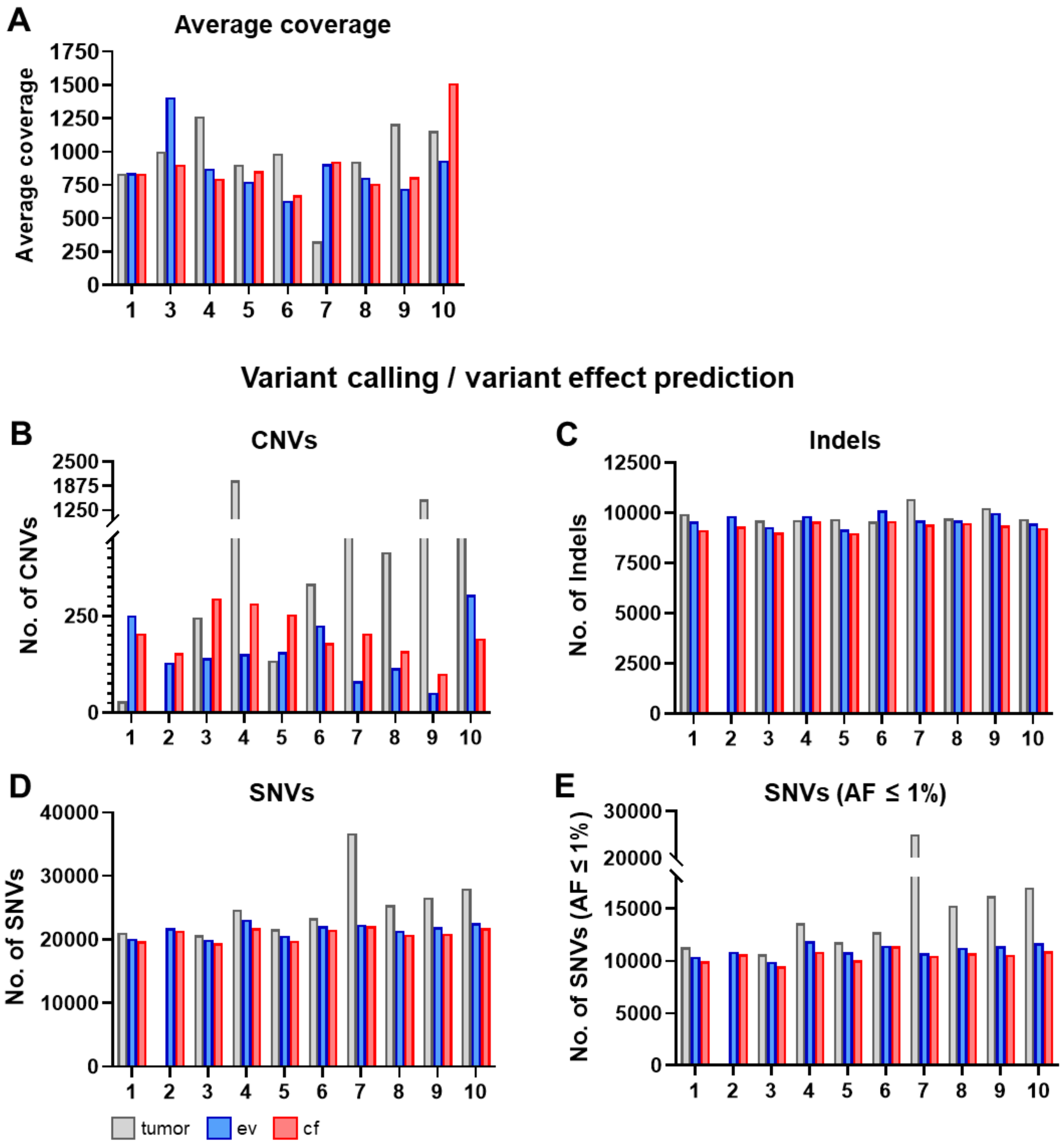

Supplemental Figure S1: Coverage and variant calling. (A) Average coverage per patient. Number of called variants of (B) CNVs (by CeGaT) and of detected variants (VEP, Ensembl) of (C) Indels; (D) unfiltered SNVs as well as (E) filtered SNVs (AF ≤1%)perpatient.

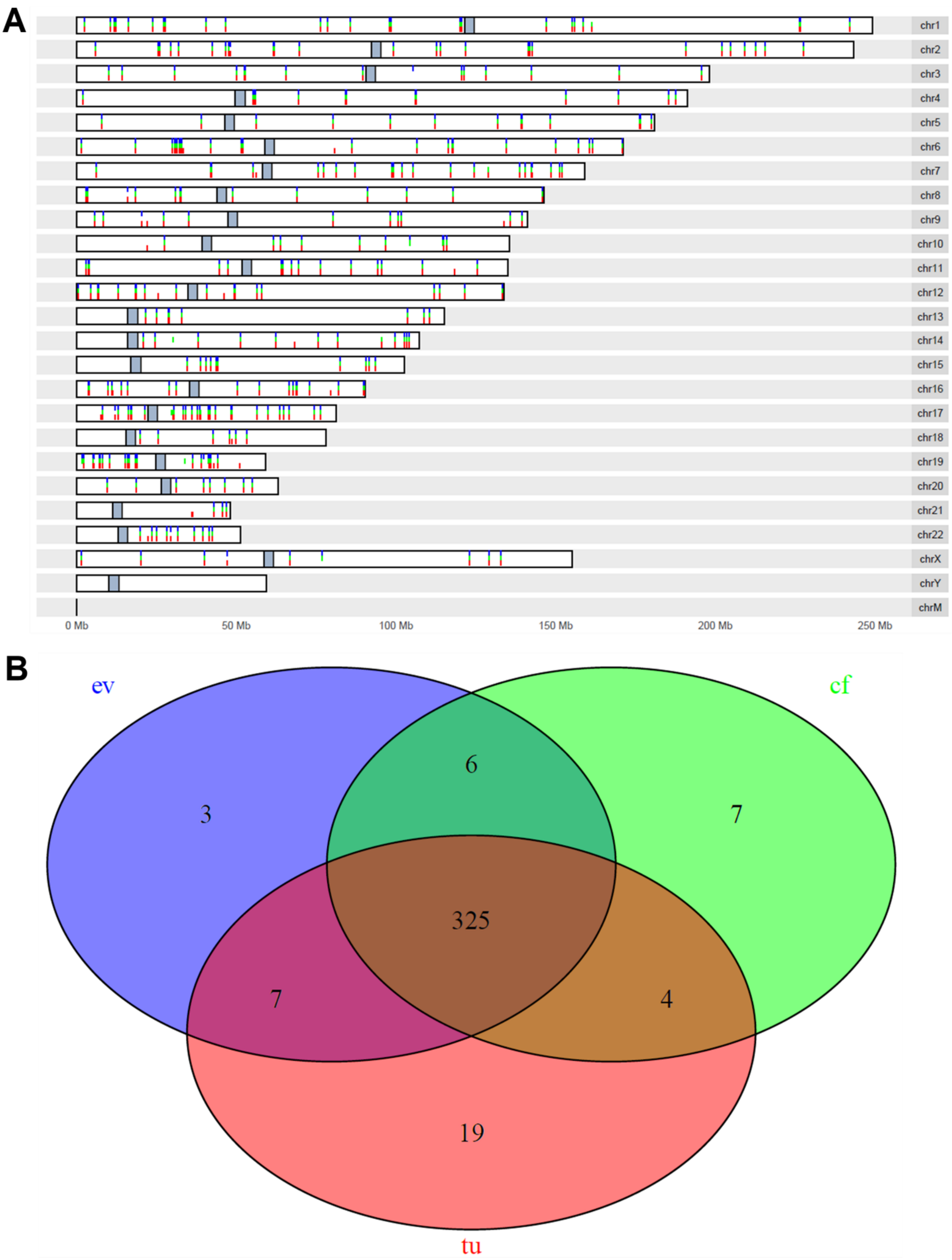

Supplemental Figure S2: Chromosomal positions of SNVs for patient 1. (A) Ideogram showing chromosomal locations of SNV variants and (B) Venn diagram indicating overlapping chromosomal positions for tumor- (red), ev- (blue) and cfDNA (green).

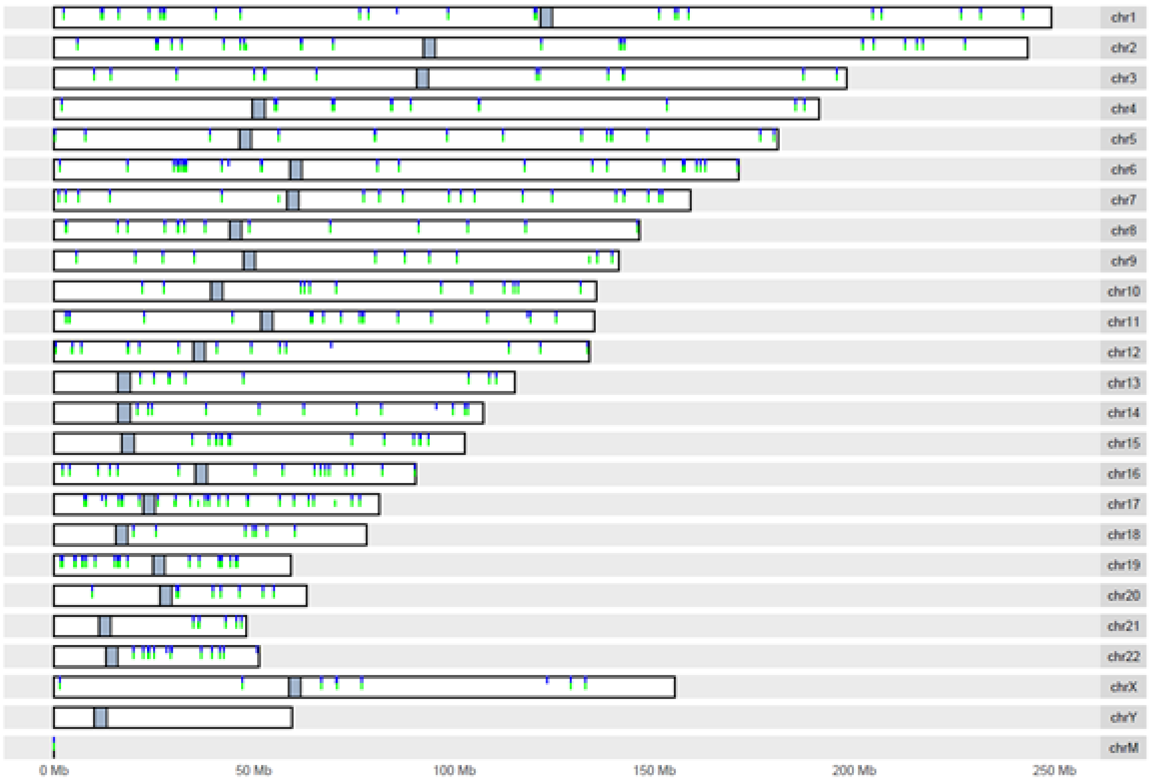

Supplemental Figure S3: Chromosomal positions of SNVs for patient 2. Ideogram showing chromosomal locations of SNV variants for ev- (blue) and cfDNA (green).

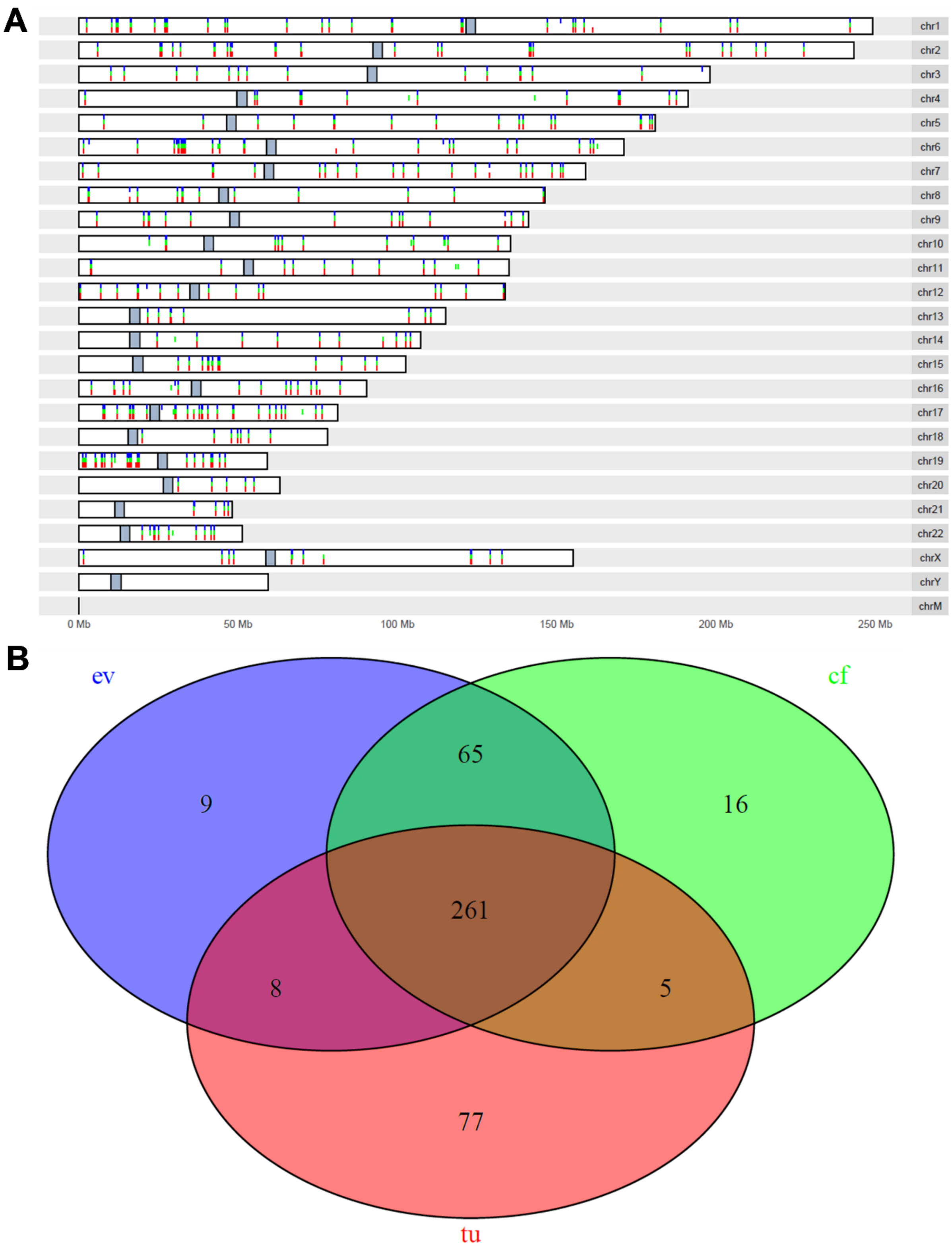

Supplemental Figure S4: Chromosomal positions of SNVs for patient 3. (A) Ideogram showing chromosomal locations of SNV variants and (B) Venn diagram indicating overlapping chromosomal positions for tumor- (red), ev- (blue) and cfDNA (green).

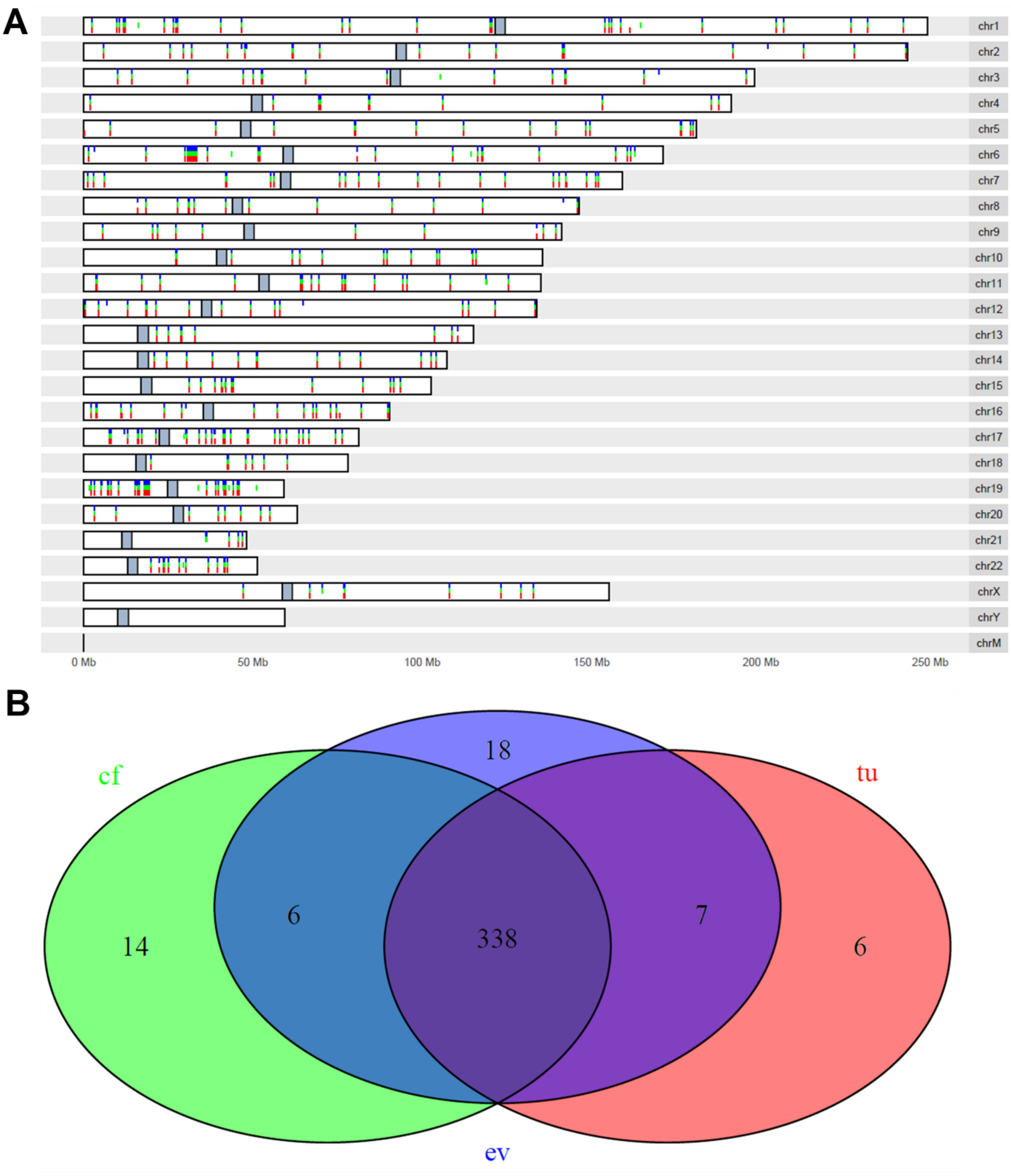

Supplemental Figure S5: Chromosomal positions of SNVs for patient 4. (A) Ideogram showing chromosomal locations of SNV variants and (B) Venn diagram indicating overlapping chromosomal positions for tumor- (red), ev- (blue) and cfDNA (green).

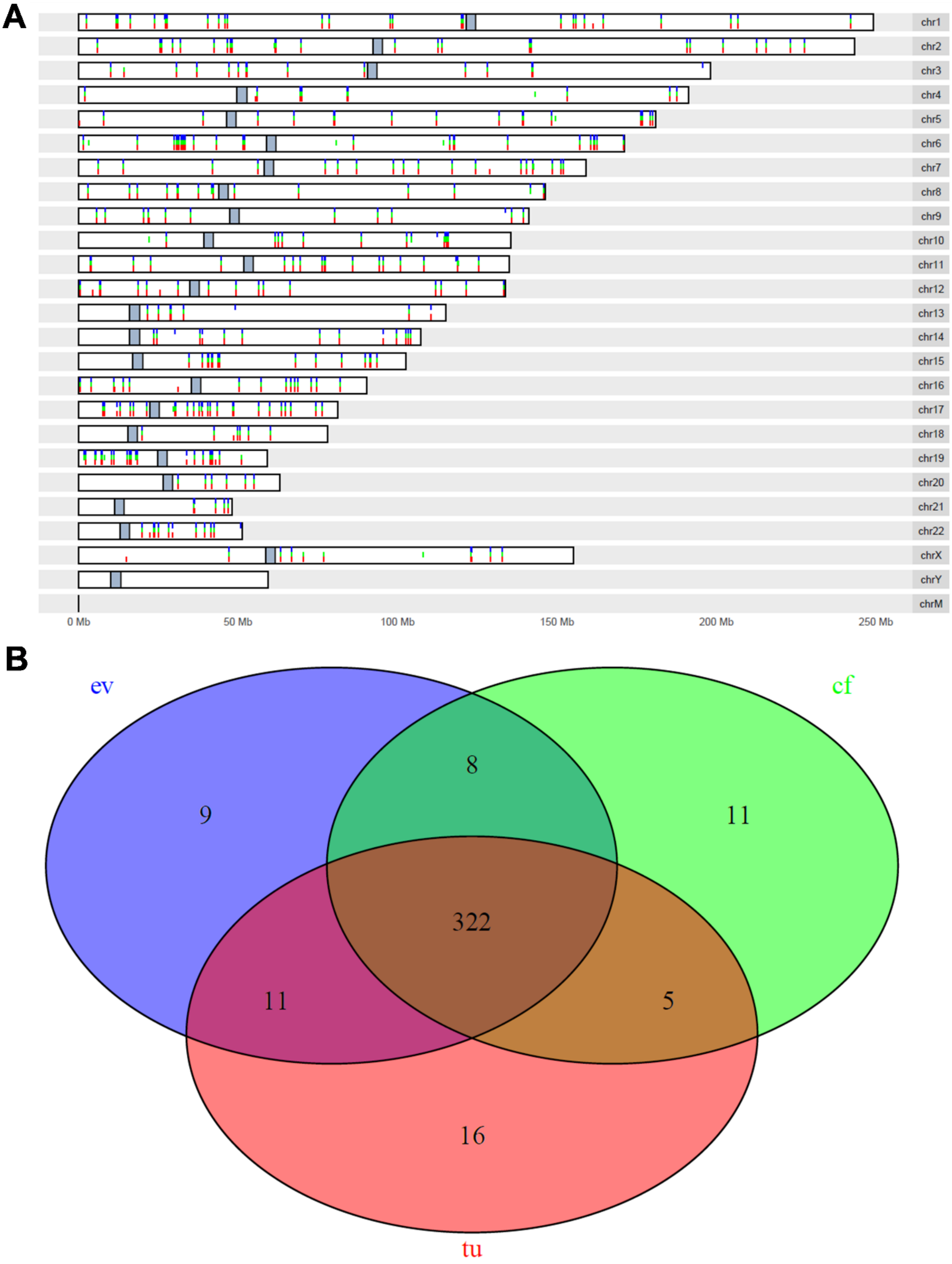

Supplemental Figure S6: Chromosomal positions of SNVs for patient 5. (A) Ideogram showing chromosomal locations of SNV variants and (B) Venn diagram indicating overlapping chromosomal positions for tumor- (red), ev- (blue) and cfDNA (green).

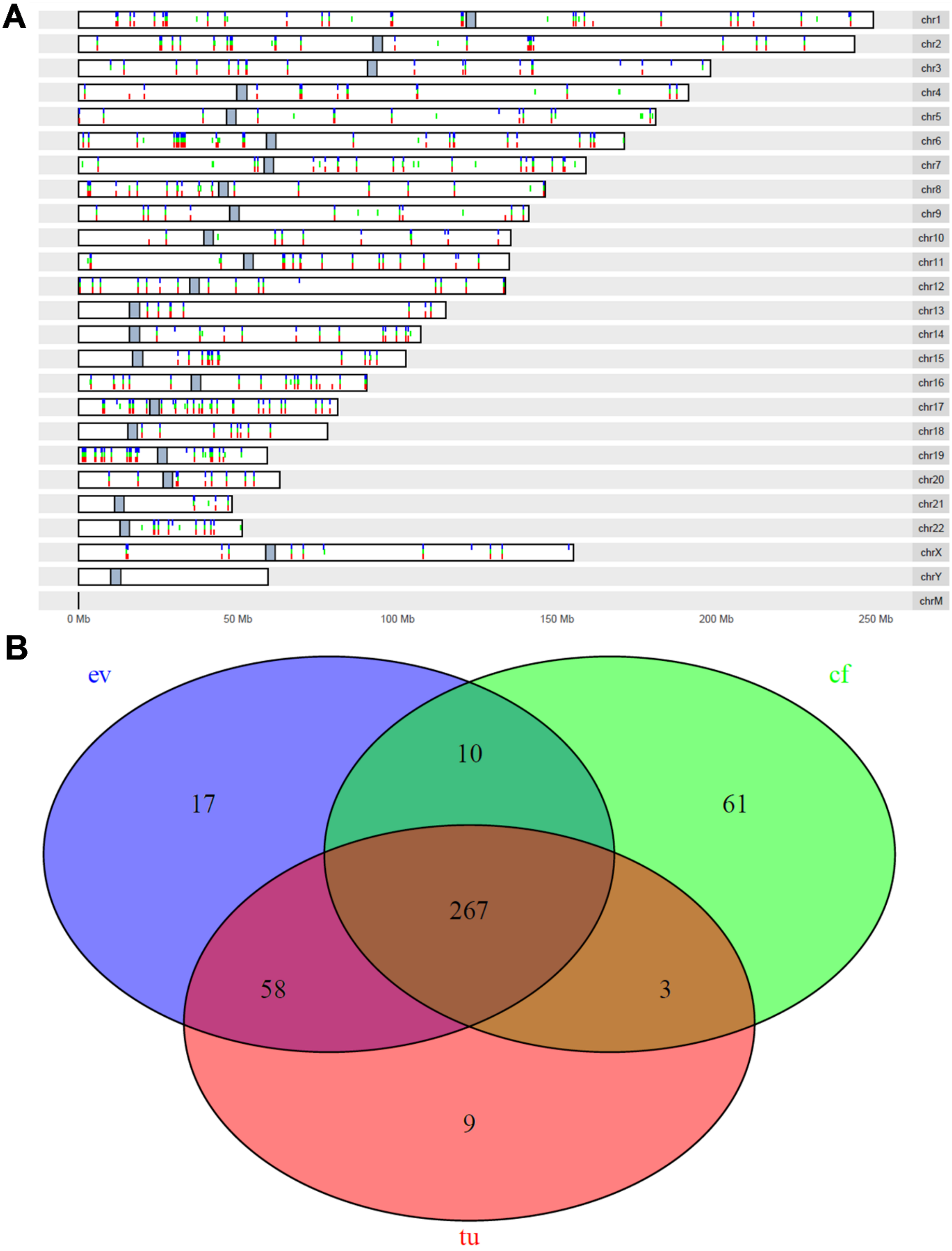

Supplemental Figure S7: Chromosomal positions of SNVs for patient 6. (A) Ideogram showing chromosomal locations of SNV variants and (B) Venn diagram indicating overlapping chromosomal positions for tumor- (red), ev- (blue) and cfDNA (green).

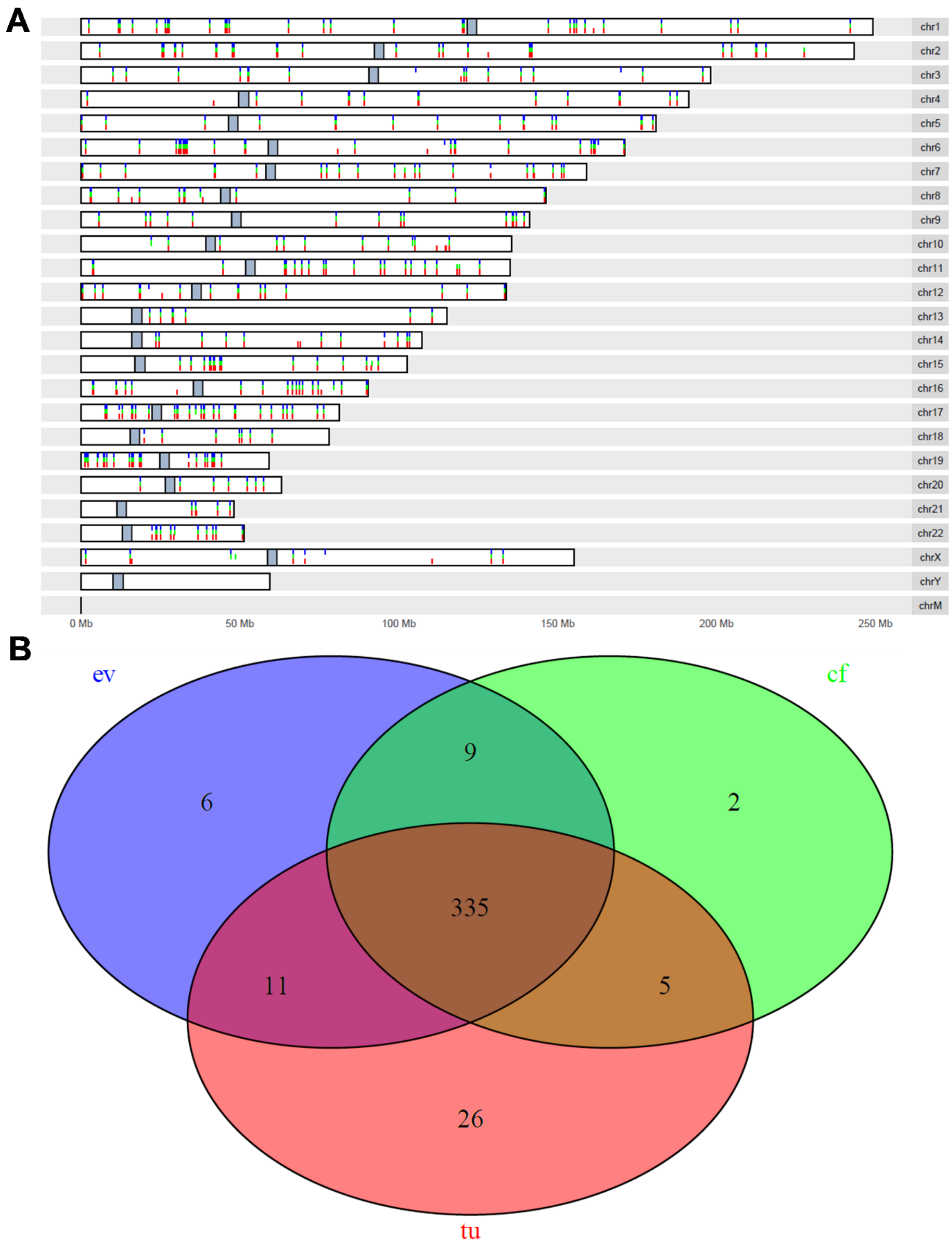

Supplemental Figure S8: Chromosomal positions of SNVs for patient 7. (A) Ideogram showing chromosomal locations of SNV variants and (B) Venn diagram indicating overlapping chromosomal positions for tumor- (red), ev- (blue) and cfDNA (green).

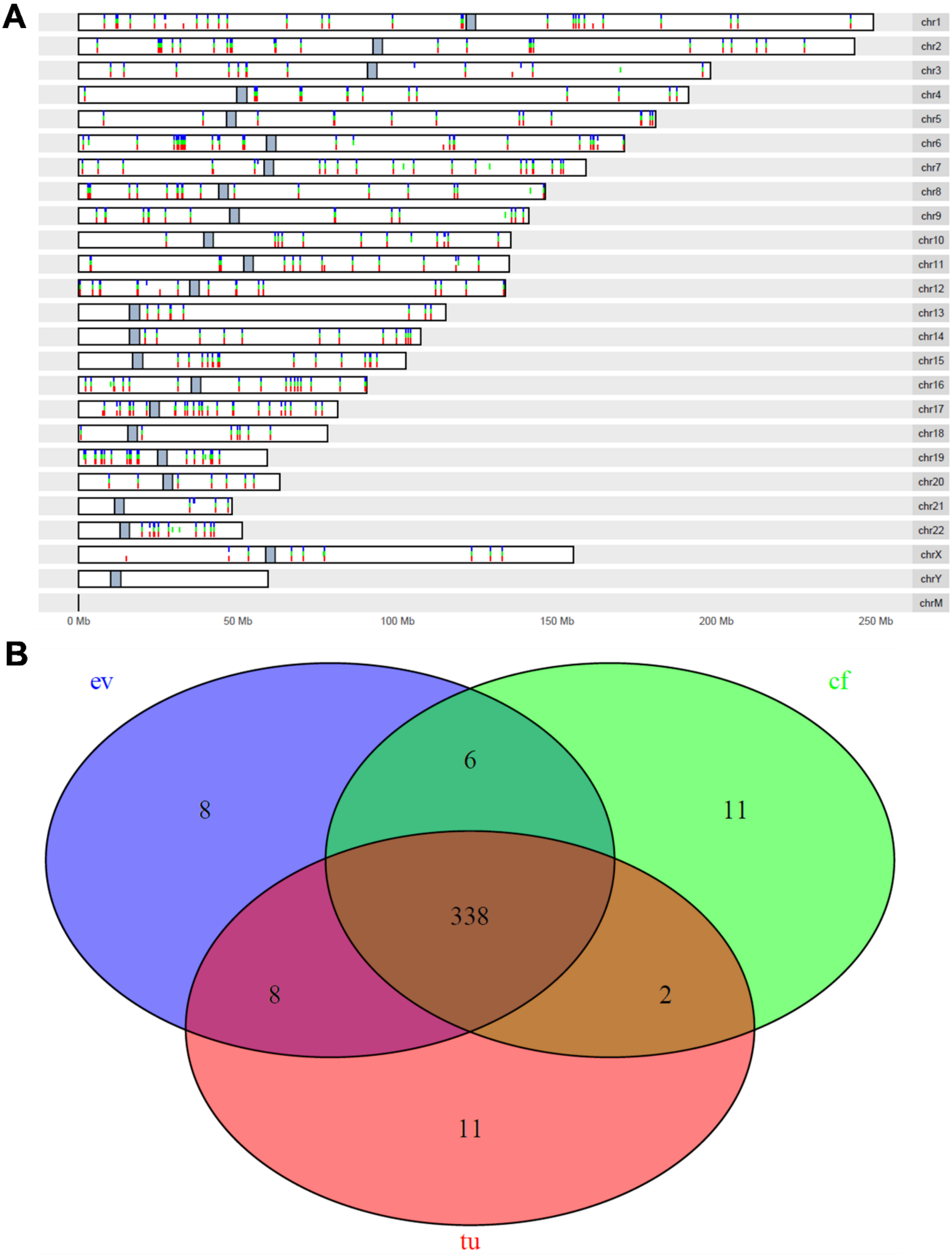

Supplemental Figure S9: Chromosomal positions of SNVs for patient 8. (A) Ideogram showing chromosomal locations of SNV variants and (B) Venn diagram indicating overlapping chromosomal positions for tumor- (red), ev- (blue) and cfDNA (green).

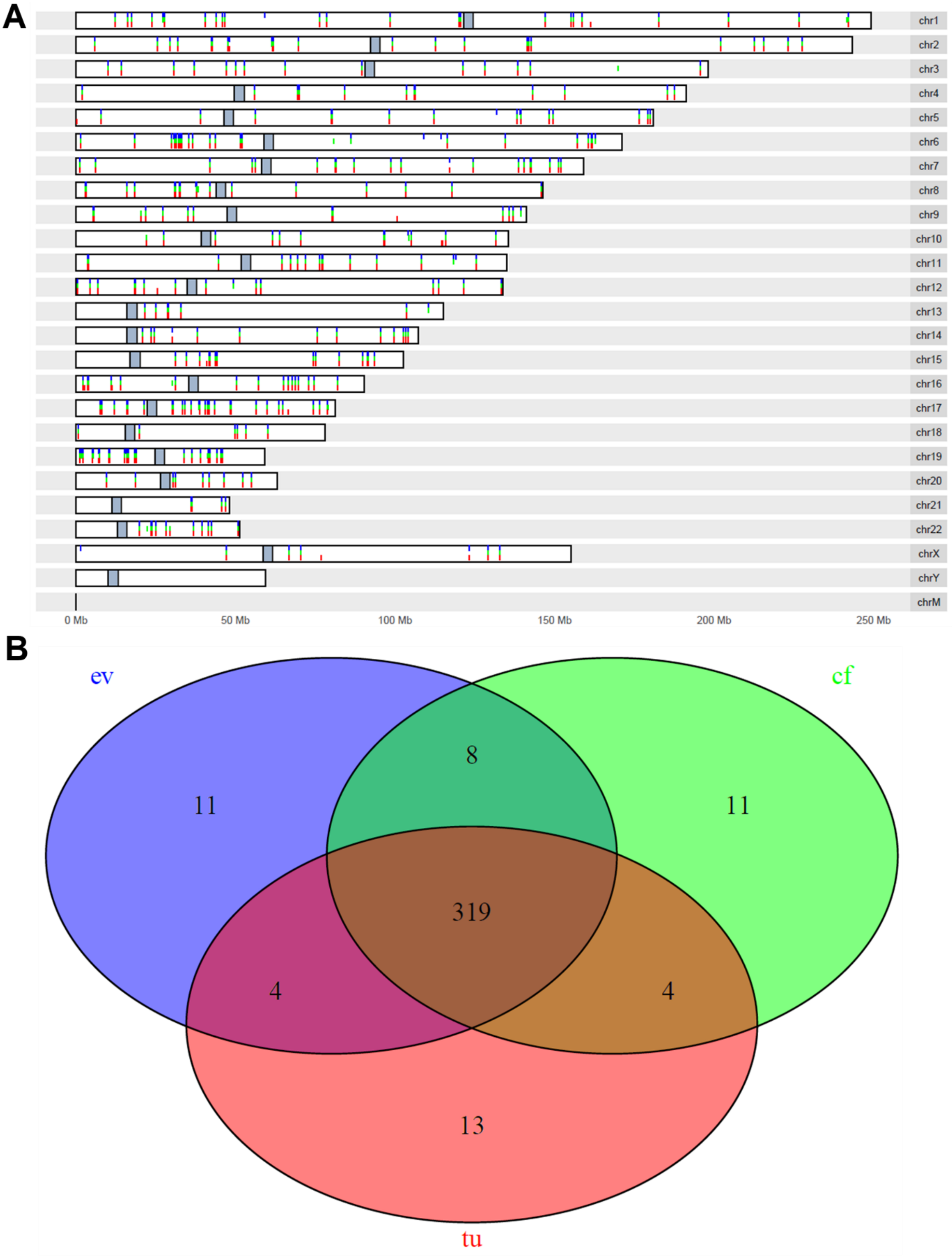

Supplemental Figure S10: Chromosomal positions of SNVs for patient 9. (A) Ideogram showing chromosomal locations of SNV variants and (B) Venn diagram indicating overlapping chromosomal positions for tumor- (red), ev- (blue) and cfDNA (green).

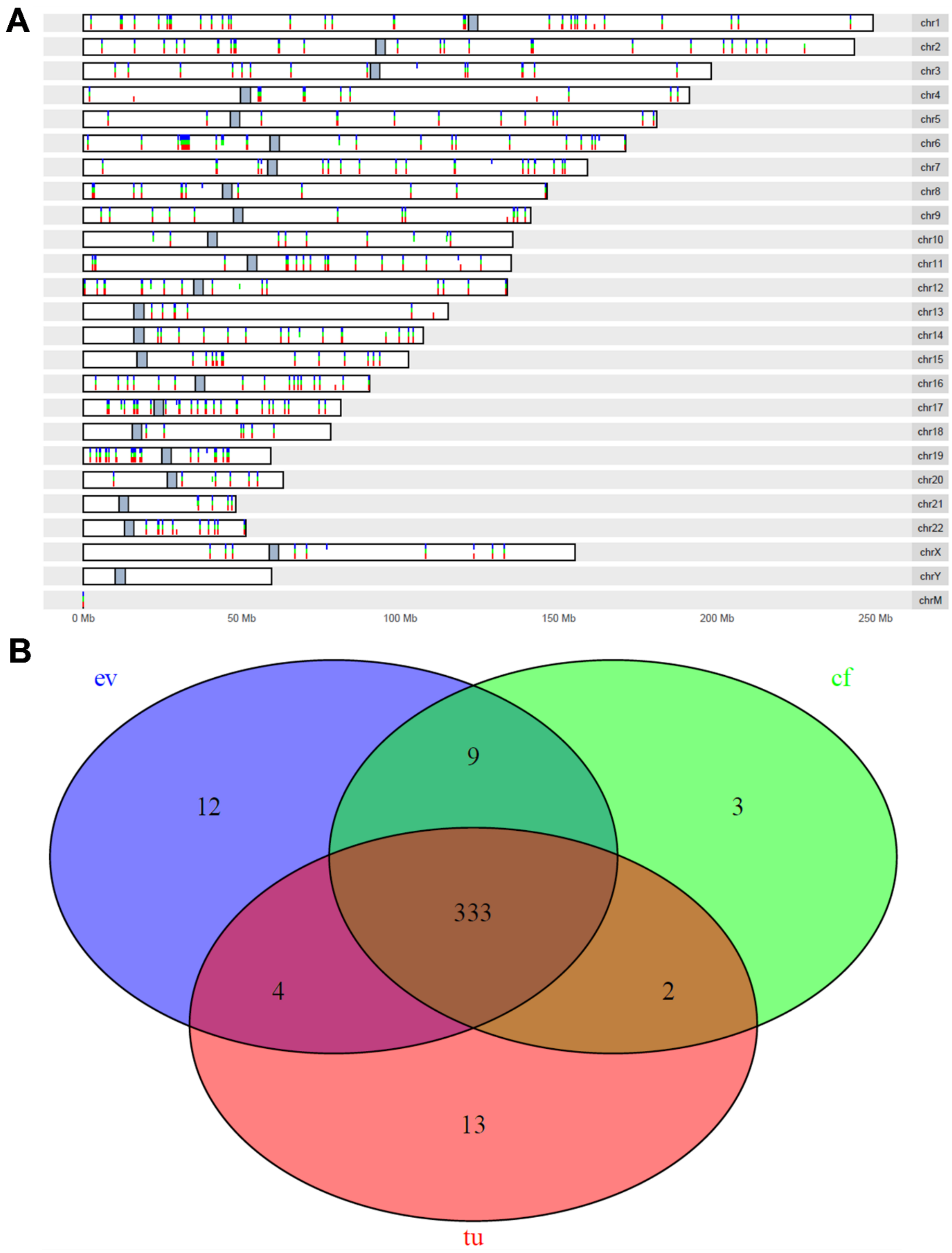

Supplemental Figure S11: Chromosomal positions of SNVs for patient 10. (A) Ideogram showing chromosomal locations of SNV variants and (B) Venn diagram indicating overlapping chromosomal positions for tumor- (red), ev- (blue) and cfDNA (green).

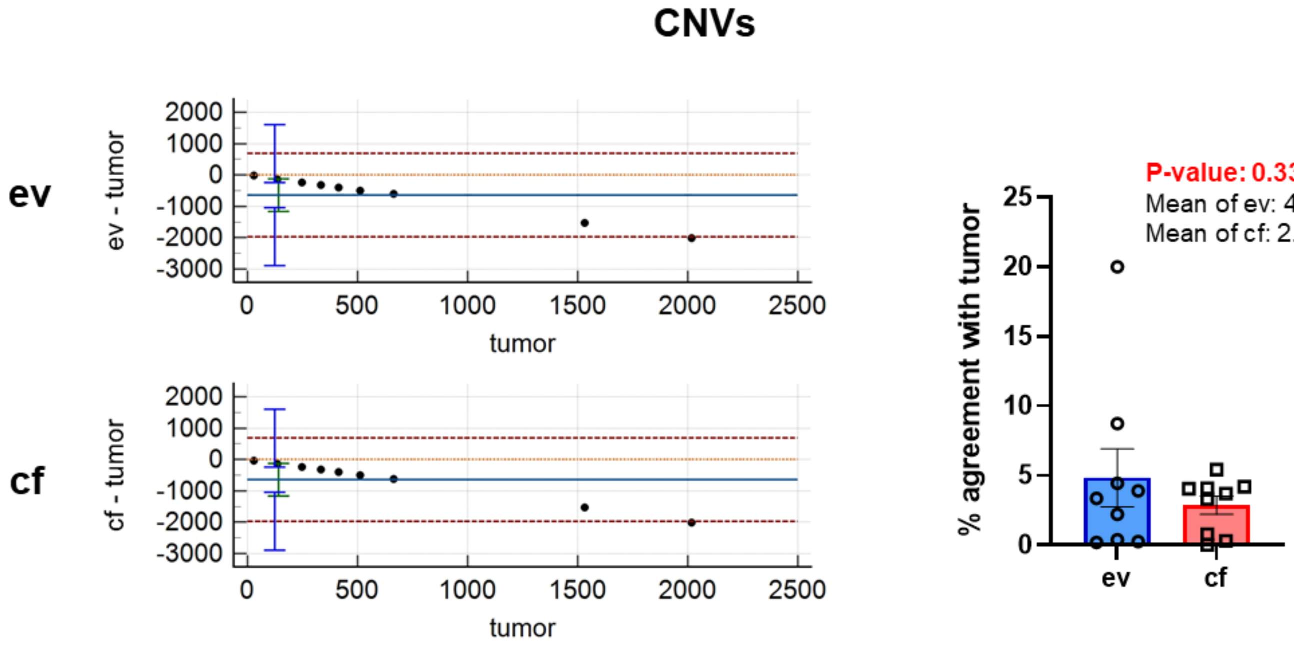

Supplemental Figure S12: Agreement of CNVs between ev- and tumor as well as cf- and tumorDNA. Bland-Altman plots and agreement (%) of variants detected in CNVs between tumor- and evDNA as well as tumor- and cfDNA. Statistical tests: (A) Two-tailed paired Student t-test.

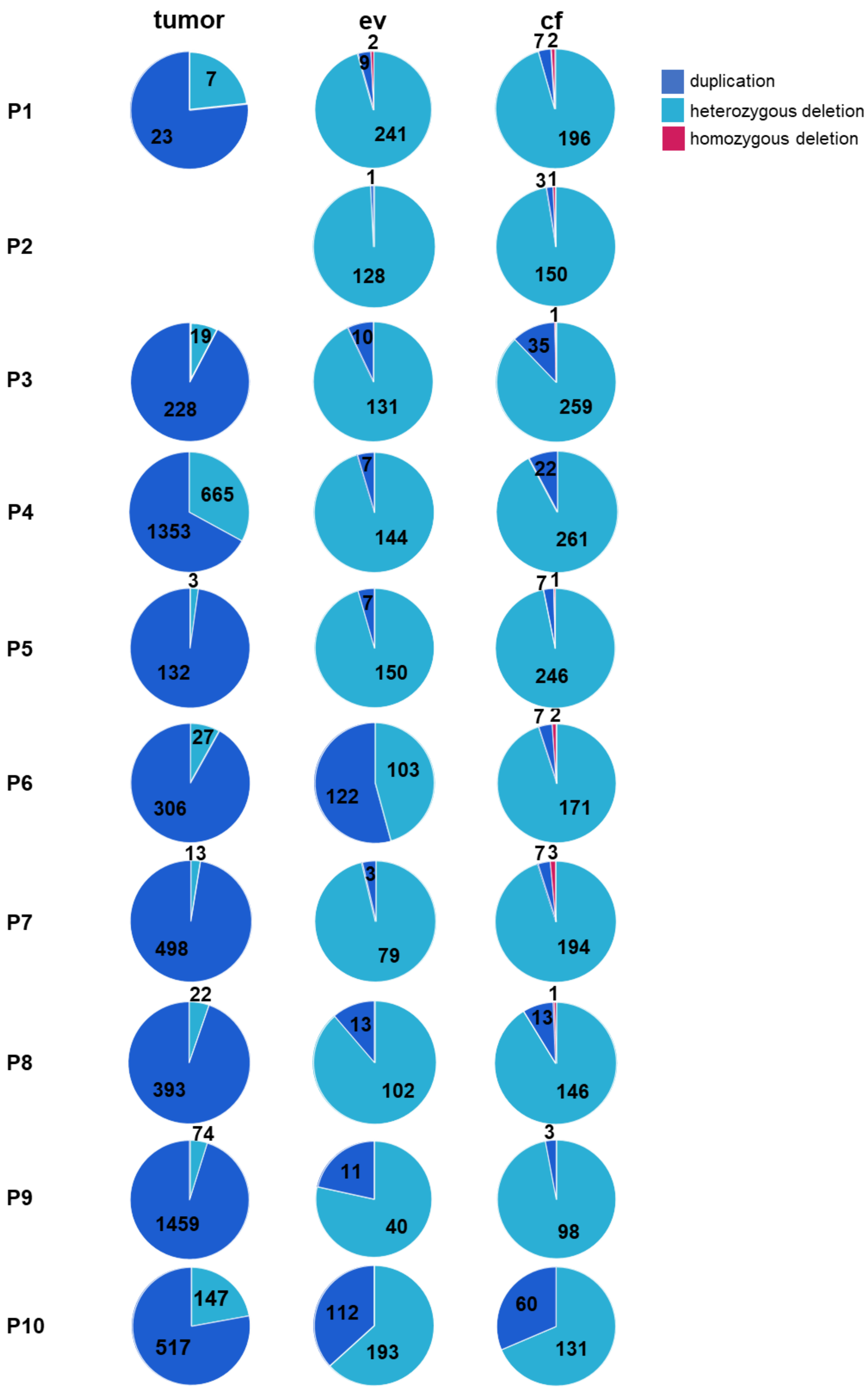

Supplemental Figure S13: Overview of detected CNVs. Individual analysis of CNVs in tumor-, ev- and cfDNA for all patients.

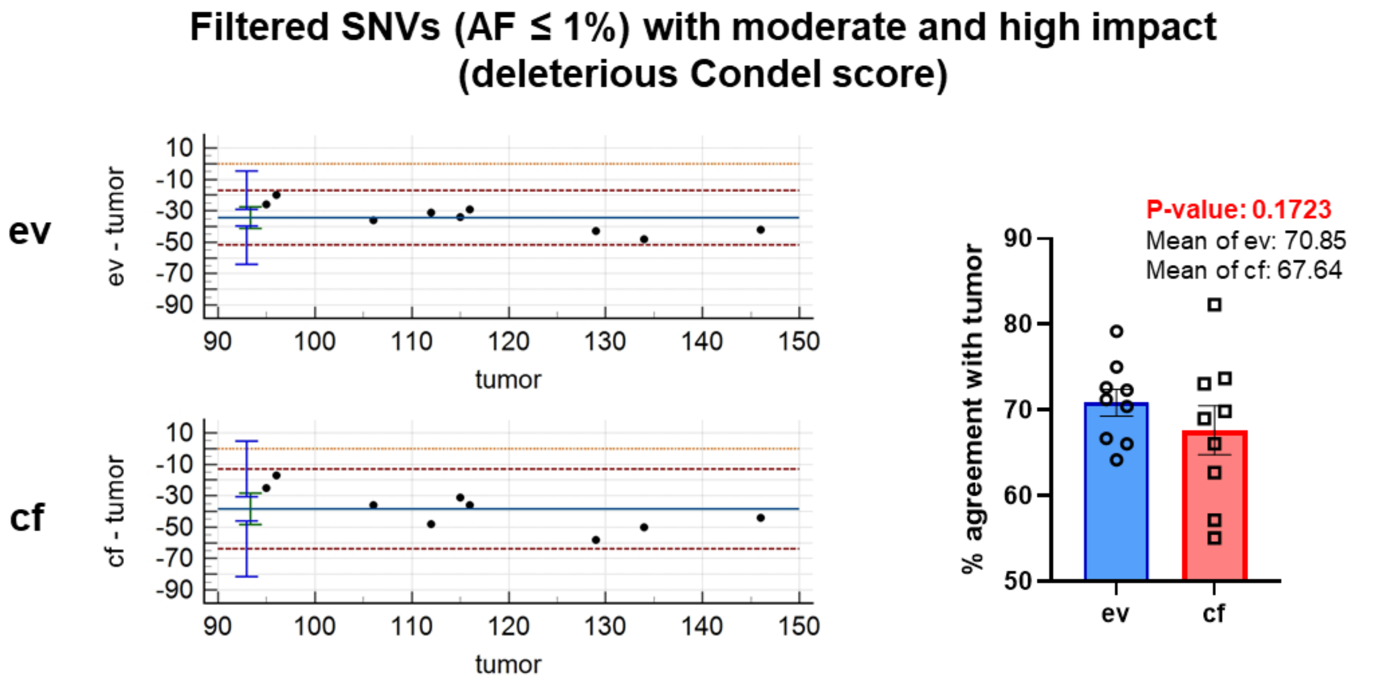

Supplemental Figure S14: Agreement of SNVs between ev- and tumor as well as cf- and tumorDNA. Bland-Altman plots and agreement (%) of variants detected in filtered SNVs (AF≤1%) with moderate and high impact and a deleterious Condel score between tumor- and evDNA as well as tumor- and cfDNA. Statistical tests: (A) Two-tailed paired Student t-test.

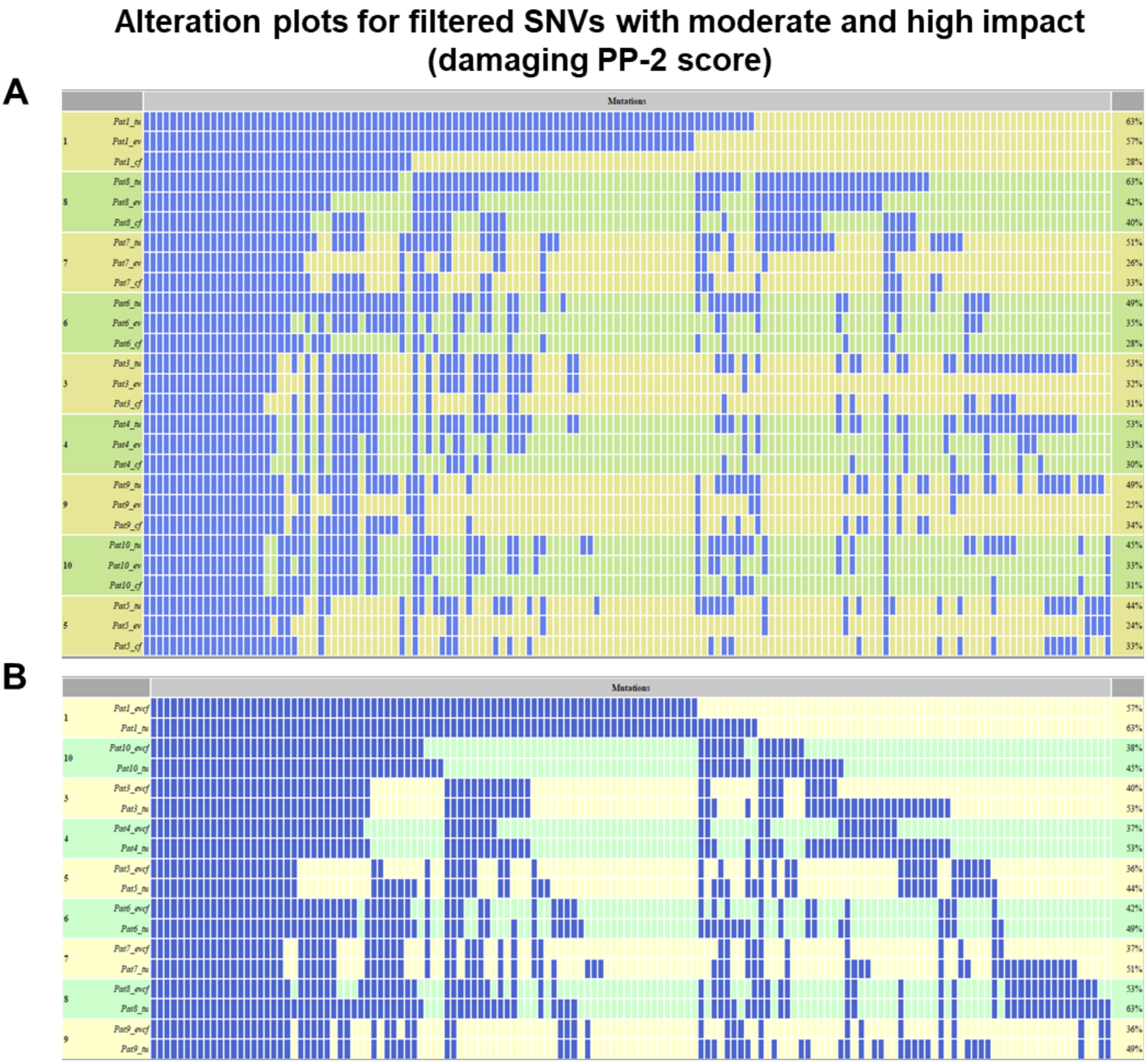

Supplemental Figure S15: Representative alteration plots for v ariants detected in tumor- and ev- or cfDNA. Blue bars indicate SNVs present in the corresponding sample indicated at the left side as tu/ev/cf. SNVs were preselected for impact score mod-erate/high and PP-2 score damaging (n=144). (A) Alteration plot for SNVs of tumor-, ev- and cfDNA. (B) Alteration plot for SNVs of tumorDNA and a combination of ev- and cfDNA.

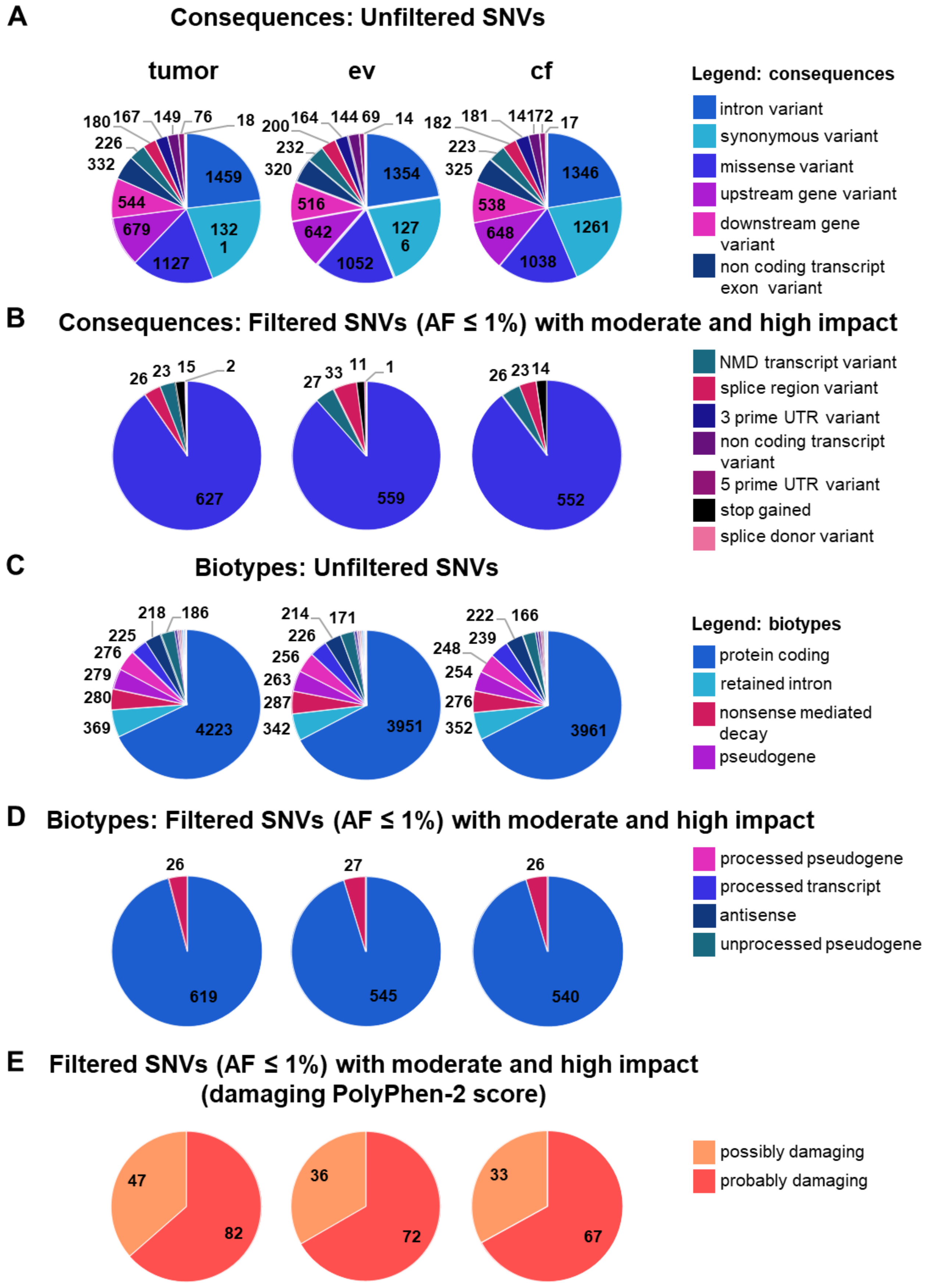

Supplemental Figure S16: Analysis of SNVs for patient 1. (A) Consequences of unfiltered SNVs and (B) filtered SNVs (AF≤1%) with moderate and high impact, (C) biotypes of unfiltered and (D) filtered SNVs (AF≤1%) with moderate and high impact as well as (D) number of variants with a possibly or probably damaging PP-2 score of filtered SNVs (AF≤1%) with moderate and high impact found in tumor-, ev- and cfDNA.

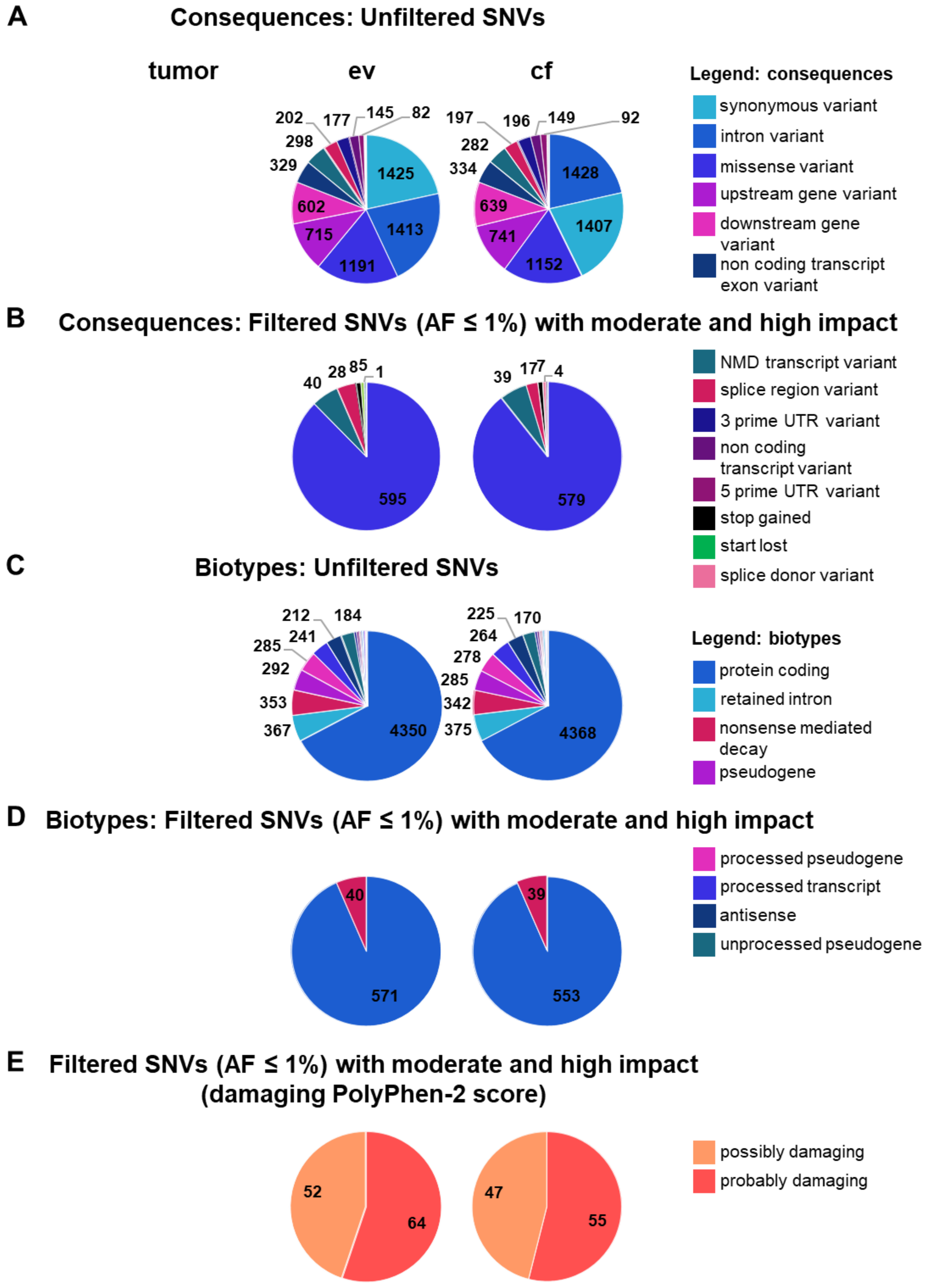

Supplemental Figure S17: Analysis of SNVs for patient 2. (A) Consequences of unfiltered SNVs and (B) filtered SNVs (AF≤1%) with moderate and high impact, (C) biotypes of unfiltered and (D) filtered SNVs (AF≤1%) with moderate and high impact as well as (D) number of variants with a possibly or probably damaging PP-2 score of filtered SNVs (AF≤1%) with moderate and high impact found in tumor-, ev- and cfDNA.

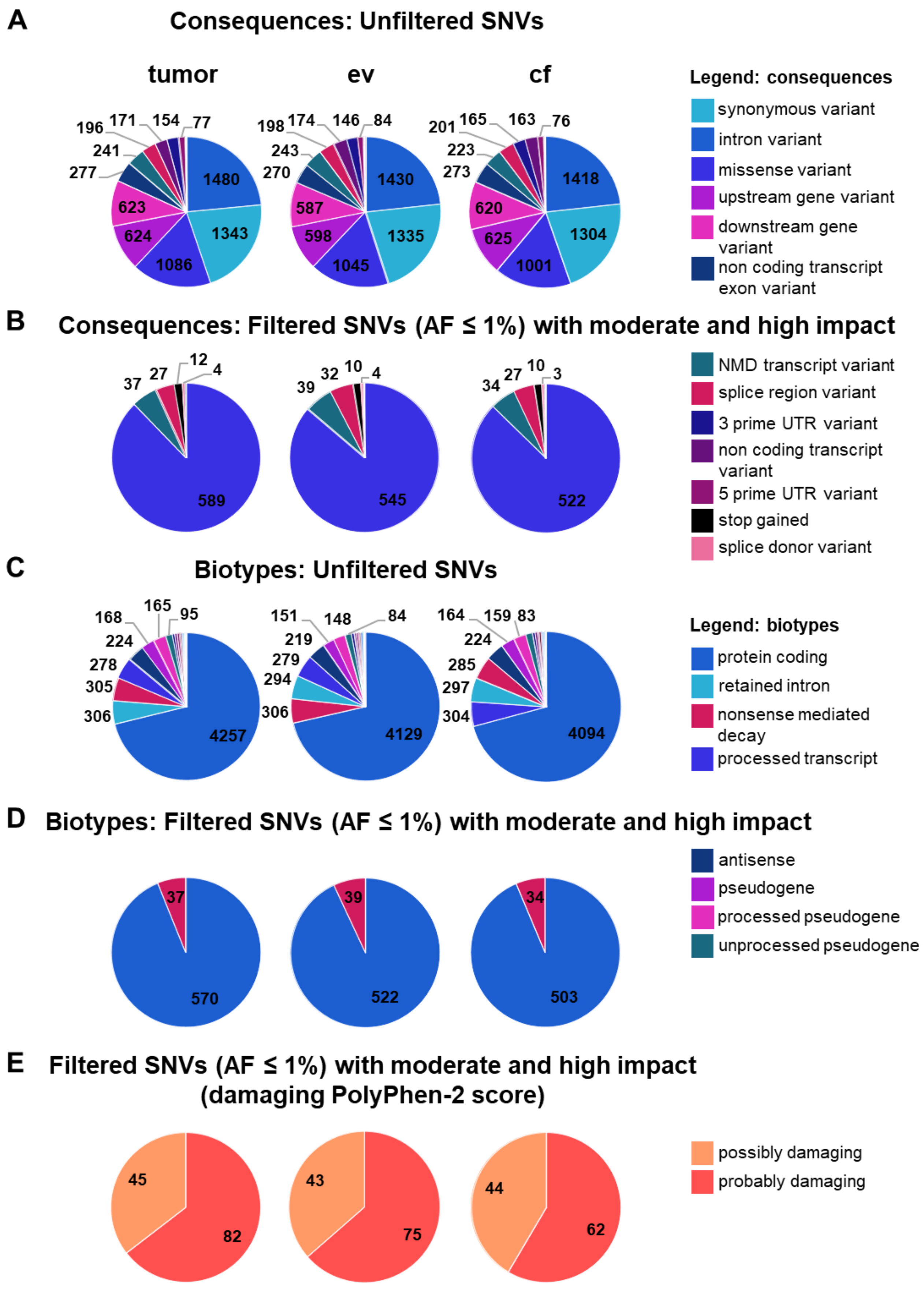

Supplemental Figure S18: Analysis of SNVs for patient 3. (A) Consequences of unfiltered SNVs and (B) filtered SNVs (AF≤1%) with moderate and high impact, (C) biotypes of unfiltered and (D) filtered SNVs (AF≤1%) with moderate and high impact as well as (D) number of variants with a possibly or probably damaging PP-2 score of filtered SNVs (AF≤1%) with moderate and high impact found in tumor-, ev- and cfDNA.

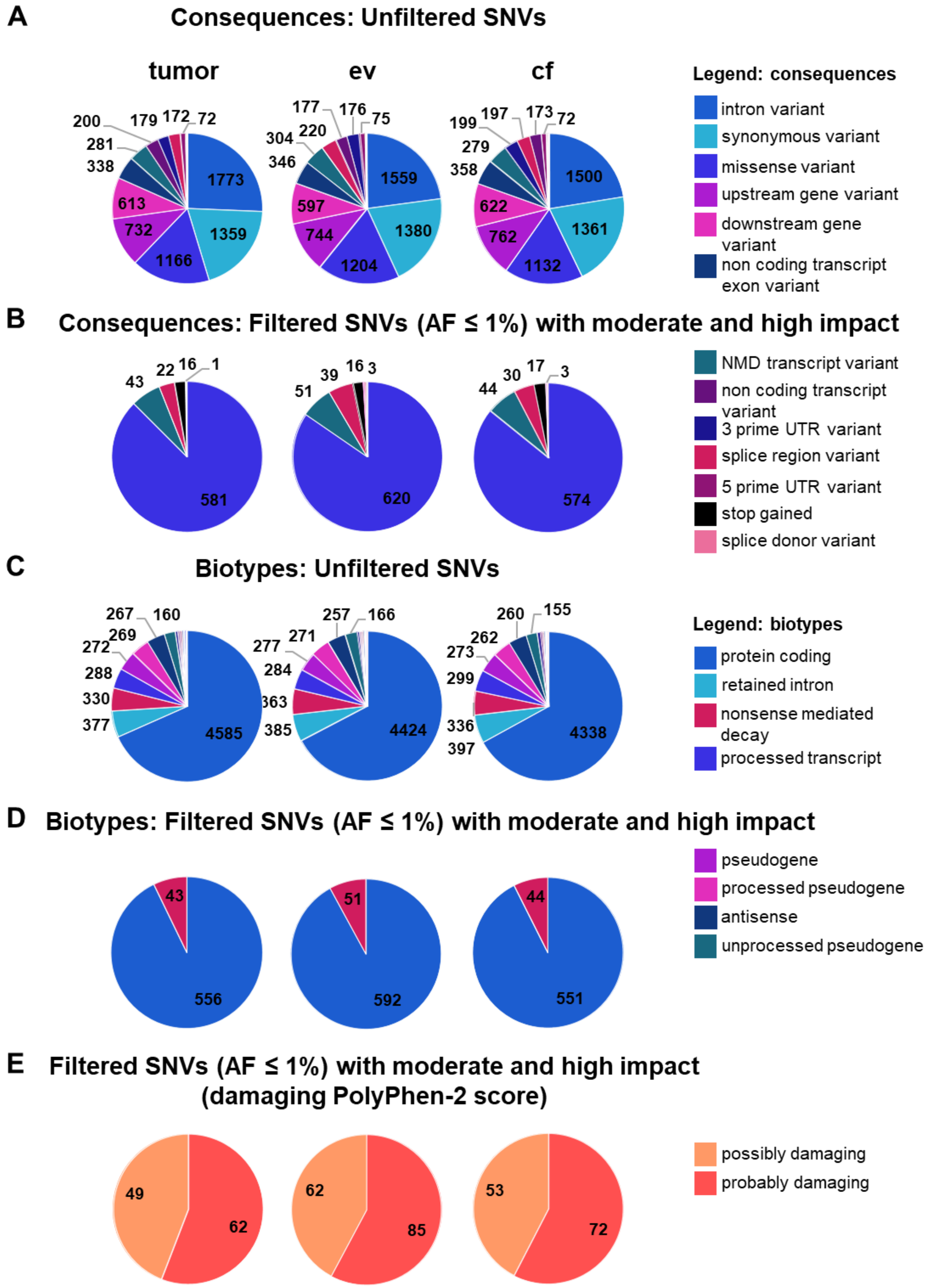

Supplemental Figure S19: Analysis of SNVs for patient 4. (A) Consequences of unfiltered SNVs and (B) filtered SNVs (AF≤1%) with moderate and high impact, (C) biotypes of unfiltered and (D) filtered SNVs (AF≤1%) with moderate and high impact as well as (D) number of variants with a possibly or probably damaging PP-2 score of filtered SNVs (AF≤1%) with moderate and high impact found in tumor-, ev- and cfDNA.

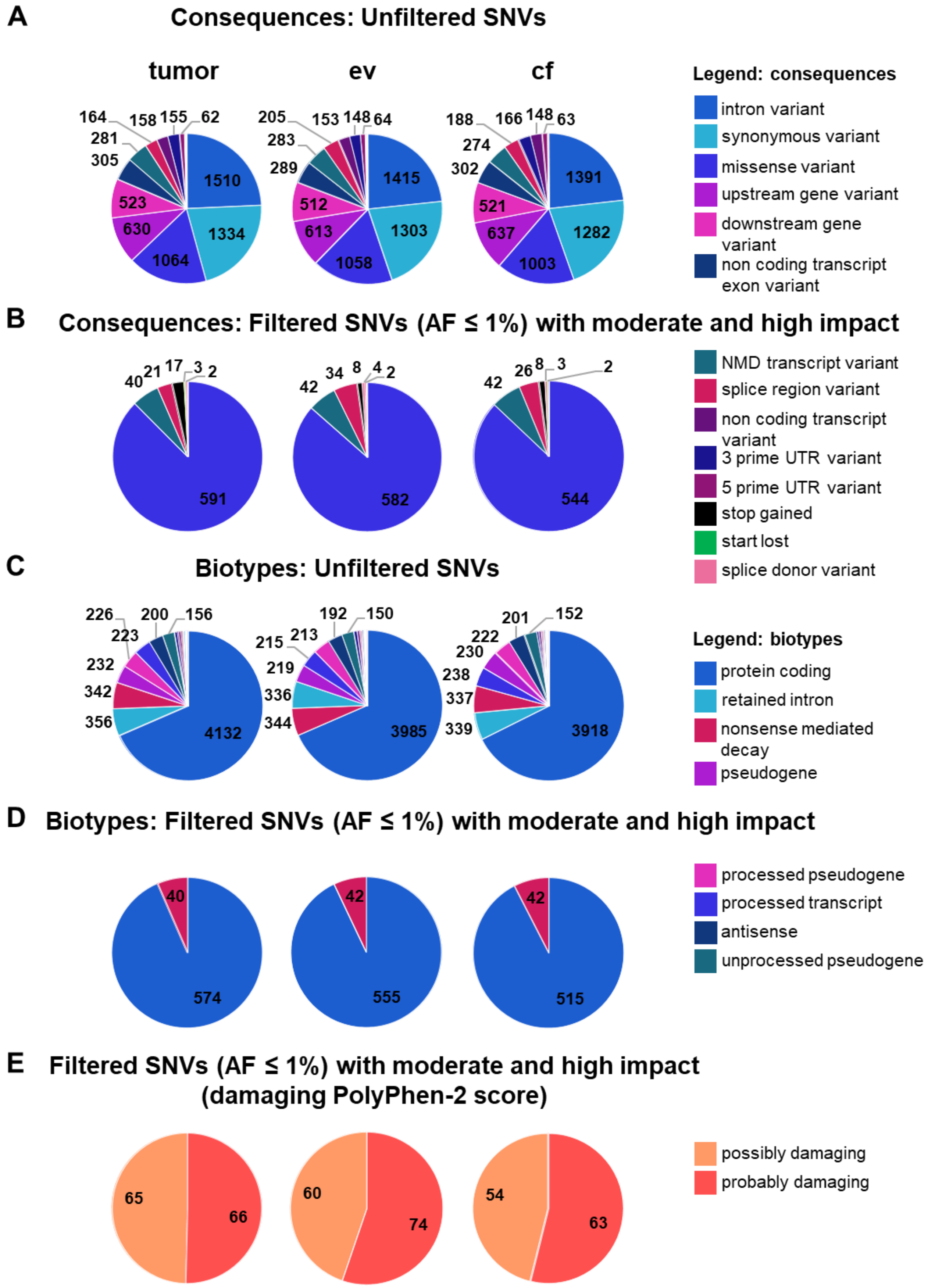

Supplemental Figure S20: Analysis of SNVs for patient 5. (A) Consequences of unfiltered SNVs and (B) filtered SNVs (AF≤1%) with moderate and high impact, (C) biotypes of unfiltered and (D) filtered SNVs (AF≤1%) with moderate and high impact as well as (D) number of variants with a possibly or probably damaging PP-2 score of filtered SNVs (AF≤1%) with moderate and high impact found in tumor-, ev- and cfDNA.

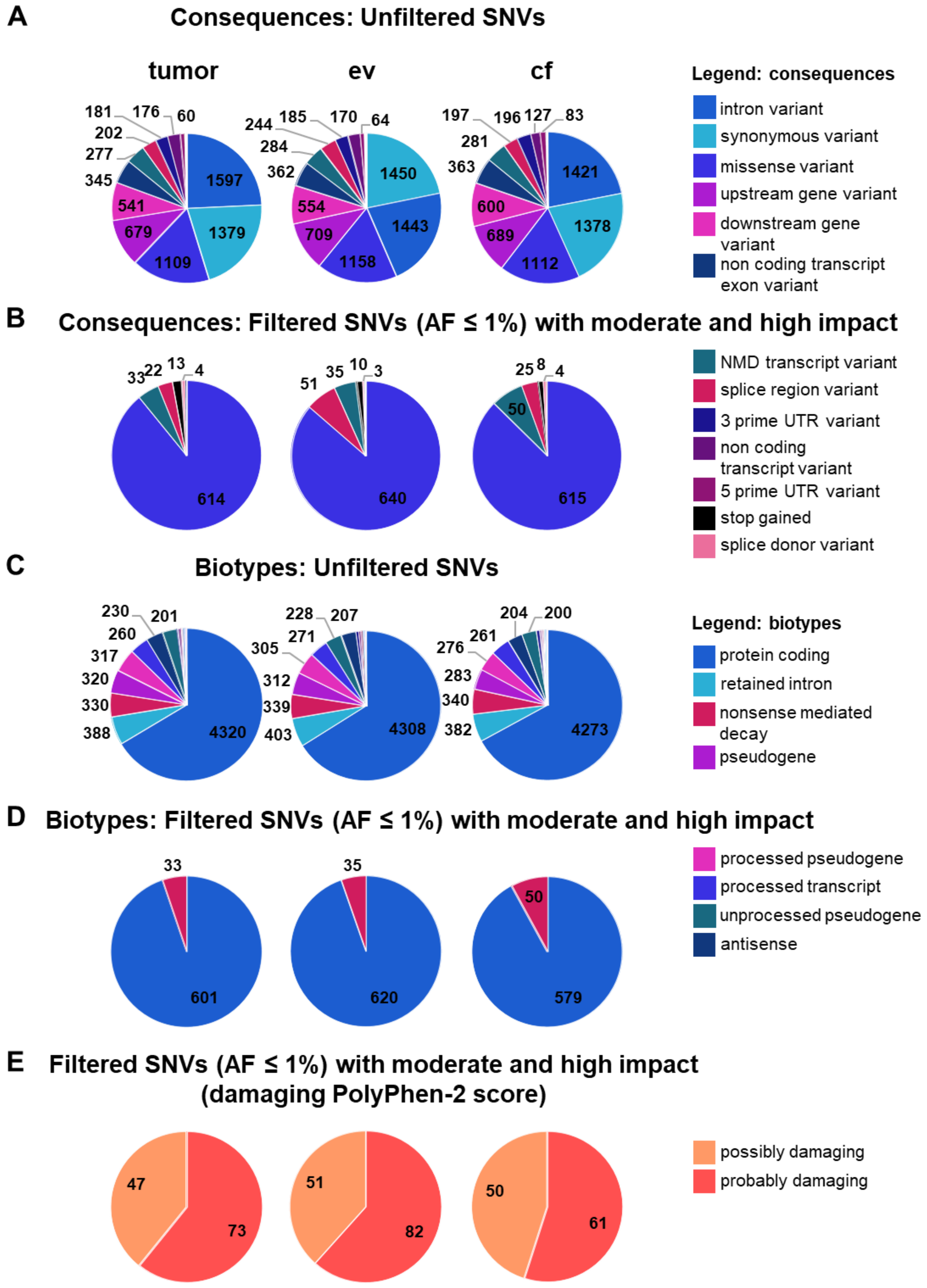

Supplemental Figure S21: Analysis of SNVs for patient 6. (A) Consequences of unfiltered SNVs and (B) filtered SNVs (AF≤1%) with moderate and high i mpact, (C) biotypes of unfiltered and (D) filtered SNVs (AF≤1%) with moderate and high impact as well as (D) number of variants with a possibly or probably damaging PP-2 score of filtered SNVs (AF≤1%) with moderate and high impact found in tumor-, ev- and cfDNA.

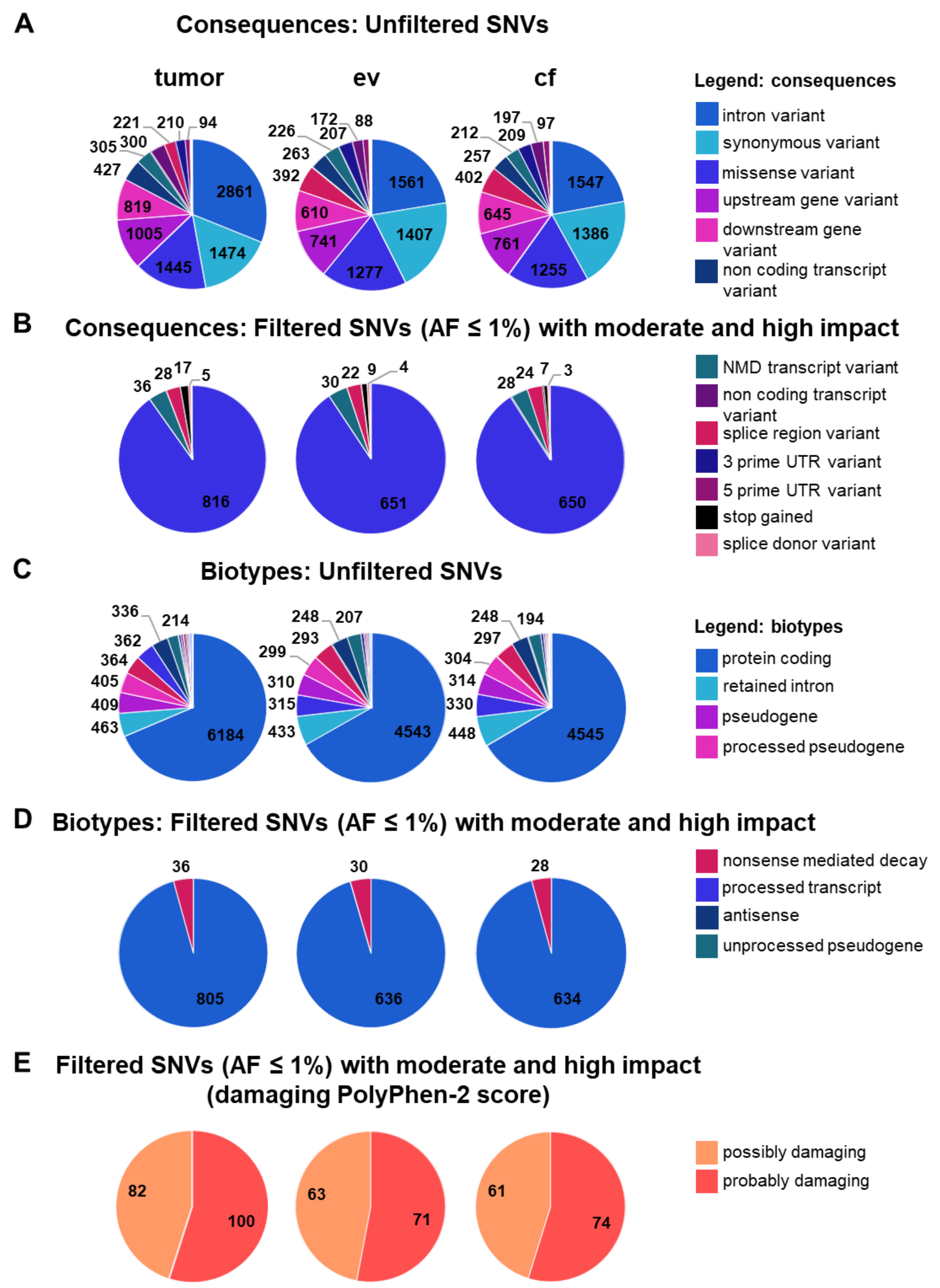

Supplemental Figure S22: Analysis of SNVs for patient 7. (A) Consequences of unfiltered SNVs and (B) filtered SNVs (AF≤1%) with moderate and high impact, (C) biotypes of unfiltered and (D) filtered SNVs (AF≤1%) with moderate and high impact as well as (D) number of variants with a possibly or probably damaging PP-2 score of filtered SNVs (AF≤1%) with moderate and high impact found in tumor-, ev- and cfDNA.

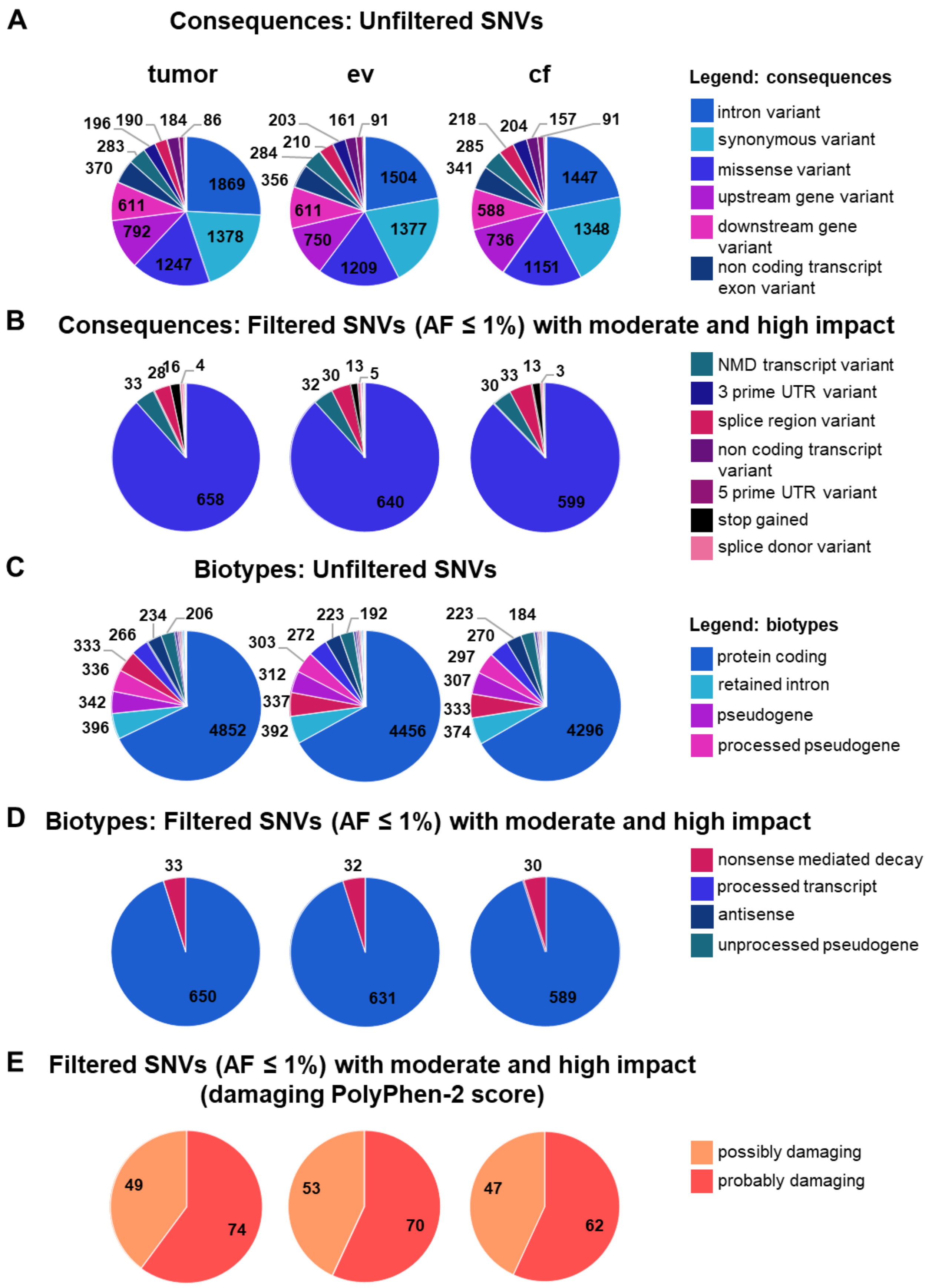

Supplemental Figure S23: Analysis of SNVs for patient 8. (A) Consequences of unfiltered SNVs and (B) filtered SNVs (AF≤1%) with moderate and high impact, (C) biotypes of unfiltered and (D) filtered SNVs (AF≤1%) with moderate and high impact as well as (D) number of variants with a possibly or probably damaging PP-2 score of filtered SNVs (AF≤1%) with moderate and high impact found in tumor-, ev- and cfDNA.

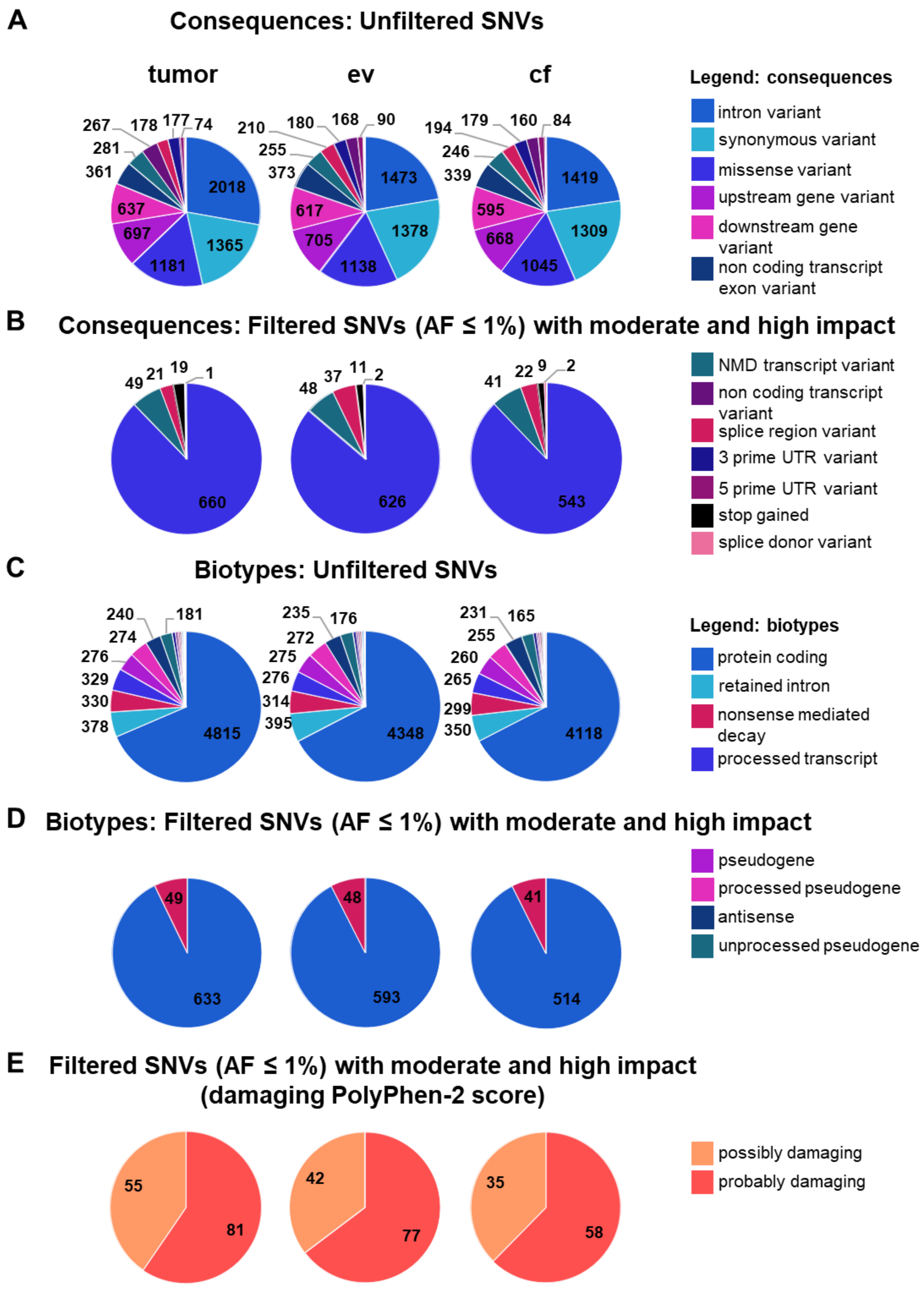

Supplemental Figure S24: Analysis of SNVs for patient 9. (A) Consequences of unfiltered SNVs and (B) filtered SNVs (AF≤1%) with moderate and high impact, (C) biotypes of unfiltered and (D) filtered SNVs (AF≤1%) with moderate and high impact as well as (D) number of variants with a possibly or probably damaging PP-2 score of filtered SNVs (AF≤1%) with moderate and high impact found in tumor-, ev- and cfDNA.

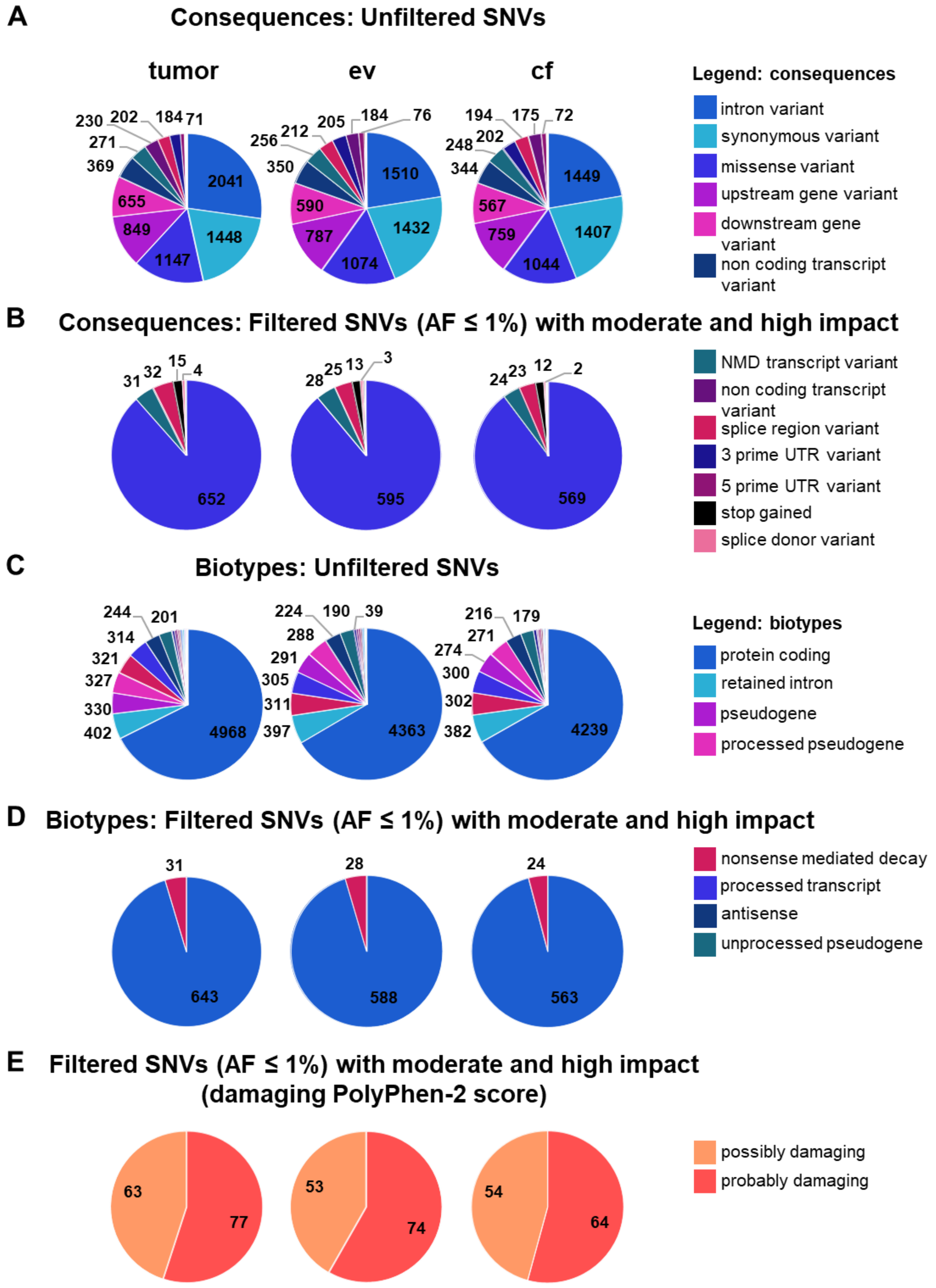

Supplemental Figure S25: Analysis of SNVs for patient 10. (A) ConsequencesofunfilteredSNVsand(B)filteredSNVs(AF≤1%) with moderate and high impact,(C)biotypesofunfilteredand(D)filteredSNVs(AF≤1%)withmoderateandhighimpact as well as (D) number of variants with a possibly or probably damaging PP-2scoreoffilteredSNVs(AF≤1%)withmoderate and high impact found in tumor-, ev- and cfDNA.

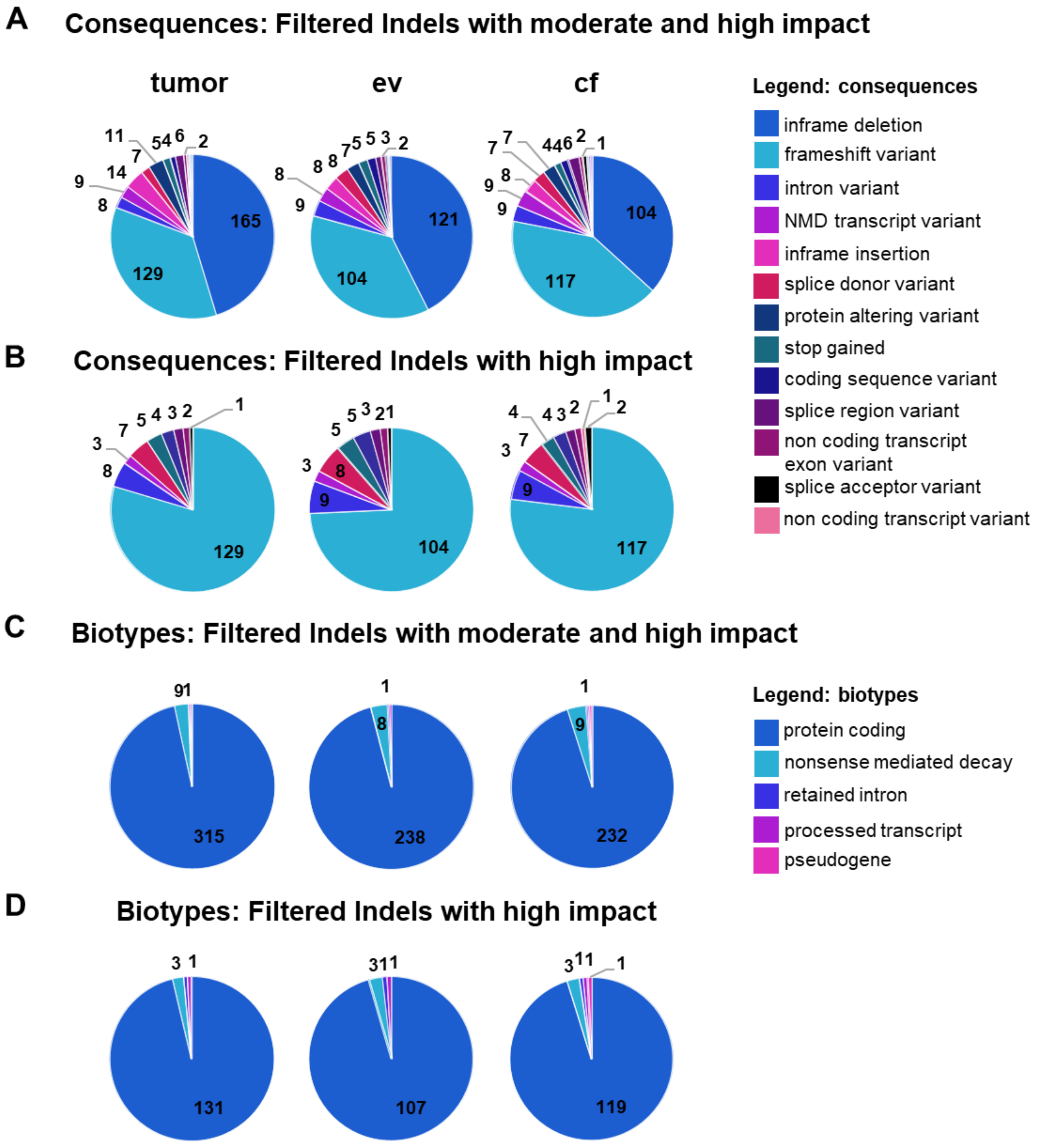

Supplemental Figure S26: Analysis of Indels for patient 1. (A) Consequences of Indels with moderate and high impact as well as (B) Indels with high impact. (C) Biotypes of Indels with moderate and high impact as well as (D) Indels with high impact found in tumor-, ev- and cfDNA.

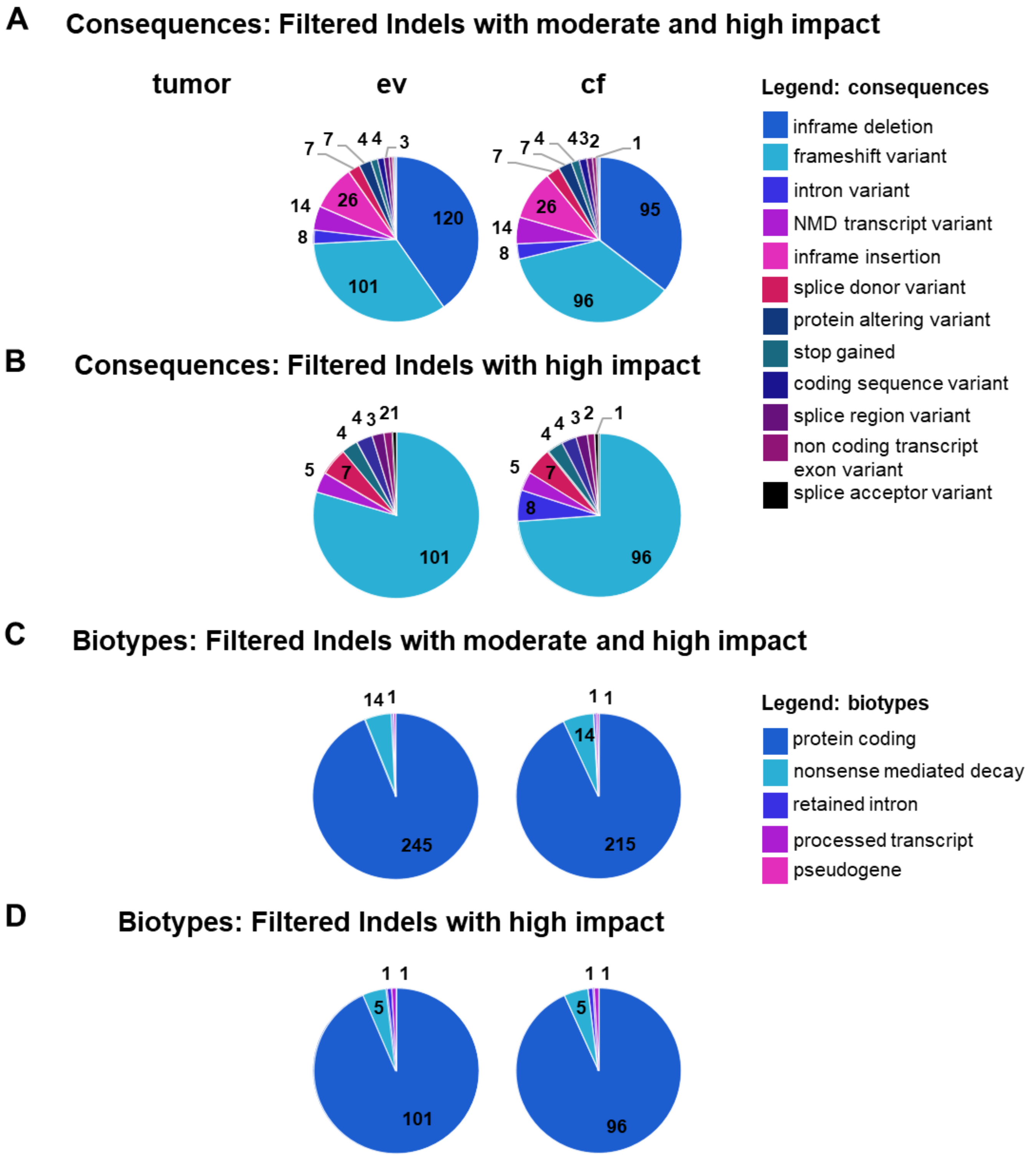

Supplemental Figure S27: Analysis of Indels for patient 2. (A) Consequences of Indels with moderate and high impact as well as (B) Indels with high impact. (C) Biotypes of Indels with moderate and high impact as well as (D) Indels with high impact found in tumor-, ev- and cfDNA.

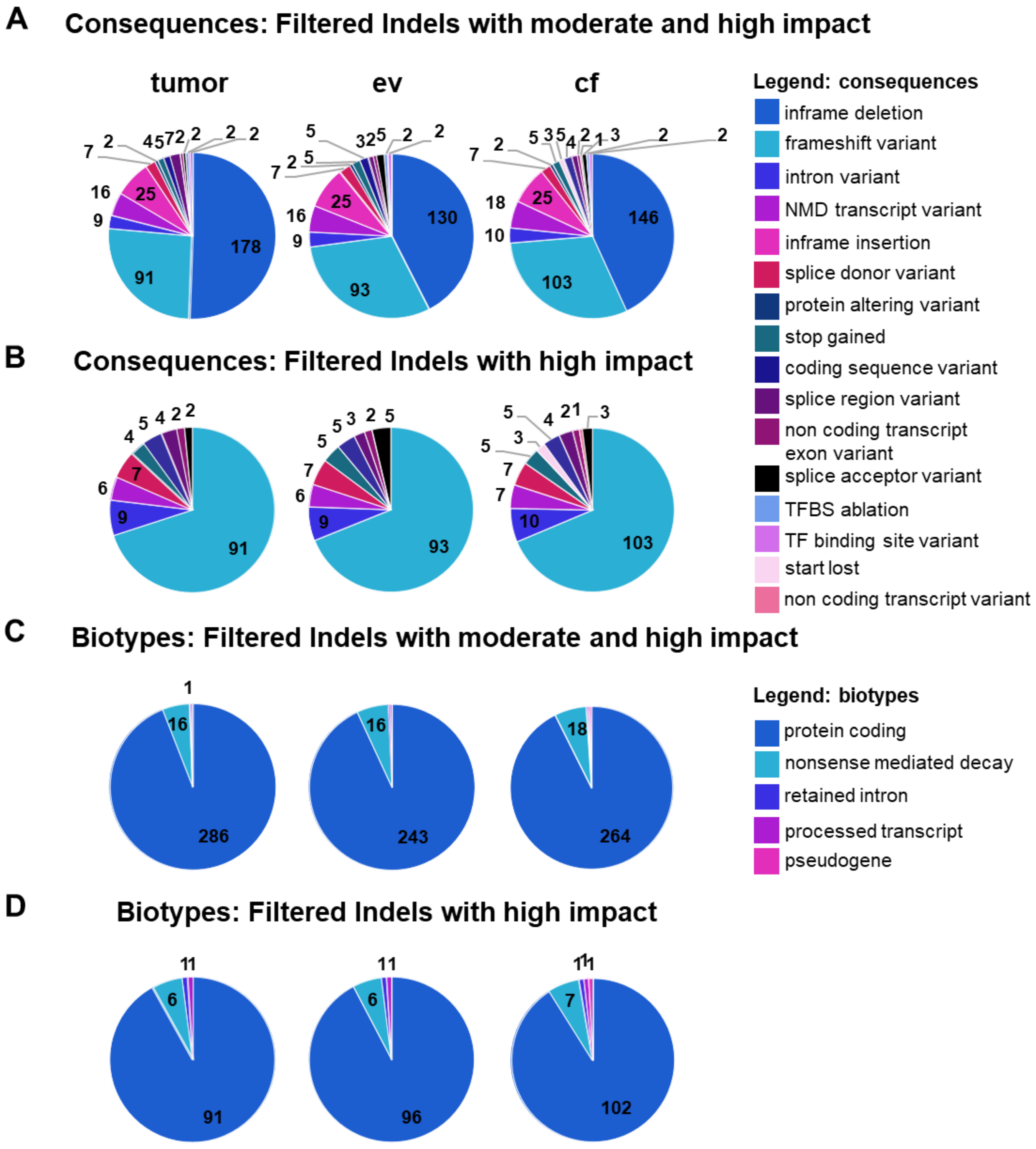

Supplemental Figure S28: Analysis of Indels for patient 3. (A) Consequences of Indels with moderate and high impact as well as (B) Indels with high impact. (C) Biotypes of Indels with moderate and high impact as well as (D) Indels with high impact found in tumor-, ev- and cfDNA.

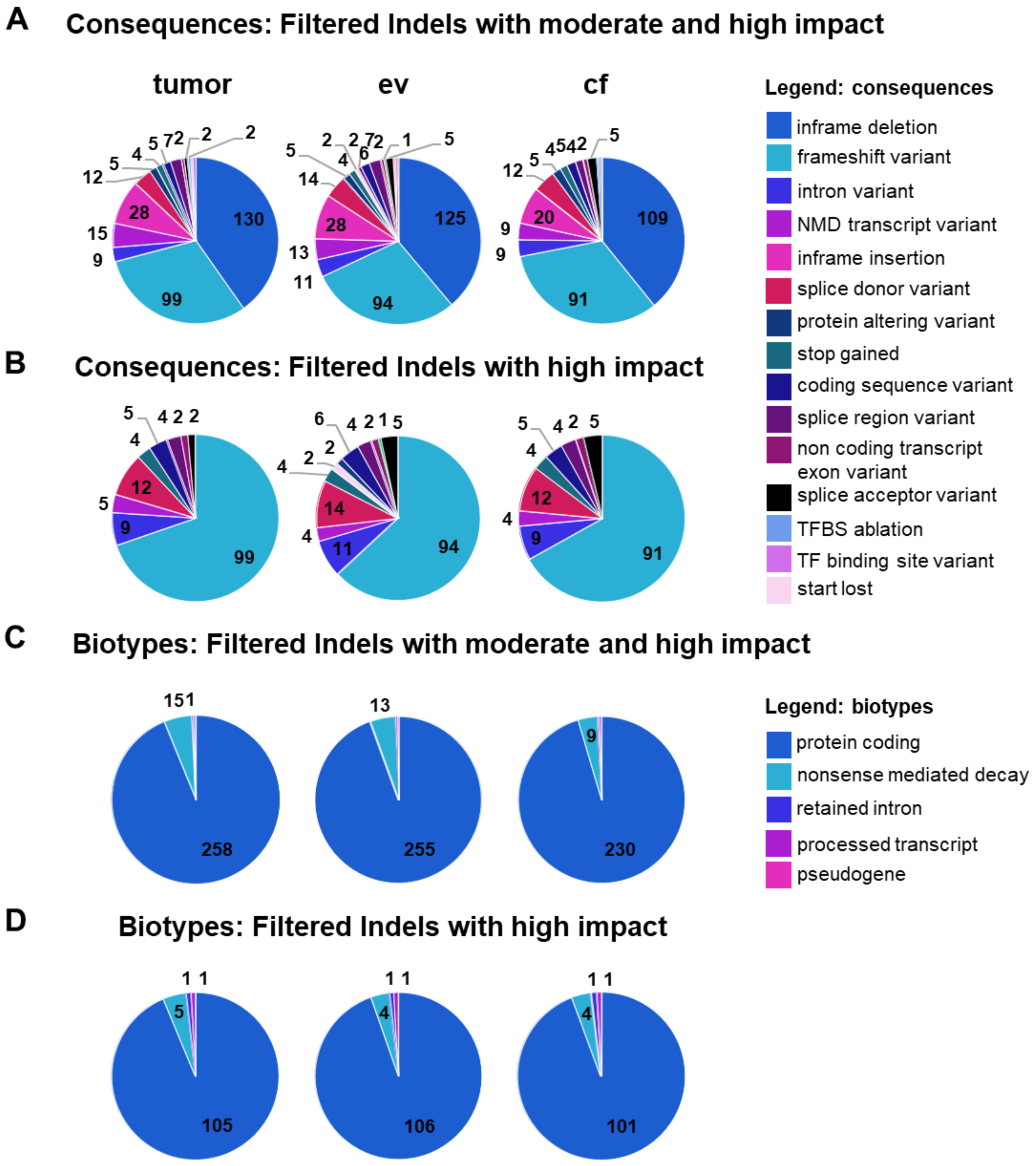

Supplemental Figure S29: Analysis of Indels for patient 4. (A) Consequences of Indels with moderate and high impact as well as (B) Indels with high impact. (C) Biotypes of Indels with moderate and high impact as well as (D) Indels with high impact found in tumor-, ev- and cfDNA.

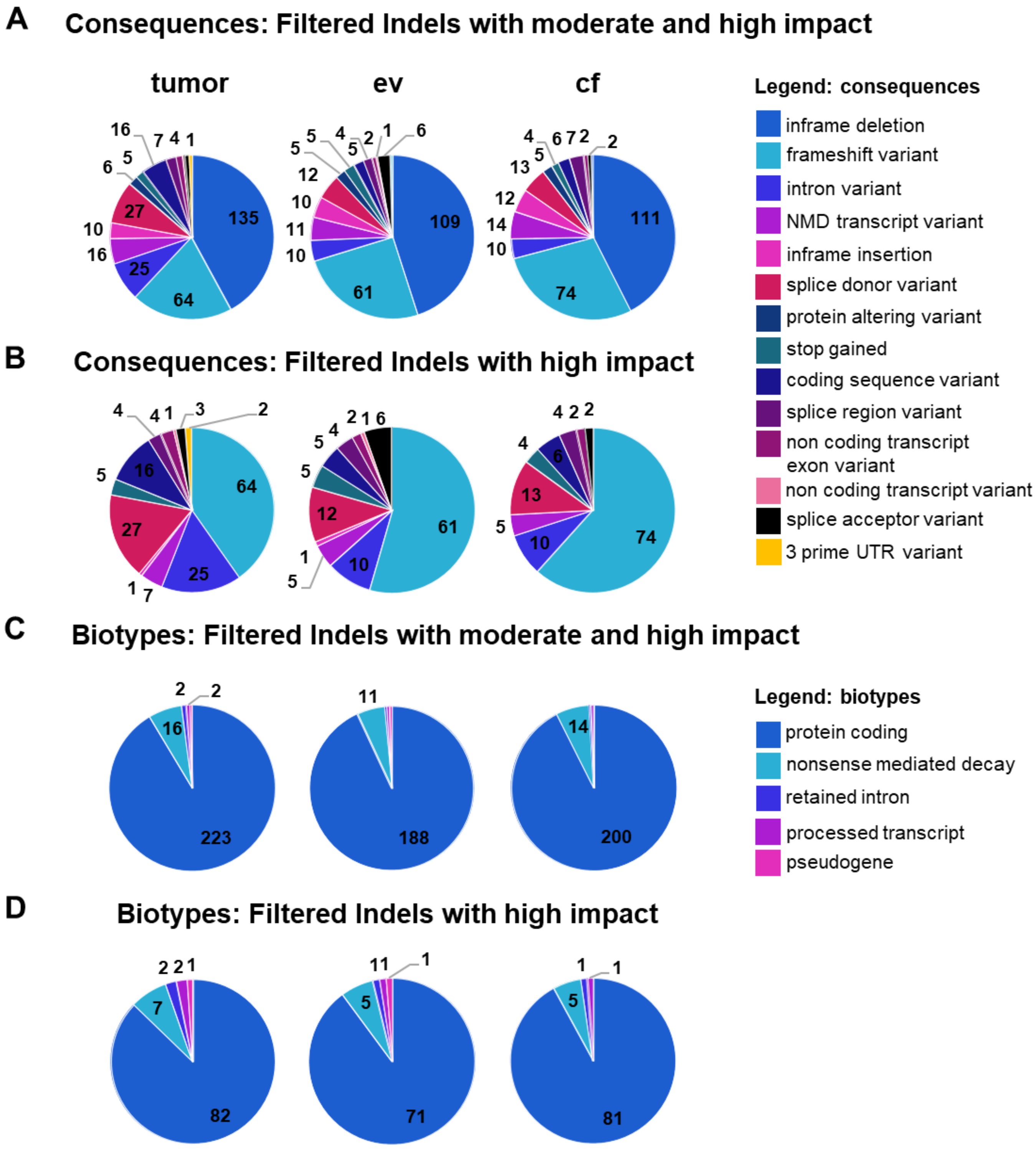

Supplemental Figure S30: Analysis of Indels for patient 5. (A) Consequences of Indels with moderate and high impact as well as (B) Indels with high impact. (C) Biotypes of Indels with moderate and high impact as well as (D) Indels with high impact found in tumor-, ev- and cfDNA.

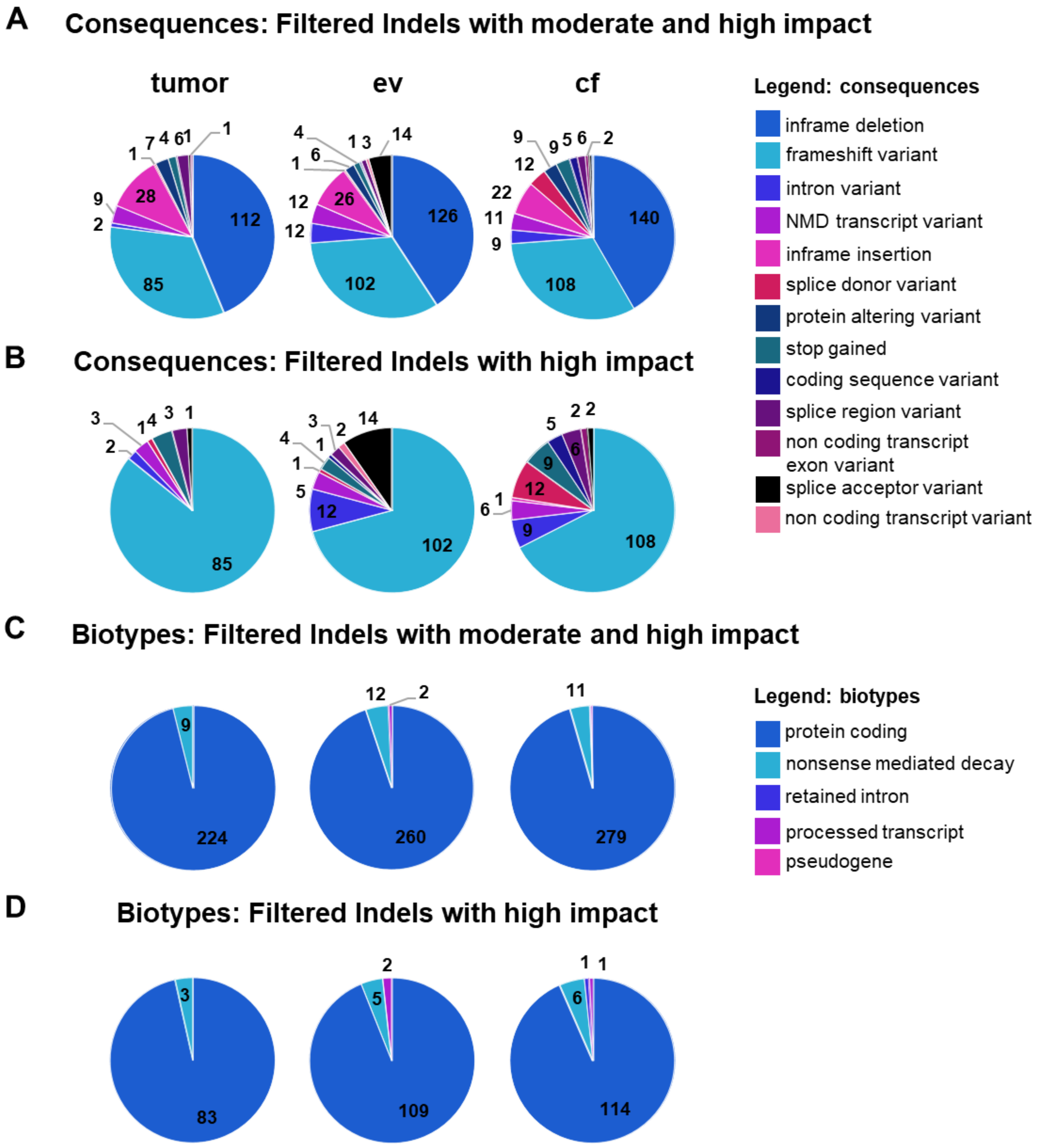

Supplemental Figure S31: Analysis of Indels for patient 6. (A) Consequences of Indels with moderate and high impact as well as (B) Indels with high impact. (C) Biotypes of Indels with moderate and high impact as well as (D) Indels with high impact found in tumor-, ev- and cfDNA.

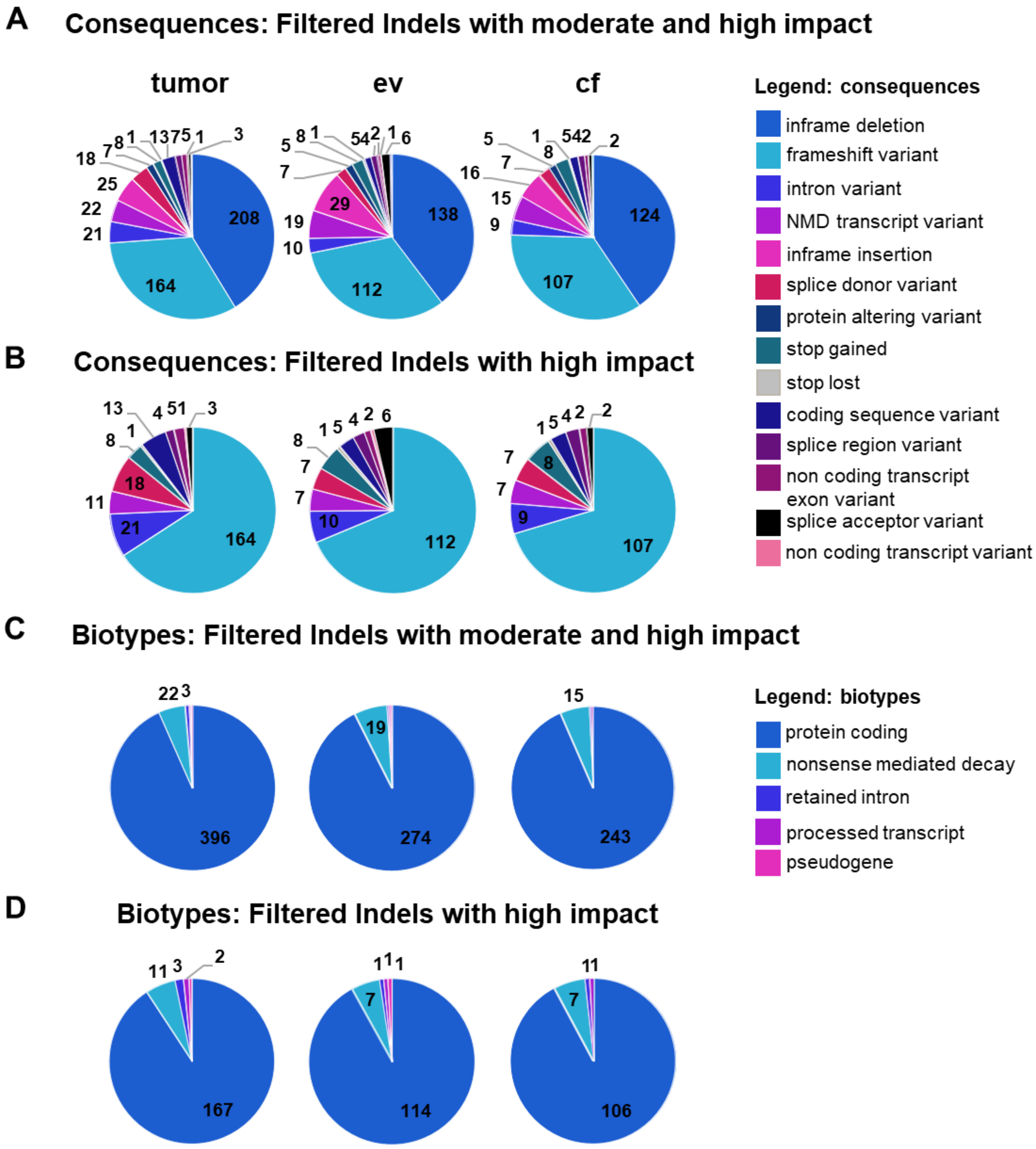

Supplemental Figure S32: Analysis of Indels for patient 7. (A) Consequences of Indels with moderate and high impact as well as (B) Indels with high impact. (C) Biotypes of Indels with moderate and high impact as well as (D) Indels with high impact found in tumor-, ev- and cfDNA.

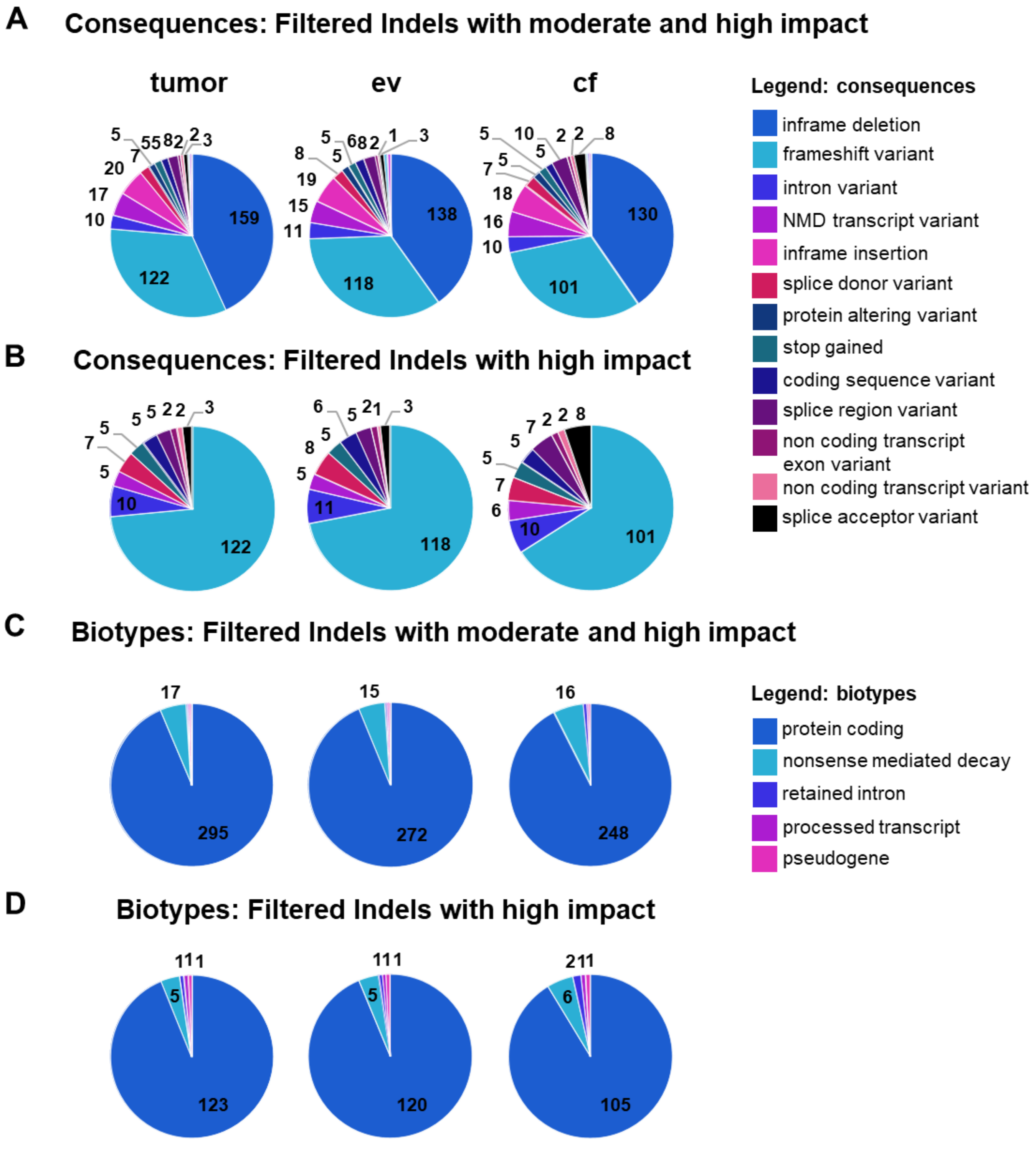

Supplemental Figure S33: Analysis of Indels for patient 8. (A) Consequences of Indels with moderate and high impact as well as (B) Indels with high impact. (C) Biotypes of Indels with moderate and high impact as well as (D) Indels with high impact found in tumor-, ev- and cfDNA.

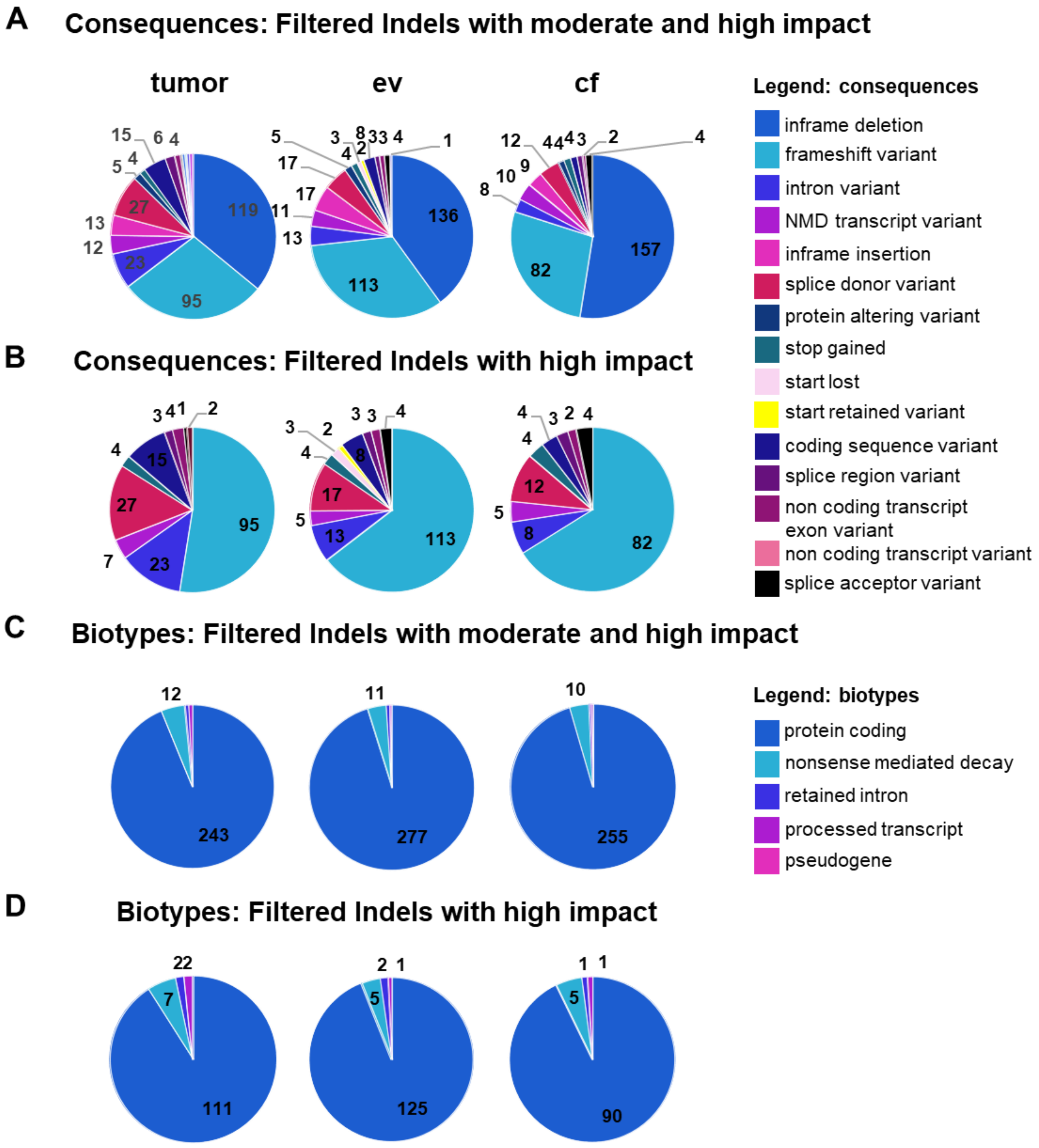

Supplemental Figure S34: Analysis of Indels for patient 9. (A) Consequences of Indels with moderate and high impact as well as (B) Indels with high impact. (C) Biotypes of Indels with moderate and high impact as well as (D) Indels with high impact found in tumor-, ev- and cfDNA.

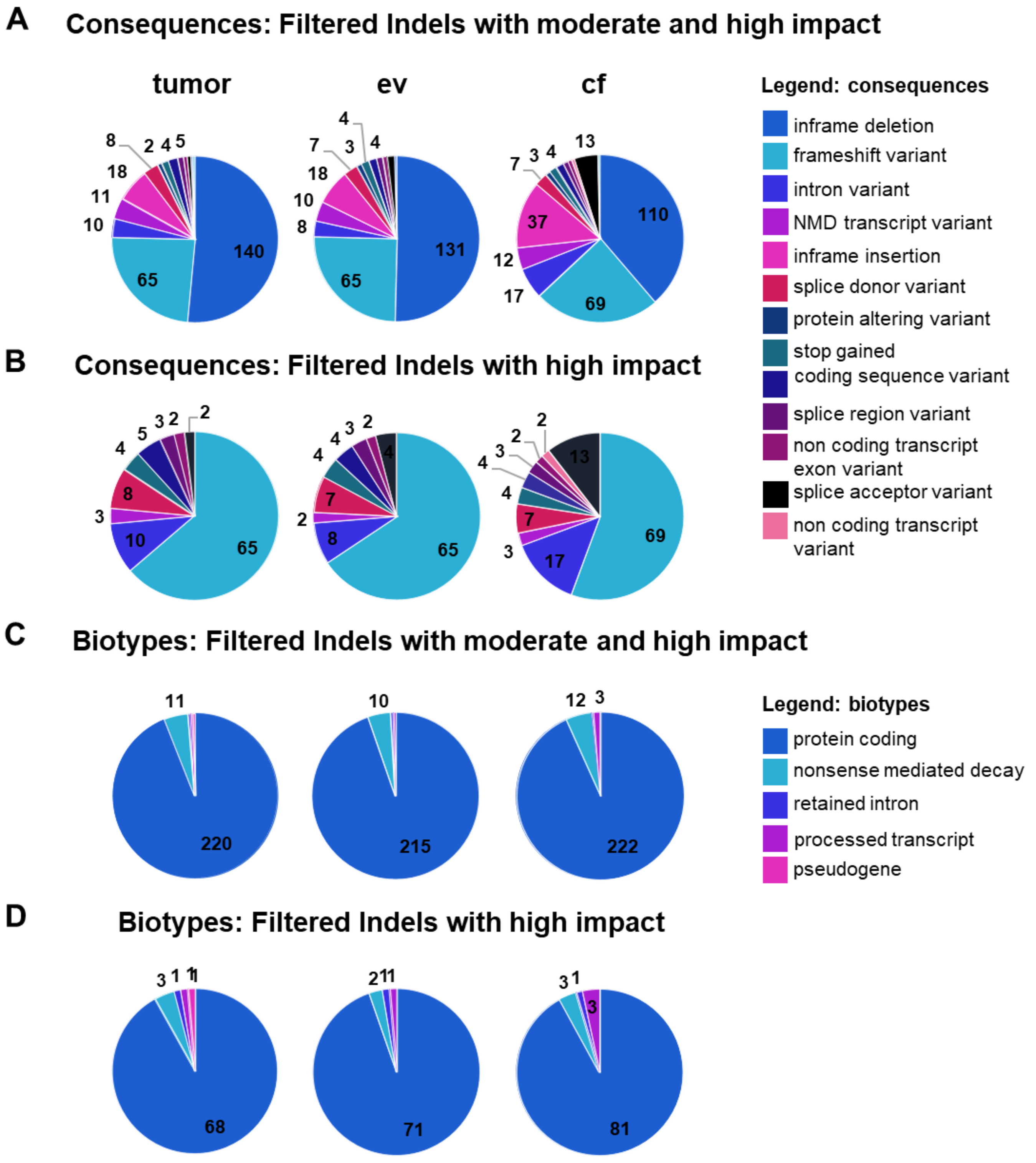

Supplemental Figure S35: Analysis of Indels for patient 10. (A) Consequences of Indels with moderate and high impact as well as (B) Indels with high impact. (C) Biotypes of Indels with moderate and high impact as well as (D) Indels with high impact found in tumor-, ev- and cfDNA.

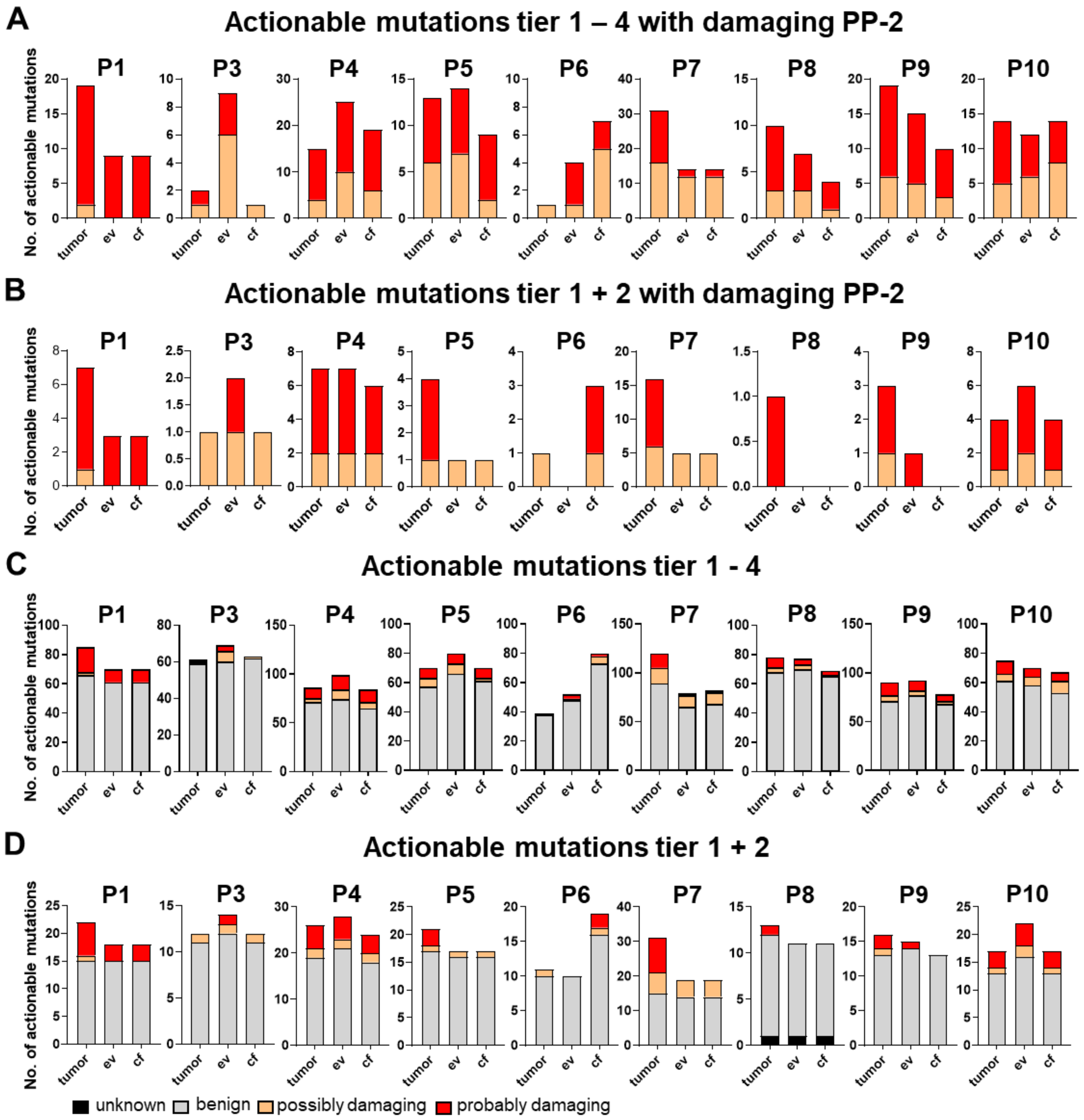

Supplemental Figure S36: Analysis of actionable variants. Absolute numbers of actionable variants with damaging PP-2 score (A) tier 1-4 and (B) tier 1+2 as well as absolute numbers of all PP-2 classified actionable variants (C) tier 1-4 and (D) tier 1+2 found in filtered SNVs with moderate and high impact of tumor-, ev- and cfDNA per patient.

Western Blot raw data

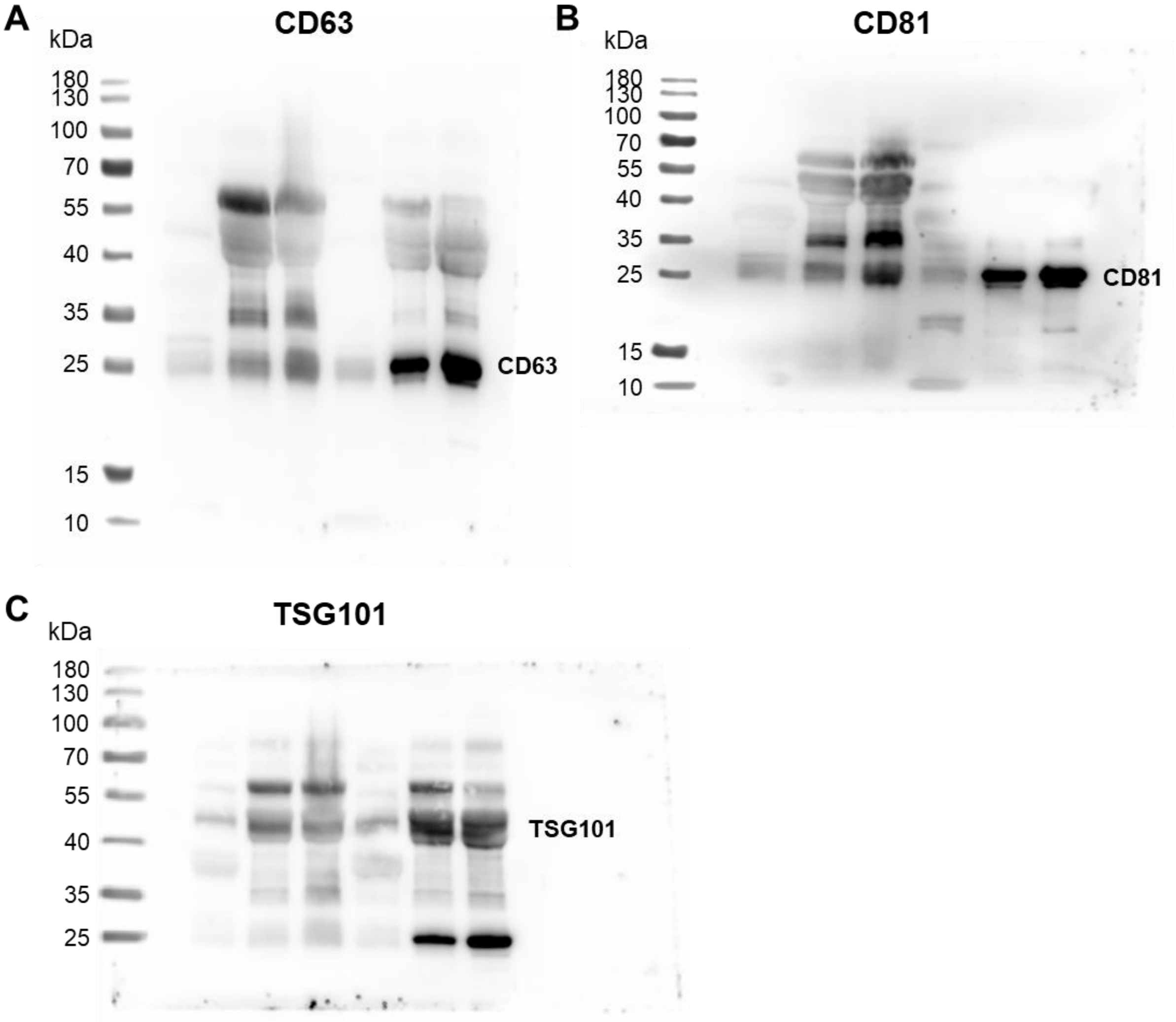

Supplemental Figure S37: Western Blot raw data showing all bands and molecular weight markers. (A) Raw data of CD63 blot. (B) Raw data of CD81 blot. (C) Raw data of TSG101 blot.
